# Single‐Cell Transcriptomics Reveals FLS2‐Dependent Hypoxia Signaling and ERF13‐Mediated Transcription During flg22‐Triggered Immunity

**DOI:** 10.1002/advs.202516380

**Published:** 2026-03-09

**Authors:** Yaping Zhou, Aizhi Qin, Mengfan Li, Qianli Zhao, Luyao Kong, Lulu Yan, Chunyang Li, Hao Liu, Yinpeng Zhang, Jiani Long, Mengyu Liao, Mengmeng Zhou, Xiaoli Fan, Baozhen Wang, Wenxuan Kang, Shui Wang, Zhixin Liu, Xuwu Sun

**Affiliations:** ^1^ National Key Laboratory of Cotton Bio‐breeding and Integrated Utilization State Key Laboratory of Crop Stress Adaptation and Improvement Key Laboratory of Plant Stress Biology School of Life Sciences Henan University 85 Minglun Street Kaifeng Henan China; ^2^ College of Life Sciences Shanghai Normal University Shanghai China

**Keywords:** ERF13, FLS2, Hypoxic signaling, ScRNA‐seq, Transcription factor network

## Abstract

The flagellin peptide flg22 activates FLAGELLIN‐SENSING 2 (FLS2)‐mediated immunity in Arabidopsis, leading to growth inhibition and oxidative burst. While these responses are well‐studied, their cell‐type‐specific regulation remains poorly understood. Using single‐cell RNA sequencing, genetics, and phenotyping, we systematically mapped flg22‐induced responses. flg22 suppressed growth and elevated reactive oxygen species (ROS) in wild‐type, but not in *fls2* mutants. Epidermal (EP_3) and mesophyll (MPC_2) cells showed FLS2‐dependent transcriptional reprogramming. Pseudotime analysis revealed developmental trajectories toward immune‐activated states. flg22 also induced a hypoxia‐like response; hypoxic signaling mutants (*ate1*, *prt6*, *zpr2*) showed reduced flg22 sensitivity, indicating crosstalk between immune and hypoxia pathways. ERF13 was identified as a central regulator: *erf13* mutants impaired flg22‐triggered ROS and growth inhibition but enhanced effector‐triggered immunity (ETI), while overexpressors showed stronger pattern‐triggered immunity (PTI). flg22 altered ploidy and cell‐cycle gene expression in WT, which was stabilized in *ate1* and *erf13* mutants. Cell‐cycle mutants *sim smr* and *e2fabc* enhanced flg22 responses, whereas *cpr5* was less sensitive. Thus, immune, hypoxia, and ROS signals converge via ERF13 to balance immunity and growth, providing a single‐cell view of spatial immune organization and stress adaptation.

## Graphical abstract

This study employs sc‐RNA sequencing, genetics, and phenotyping to systematically map the cell‐type‐specific immune responses triggered by flg22. It reveals FLS2‐dependent transcriptional reprogramming in epidermal and mesophyll cells, and uncovers crosstalk between immune and hypoxia signaling pathways. The transcription factor ERF13 is identified as a central integrator, converging immune, ROS, and cell‐cycle signals to balance growth and defense.

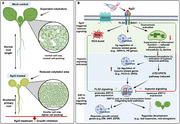

## 1 Introduction

Plants employ a multi‐layered receptor and signal transduction network to defend against pathogen invasion. Two primary immune receptor systems underpin this defense: cell surface‐localized pattern recognition receptors (PRRs, including receptor‐like kinases [RLKs]) and intracellular nucleotide‐binding oligomerization domain‐like receptors (NLRs) [1, 2, 3]. PRRs recognize pathogen‐associated molecular patterns (PAMPs) or damage‐associated molecular patterns (DAMPs), triggering pattern‐triggered immunity (PTI). NLRs detect pathogen effector proteins, activating the typically more robust effector‐triggered immunity (ETI) [4, 5].

Within PRR‐mediated immunity, FLAGELLIN‐SENSING 2 (FLS2) serves as a canonical receptor that specifically recognizes bacterial flagellin (e.g., the epitope flg22). FLS2 forms a complex with the co‐receptor BRI1‐ASSOCIATED RECEPTOR KINASE 1 (BAK1) to activate downstream signaling [6]. This process involves key components like the receptor‐like cytoplasmic kinase BRASSINOSTEROID‐SIGNALING KINASE1 (BSK1), whose interaction with FLS2 positively regulates* Arabidopsis* innate immunity [7]. Conserved tyrosine residues in SOMATIC EMBRYOGENESIS RECEPTOR KINASE (SERK) family proteins (e.g., AtSERK3/BAK1) specifically distinguish FLS2‐mediated from BRI1‐mediated signaling [8].

FLS2 signaling exhibits complex spatiotemporal control. Immune function is suppressed during early aerial tissue development, potentially linked to developmental signaling network restructuring [9]. Molecularly, phosphorylation of FLS2 at Ser‐938 alters receptor dynamics; single‐particle tracking shows this modification prolongs plasma membrane residence time, promotes homodimer formation, but does not enhance co‐localization with unrelated receptors [10]. Such regulation may involve partitioning FLS2 into specific membrane nanodomains (e.g., Remorin1.3 [Rem1.3]‐associated domains) [10]. Dynamin‐related proteins (e.g., DRP1A, DRP2B) modulate FLS2 distribution and signaling by influencing membrane lipid environments [10].

FLS2 signaling intricately interacts with hormone networks. FLS2 activation reprograms ∼20% of the transcriptome via NONEXPRESSER OF PR GENES1 (NPR1) in the salicylic acid (SA) pathway to establish systemic acquired resistance (SAR) [11, 12, 13]. NPR1 also mediates stomatal defense [14]. Jasmonic acid (JA) pathway interactions are bidirectional: BRASSINOSTEROID‐SIGNALING KINASE7/8 (BSK7/8) enhances flg22‐induced immunity [15], while the chromatin factor GENERAL TRANSCRIPTION FACTOR GROUP E4 (GTE4) promotes SA signaling by suppressing JA biosynthesis [16]. This cross‐regulation shows spatiotemporal specificity [17]. FLS2 also cross‐regulates with SA; SA‐induced CLAVATA3/ESR‐RELATED 3 (CLE3) remotely regulates SAR via WRKY33 [18]. Collectively, plants achieve spatiotemporal defense control through receptor dynamics, membrane microdomain compartmentalization, and inter‐tissue signaling [19, 20, 21].

Regarding NLR‐mediated immunity, recent studies reveal novel mechanisms where NLRs precisely modulate immune signals via macromolecular assembly and phase separation [22]. This oligomerization acts as a critical “switch” for receptor activation [22]. Different NLRs employ distinct mechanisms, including resistosome formation [23]. Notably, NLRs recognize effectors *and* regulate hormone pathways, with the abscisic acid (ABA) pathway playing a pivotal defense role [24].

Plant tissues and cells exhibit marked functional specialization. Epidermal and mesophyll cells employ complementary defense strategies [25, 26, 27]. Epidermal cells, the primary physical barrier, rapidly activate structural defenses (cell wall lignification, wax deposition) and secrete antimicrobial metabolites (e.g., phenolic acids, flavonoids) [25, 26, 27]. Single‐cell transcriptomics shows epidermal cells significantly upregulate defense genes, including key pathways like ENHANCED DISEASE SUSCEPTIBILITY1 (EDS1)/PHYTOALEXIN‐DEFICIENT4 (PAD4) and PENETRATION2 (PEN2)/PEN3 [28]. In contrast, mesophyll cells reprogram metabolism toward defense compound synthesis by suppressing photosynthesis‐related genes and may trigger localized hypersensitive response (HR) cell death [29, 30, 31]. The MITOGEN‐ACTIVATED PROTEIN KINASE3 (MPK3)/MPK6 pathway exhibits differential regulation: modulating stomatal immunity in epidermis, and balancing leaf development with immunity in mesophyll cells [32]. This division of labor maintains essential physiology while achieving effective immunity.

Notably, plants exhibit complex immune‐hypoxia interactions under stress. Hypoxia causes abnormal reactive oxygen species (ROS) accumulation, triggering oxidative stress and lipid peroxidation [33]. Multilayered regulatory mechanisms exist; ETHYLENE RESPONSE FACTOR VII (ERFVII) transcription factors (e.g., AtERF73/HRE1) coordinate hypoxia‐responsive gene expression [34, 35]. Intriguingly, the Arg/N‐degron pathway (mediated by E3 ligases like PROTEOLYSIS6 [PRT6]) shows significant crosstalk between hypoxia signaling and flg22‐induced PTI [36]. By modulating ERFVII stability (∼20% of hypoxia‐responsive genes), this pathway integrates environmental stress and pathogen defense signals [37, 38], revealing how plants balance abiotic stress and immunity.

Hypoxic stress and FLS2‐mediated immunity influence plant growth, yet signaling pathways regulating cell‐type‐specific developmental dynamics remain unclear. A complex trade‐off exists between immunity and growth, potentially mediated by hormone networks and cell‐specific responses [9, 11, 15]. During seedling development, FLS2 pathway activation significantly impacts shoot morphogenesis, indicating precise spatiotemporal regulation between immunity and development [9]. However, systematic understanding of immune stress effects on developmental dynamics across cell types and their regulatory networks is lacking.

To elucidate FLS2‐mediated immune regulation of cellular development, this study employs single‐cell transcriptomics to systematically compare developmental dynamics in wild‐type (WT) and *fls2 *mutant *Arabidopsis* seedlings under flg22 treatment. Using 10× Genomics for high‐resolution single‐cell data and Uniform Manifold Approximation and Projection (UMAP) analysis, we dissect immune activation‐induced transcriptional reprogramming across cell types, analyze FLS2‐dependent developmental trajectory changes, and construct key regulatory networks. Functional validation of critical regulators provides insights into maintaining growth homeostasis under immune stress [39].

## 2 Results

### 2.1 Flg22 Suppresses *Arabidopsis* Seedling Growth via FLS2‐Dependent Oxidative Burst

To assess the impact of flg22‐induced immune responses on *Arabidopsis* development, we treated 3‐day‐old WT and *fls2* mutant seedlings with 1 µM flg22 or mock solution in liquid culture. After three days, we analyzed seedling growth phenotypes and ROS accumulation (Figure S1).

flg22 treatment severely inhibited WT seedling growth: primary root length decreased by ∼42%, cotyledon angle by 35%, and cotyledon area by 58% compared to mock‐treated controls (Figure S1A–E). In contrast, *fls2* mutant growth parameters remained unaffected (Figure S1A–E).

ROS detection revealed markedly increased staining intensity for nitroblue tetrazolium (NBT; superoxide indicator) and 3,3'‐diaminobenzidine (DAB; hydrogen peroxide indicator) in flg22‐treated WT cotyledons, indicating substantial O_2_^−^ and H_2_O_2_ accumulation (Figure S1F–I). This suggests disruption of intracellular redox homeostasis. ROS levels in *fls2* mutants were unchanged (Figure S1F–I). These results demonstrate that flg22 triggers an FLS2‐dependent oxidative burst, inducing oxidative stress concomitant with growth suppression.

### 2.2 Single‐Cell Transcriptomic Analysis of flg22‐Induced Immune Response

To elucidate the dynamic cellular developmental changes during *Arabidopsis* immune responses, we performed single‐cell RNA sequencing (scRNA‐seq) on cotyledons from WT and *fls2* seedlings, both with and without flg22 treatment. As the key nutrient organ facilitating the transition from heterotrophic to autotrophic growth in *Arabidopsis* seedlings, cotyledons provide essential nutrients for seedling development through photosynthesis [40]. Protoplasts isolated from the cotyledons of WT_CK, fls2_CK, WT_flg22, and fls2_flg22 were used to prepare single‐cell libraries, which were subsequently processed on the 10× Genomics platform. The raw sequencing data were subjected to stringent quality control (QC) (Figure S2; Table S1). Ultimately, based on stringent thresholds for nGene (detected genes per cell), nUMI (UMI counts), and percent_mito (mitochondrial gene percentage), a total of 77 909 high‐quality single‐cell transcriptomes were retained for downstream analyses.

**FIGURE 1:**
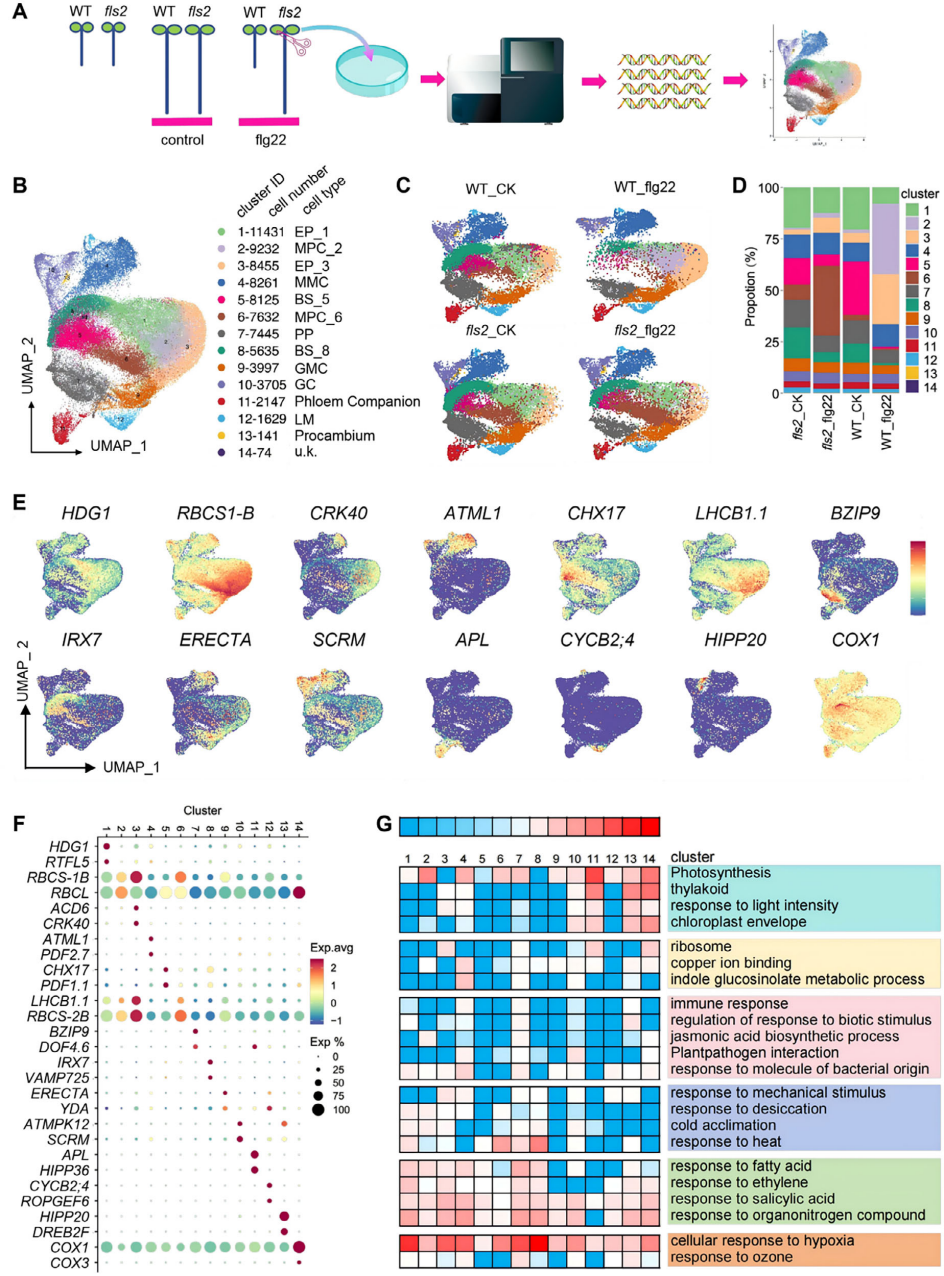
UMAP‐based clustering analysis and cell type identification in scRNA‐seq. (A) Schematic illustration of the experimental workflow. (B) UMAP projection of scRNA‐seq data from all four samples (WT_CK, WT_flg22, *fls2*_CK, and *fls2*_flg22), with 14 distinct cell clusters color‐coded. The x‐ and y‐axes represent the first and second principal components derived from dimensionality reduction, respectively, where each point corresponds to an individual cell. (C) UMAP plots displaying the four samples separately. (D) Stacked bar plot illustrating the proportional distribution of cells across different clusters within each sample. (E) Feature plot depicting the expression of representative marker genes for each cell cluster. (F) Dot plot showcasing the top two marker genes for each cluster. (G) Heatmap of Gene Ontology (GO) enrichment analysis for differentially expressed genes (DEGs) in each cell cluster.

Next we employed the UMAP algorithm for dimensionality reduction and clustering visualization of single‐cell populations, identifying a total of 14 characteristic cell clusters (Figure 1A,B), with the cellular distribution of each cluster shown in the Figure 1B. The observed variations in cluster cell proportions across the four experimental groups (WT_CK, fls2_CK, WT_flg22, and fls2_flg22) may reflect inherent variability introduced during the droplet‐based single‐cell capture step of library construction (Figure 1C,D; Table S2).

Cluster‐specific marker genes were identified (Table S3). Integrating expression patterns of known cell‐type markers (Tables S4 and S5) [41, 42, 43, 44, 45, 46, 47] with differential gene expression analysis enabled systematic cluster annotation (Figure 1B,E,F): Epidermal cells comprised two subpopulations: EP_1 (Cluster 1), marked by the homeobox gene *homeodomain GLABROUS 1* (*HDG1*), and EP_3 (Cluster 3), which expressed immune‐related genes *ACCELERATED CELL DEATH 6* (*ACD6*) and *CYSTEINE‐RICH RLK (RECEPTOR‐LIKE PROTEIN KINASE) 40* (*CRK40*). Mesophyll cells were represented by MPC_2 (Cluster 2) and MPC_6 (Cluster 6), both expressing the photosynthetic gene *LIGHT HARVESTING CHLOROPHYLL A/B‐BINDING PROTEIN 1.1* (*LHCB1.1*). Meristemoid mother cells (MMC, Cluster 4) expressed the key regulators *MERISTEM LAYER 1* (*ATML1*) and *PROTODERMAL FACTOR 2* (*PDF2*), while cortex cells consisted of two clusters: Cortex_5 (Cluster 5), expressing *CATION/H^+^ EXCHANGER 17* (*CHX17*), *SULFATE TRANSPORTER 1;2* (*SULTR1;2*), and *PLANT DEFENSIN 1.1* (*PDF1.1*), and Cortex_8 (Cluster 8), characterized by *IRREGULAR XYLEM 7* (*IRX7*) and *VESICLE‐ASSOCIATED MEMBRANE PROTEIN 725* (*VAMP725*). Vascular lineages included Protophloem cells (PP, Cluster 7) expressing *BASIC LEUCINE ZIPPER 9* (*bZIP9*) and *SWEET11*, Phloem Companion cells (Cluster 11) marked by *ALTERED PHLOEM DEVELOPMENT* (*APL*) and *HEAVY METAL‐ASSOCIATED ISOPRENYLATED PLANT PROTEIN 36* (*HIPP36*), and procambium cells (Cluster 13) expressing *HIPP20* and *DEHYDRATION‐RESPONSIVE ELEMENT BINDING PROTEIN 2F* (*DREB2F*). Stomatal lineage cells were identified as guard mother cells (GMC, Cluster 9), expressing *YDA* and *ERECTA*, and guard cells (GC, Cluster 10) expressing *SCREAM* (*SCRM*) and *MITOGEN‐ACTIVATED PROTEIN KINASE 12* (*MPK12*). Lateral meristem cells (LM, Cluster 12) were marked by *CYCLIN B2;4* (*CYCB2;4*) and *ROP* (*RHO OF PLANTS*) *GUANINE NUCLEOTIDE EXCHANGE FACTOR 6* (*ROPGEF6*), whereas Cluster 14 lacked definitive marker expression and was thus designated as “unknown”.

Functional enrichment analysis further corroborated the identity of these cell types and revealed a high degree of concordance between their transcriptional profiles and physiological roles (Figure 1G; Figure S3). Mesophyll cells (MPC_2 and MPC_6) exhibited significant enrichment in photosynthesis (ath00195) and thylakoid‐related pathways (GO:0009579), consistent with their expression of *LHCB1.1* and their role as primary sites of photosynthetic activity. Epidermal cells (EP_1 and EP_3) were enriched in aldehyde biosynthesis (GO:0046184) and high‐light response (GO:0009644), while also expressing immune‐related genes such as *ACD6* and *CRK40*, supporting their dual role in environmental sensing and barrier defense. Meristematic cells, including MMC and lateral meristem (LM), showed enrichment in chromatin organization (GO:0030527) and spliceosome activity (ath03040), in line with their proliferative state. Vascular cells—protophloem and phloem—were markedly enriched in membrane transport and generation of precursor metabolites and energy (GO:0006091), aligning with their function in nutrient translocation. Notably, carbon metabolism (ath01200) and energy‐related pathways were specifically upregulated in mesophyll cells, whereas immune response processes (GO:0002376) were more active in epidermal and cortical cells, reflecting a clear functional division of labor in both metabolic specialization and environmental adaptation. Collectively, these results demonstrate, at single‐cell resolution, a precise alignment between cellular identity and physiological function in plant tissues.

To validate the cell type‐specific expression of the candidate genes identified in this study, we performed subcellular localization analysis for* PS II OXYGEN‐EVOLVING COMPLEX 1* (*PSBO1)* (*AT5G66570*, from MPC_2) and* EPIDERMAL PATTERNING FACTOR LIKE‐9* (*EPFL9) (AT4G12970)* (from EP_3). Confocal imaging revealed that PSBO1‐GFP localized predominantly in the mesophyll region, with signals observed in chloroplasts, while EPFL9‐GFP was expressed in both epidermis (EP) and mesophyll cells (MPC) (Figure S4). Although EPFL9 is considered a mesophyll‐derived secreted peptide [48]. Our results demonstrate that* EPFL9* is also expressed in clusters related to epidermal development, including EP_1, EP_3, guard mother cells (GMC), and lateral meristemoid (LM)—all of which are associated with the epidermal domain. Consistent, in the Table S2 of Guo et al., (2025), we found that *EPFL9* is expressed in both epidermal cells and mesophyll cells at the early development stage 1 of rosette leaf [47]. Similarly, in the study by Xia et al. (2023), analysis revealed that the *EPFL9* gene is specifically expressed mainly in the lower epidermal cells, while its expression was not identified among the differentially expressed genes (DEGs) in mesophyll cells [42]. Therefore, these results show the possibility of *EPFL9*’s mRNA expression in epidermal cells or its application as a marker gene for epidermal cells.

Furthermore, to validate the reliability of the identified cell types, we performed a comparative analysis with previously published gene sets specifically expressed in MPC, EP, GC, PP, Phloem Companion, Procambium, and Cortex (Table S5). Our results showed that MPC_2 and MPC_6 shared 369 and 376 genes, respectively, with these reference sets [42]. For Cortex_5 and Cortex_8, we identified 295 and 503 overlapping genes, respectively [41]. In the case of EP_1 and EP_3, 28 and 41 shared genes were detected, respectively [42]. For GC, PP, Phloem Companion, and Procambium, we identified 267, 294, 215, and 217 overlapping genes, respectively [43]. A dot plot in Figure S5 illustrates the expression patterns of selected representative genes across different clusters. Based on these cross‐study comparisons, we further confirmed the identity of the cell types identified in our study.

### 2.3 Developmental Trajectory Variations across Different Cell Types

**FIGURE 2:**
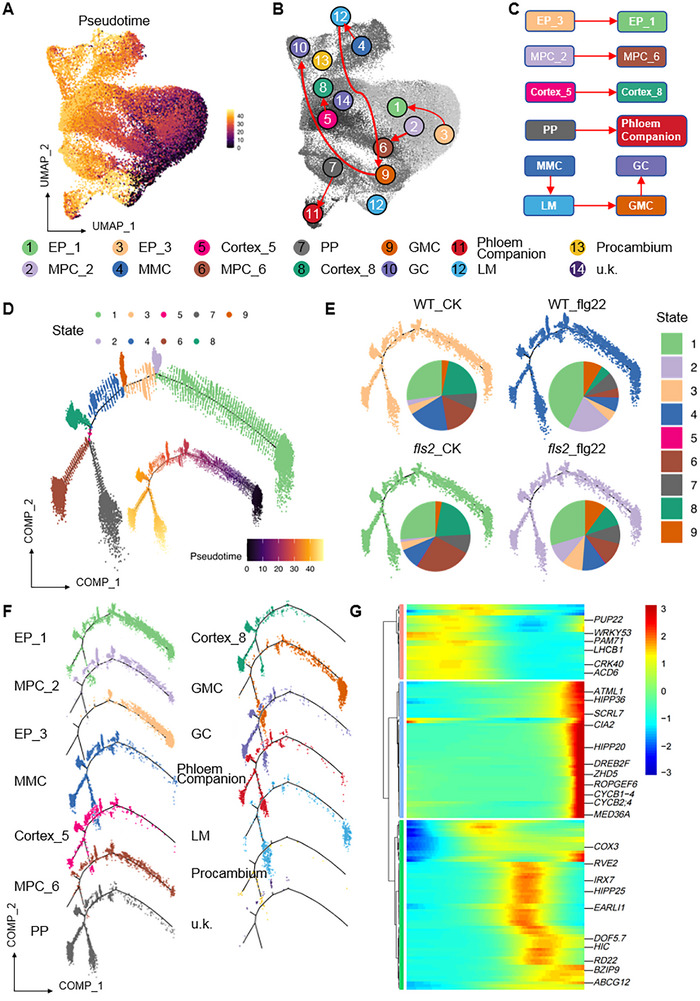
Pseudotime analysis of differentiation trajectories across distinct cell types. (A) Pseudotime analysis of cells from all four samples, visualized on a UMAP projection. The color bar indicates the developmental trajectory of cells along pseudotime, with darker to lighter hues representing progression from early to late stages. (B,C) Simulation of the developmental sequence of different cell clusters along the trajectory inferred in (A). (D) Pseudotime‐based classification of cells into nine distinct developmental states, ordered from early to late stages. Cells distributed in different states are distinguished by different colors. (E) Pseudotime trajectory analysis of individual samples; pie charts illustrate the proportion of cells in different states across various samples. (F) Distribution of cells from each cluster along the developmental trajectory, displayed separately. (G) Heatmap illustrating dynamic gene expression patterns across pseudotime stages, with representative genes highlighted on the right.

To elucidate the regulatory impact of flg22‐triggered immune responses on developmental dynamics in *Arabidopsis* cotyledon cells, we performed a systematic pseudotime analysis of cell cluster variations across four sample groups: WT_CK, WT_flg22, *fls2*_CK, and *fls2*_flg22. The pseudotime analysis delineated the developmental trajectories of distinct cell lineages (Figure 2; Table S6). Stomatal lineage cells progressed along the differentiation path from MMCs to LMs, GMCs, and finally GCs. Vascular cells developed from protophloem cells into Phloem Companion cells, while epidermal cells transitioned from EP_3 to EP_1. Mesophyll cells exhibited a developmental dynamic from MPC_2 to MPC_6 (Figure 2A–C). Notably, EP_3 and MPC_2 were predominantly located at the early stage of the pseudotime trajectory, supporting their identity as flg22‐responsive cell types with potentially delayed development (Figure 2D–F).

### 2.4 Gene Expression Profiles and GO Functional Analysis of Different Cell Types

Through comparative GO functional enrichment analysis of differentially expressed genes (DEGs) among cell clusters across samples (Table S7), this study systematically analyzed the transcriptional regulatory characteristics of cotyledon cell types responding to immune stimulation. In the WT_flg22 versus WT_CK comparison, heatmap results revealed significant differences in DEG quantities among clusters (Figure S6A), indicating cell‐type‐specific responses to flg22. Notably, the GO term “response to hypoxia” was significantly enriched across all clusters (Figure S6B). Among them, MPC_2, PP, GC, and MMC clusters exhibited similar functional characteristics in upregulated genes, while the Phloem Companion cluster (Cluster 11) showed upregulated genes primarily enriched in precursor metabolite generation, energy production, and photosynthesis. Immune stress‐related GO terms, including “chitin response” and “oxidative stress response,” were significantly enriched in EP_1, EP_3, MMC, PP, and Procambium clusters but displayed lower enrichment in MPC_5. Conversely, downregulated genes in WT_flg22 were predominantly associated with “defense response to fungus,” “indole‐containing compound metabolic process,” and “proteasome” functions (Figure S6C).

In the fls2_flg22 versus fls2_CK comparison, most clusters (except EP_3, Procambium, and unclassified Cluster 14) contained fewer than 100 DEGs, predominantly upregulated (Figure S7A). GO analysis indicated these upregulated genes were mainly involved in “defense response to fungus,” “photosynthesis,” and “thylakoid” functions (Figure S7B), suggesting that despite impaired flg22 perception, the *fls2* mutant may activate some immune‐related genes through alternative pathways.

The fls2_CK versus WT_CK comparison revealed limited transcriptomic differences under basal conditions. Most clusters (excluding EP_3, PROCAMBIUM, and Cluster 14) contained few DEGs, primarily downregulated (Figure S8A). Upregulated genes were enriched in photosynthesis‐related pathways (Figure S8B), while downregulated genes were associated with immune system function and oxidative stress response (Figure S8C). Similarly, in the fls2_flg22 versus WT_flg22 comparison, seven cell types exhibited fewer than 100 DEGs, mostly downregulated (Figure S9A). Upregulated genes primarily participated in photosynthesis‐related functions (Figure S9B), whereas downregulated genes were enriched in immune response pathways (Figure S9C), likely reflecting impaired induction of immune‐related genes in *fls2* mutants upon flg22 treatment.

Strikingly, the EP_3 cluster displayed significant DEG changes across all comparisons. In WT_flg22 versus WT_CK, EP_3 upregulated genes were enriched in immune stress responses, while downregulated genes participated in growth‐related metabolism. Conversely, in all three *fls2*‐involving comparisons, EP_3 downregulated genes were linked to immune responses, while upregulated genes were enriched in photosynthesis‐related functions (Figures S6–S9s). These results demonstrate that EP_3 immune functionality strictly depends on FLS2‐mediated signaling. In contrast, the MPC_2 cluster showed fewer DEGs across comparisons, with differential genes mainly enriched in photosynthesis. Notably, flg22 induced immune response‐related gene upregulation in MPC_2 only in WT samples, while photosynthesis‐related gene downregulation occurred in *fls2* mutants. Collectively, these data indicate MPC_2 specifically responds to FLS2‐dependent immune signals while maintaining photosynthetic functionality.

### 2.5 Analysis of the Expression Pattern of FLS2 Signaling Associated Genes

To investigate the heterogeneity of immune responses between *fls2* mutants and WT plants, we systematically analyzed the expression profiles and biological functions of FLS2‐dependent signaling pathways and other immune‐related genes in the four sample groups (WT_CK, WT_flg22, fls2_CK, and fls2_flg22). For marker gene selection, we focused on two major categories: (1) Cysteine‐rich receptor‐like kinases (CRKs), which belong to the DUF‐26‐containing RLK family and play crucial regulatory roles in plant immunity, abiotic stress responses, and growth development [49]; (2) Core components of the FLS2 immune signaling pathway, including pattern recognition receptors [*FLS2*, *BAK1*, *BOTRYTIS‐INDUCED KINASE1* (*BIK1*)], ROS metabolic regulators [(*RESPIRATORY BURST OXIDASE HOMOLOGUE D* (*RBOHD*)], MAPK cascade elements (*MPK3*, *MPK11*), and negative regulators [*PLANT U‐BOX 23* (PUB23), *NDR1/HIN1‐LIKE* (*NHL10*)].

Expression analysis revealed that flg22 treatment significantly upregulated most immune marker genes in WT_flg22 compared to WT_CK (Figure S10A). Notably, this induction was completely abolished in the *fls2* mutant, with no significant differences observed between *fls2_flg22* and *fls2_CK*. Intriguingly, besides FLS2‐dependent signaling, other immune‐related genes (e.g., *LYSM‐CONTAINING RECEPTOR‐LIKE KINASE 5* (*LYK5*), *CRK14*, *CRK31*, and *CALCIUM‐DEPENDENT PROTEIN KINASE 11* (*CPK11*) were also markedly upregulated in WT_flg22, while showing slight downregulation in *fls2_flg22* compared to *fls2_CK* (Figure S10A).

Single‐cell expression profiling demonstrated that upstream pathway components (*FLS2*, *BAK1*, *MPK3*, *MPK11*) were expressed in relatively rare cell populations (<5% of total cells), whereas downstream effectors (*PUB23*, *RBOHD*, *NHL10*) exhibited broader expression patterns (>10% of cells) (Figure S10B). This distribution pattern suggests that FLS2‐mediated immune signals may initiate in specific cell types before propagating to broader cellular populations, thereby amplifying the immune response.

GO enrichment analysis showed that the DEGs in upstream component‐expressing cells *(FLS2*, *BAK1*, *BIK1*, *MPK3*) were significantly enriched for “response to oxidative stress” and “cellular response to oxygen levels” (Figure S10C; Table S8). Downstream component‐expressing cells (*RBOHD*, *PUB23*, *MPK11*) displayed highly similar enrichment patterns. Notably, *NHL10*‐expressing cells—as terminal pathway effectors [50]—exhibited distinct GO enrichment profiles, potentially representing the final response state of the FLS2 signaling cascade (Figure S10C).

Pseudotime analysis further identified EP_3 and MPC_2 as key cell populations showing specific high expression of immune markers, particularly downstream components *MPK3*, *MPK11*, and *PUB23* (Figure S10D). These findings strongly suggest that EP_3 and MPC_2 cells play pivotal roles in mediating flg22‐induced immune responses.

### 2.6 Transcription Factor Analysis in PCs and MPCs

**FIGURE 3:**
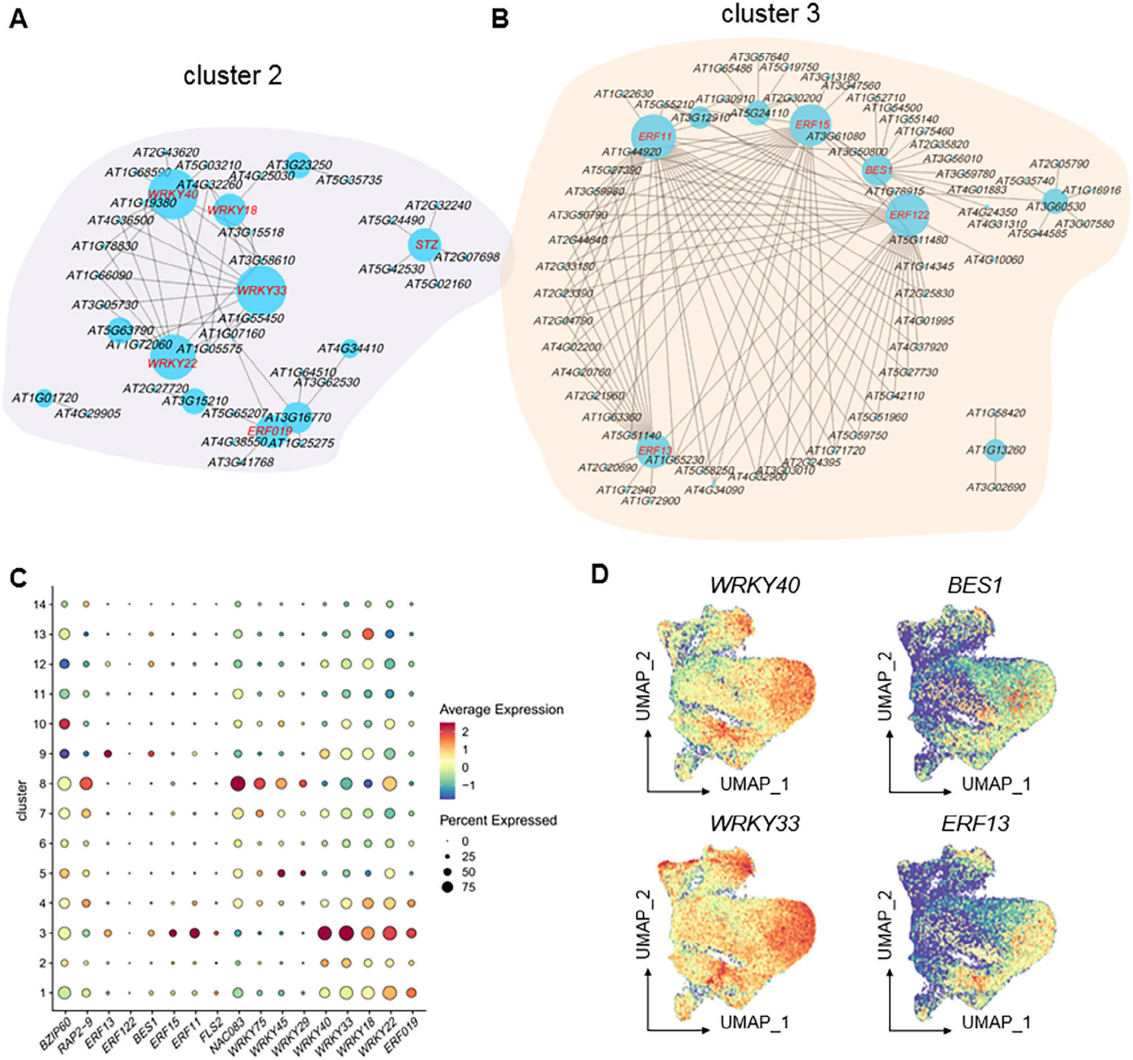
Identification of transcription factor regulatory networks in immune‐responsive cells MPC_2 and EP_3. (A) Regulatory network analysis of transcription factors (TFs) in MPC_2. (B) Regulatory network analysis of TFs in EP_3. (C) Dot plot displaying expression levels of representative TFs across different cell clusters. (D) Feature plot illustrating expression patterns of representative TFs.

To elucidate the regulatory mechanisms of the FLS2 signaling pathway, we systematically analyzed the transcription factor (TF) regulatory networks in MPC_2 and EP_3 cell clusters (Figure 3). Through screening of core transcription factors based on regulatory index (number of target genes), we identified that the core transcription factors in the MPC_2 cluster mainly included WRKY family members *WRKY40* (*AT1G80840*), *WRKY18* (*AT4G31800*), *WRKY33* (*AT2G38470*), *WRKY22* (*AT4G01250*), as well as *ERF19* (*AT1G22810*) and the salt‐tolerance zinc finger protein *SALT TOLERANCE ZINC FINGER* (*STZ*) (*AT1G27730*) (Figure 3A,C). Previous studies have demonstrated that transcription factors such as WRKY33, WRKY40, and WRKY18 play crucial roles in regulating plant immune responses and ROS homeostasis [51]. Therefore, the high expression of these WRKY transcription factors in the MPC_2 cluster may be closely associated with their involvement in flg22‐induced immune responses.

In the EP_3 cluster, the identified core transcription factors were primarily related to the ethylene signaling pathway, including *ERF11* (*AT1G28370*), *ERF15* (*AT2G31230*), *BES1* (*BRI1‐EMS‐SUPPRESSOR 1*, *AT1G19350*), *ERF112* (*AT2G33710*), and *ERF13* (*AT2G44840*) (Figure 3B,D). As an important regulator of PTI, ethylene can regulate FLS2 transcriptional expression through direct binding to EIN3/EIL transcription factors that depend on EIN2 activity accumulation [52]. Furthermore, ethylene can modulate pattern‐triggered immune responses through interactions with other plant hormones such as SA and JA [53], all of which function downstream of the FLS2 signaling pathway [54]. Therefore, the high expression of ethylene signaling component‐related transcription factors in EP_3 cells may be a key factor in this cell type's specific response to flg22 treatment, while these ERF family transcription factors may also play important regulatory roles in the growth and developmental processes of immune‐related cells.

### 2.7 Flg22 Stress Treatment Affects the Development of PCs and MPCs

Next, we systematically evaluated the roles of PCs and MPCs in flg22‐induced immune responses using a multi‐scale analytical approach (Figure S11). Semi‐thin section observations revealed significant morphological alterations in WT seedling cotyledons following flg22 treatment: both epidermal and mesophyll cells exhibited reduced cell volumes with more compact arrangements, accompanied by markedly decreased intercellular spaces in spongy tissues (Figure S11A). In contrast, *fls2* mutant cotyledons showed no significant cellular morphological changes (Figure S11A). These results confirm that flg22 significantly inhibits WT cotyledon growth through the FLS2 receptor‐mediated signaling pathway, while exerting minimal effects on *fls2* mutants. Confocal microscopy quantitative analysis provided more detailed cellular phenotypic characteristics: compared to WT_CK, WT_flg22 samples exhibited significant epidermal developmental alterations, including increased proportions of small‐sized meristemoid mother cells and protodermal cells, as well as shortened serrated projections in pavement cells (Figure S11B). Concurrently, mesophyll cells displayed reduced volumes and increased packing density, leading to significantly elevated areal densities of both MPCs and PCs (Figure S11B–D). Notably, fls2_flg22 showed no significant differences in these cellular morphological parameters compared to fls2_CK, including proportions of small epidermal cells, pavement cell morphological features, or mesophyll cell arrangement patterns. Flow cytometry further validated these findings, demonstrating significantly reduced average cell volumes in WT cotyledons after flg22 treatment, whereas *fls2* cotyledons showed negligible changes (Figure S11E,F).

### 2.8 Hypoxic Signaling Collaborates With flg22 Immune Responses to Regulate Seedling Development

scRNA‐seq analysis revealed significant enrichment of “cellular response to hypoxia” genes across cell types following flg22 treatment (Figure S6C), suggesting widespread hypoxic stress accompanies flg22 immune responses. Given hypoxia's established role in inhibiting cellular growth and development, we hypothesized that flg22‐mediated seedling growth inhibition involves induced hypoxic stress, with hypoxic signaling pathways participating in immune response regulation.

To test this, we examined interactions between hypoxic signaling and flg22 responses. Key components of the O_2_‐dependent N‐end rule pathway [arginyl‐tRNA:protein transferase 1 (*ATE1)*, PROTEOLYSIS 6 (*PRT6)*, LITTLE ZIPPER 2 (*ZPR2)*, PLANT CYSTEINE OXIDASE 1 (*PCO1)* and PCO2] regulate hypoxic stress perception and shoot apical meristem development [55, 56, 57]. Analysis of the expression patterns of the genes *ATE1*, *PRT6*, *PCO1*, and *PCO2* indicates that in WT_CK, these genes exhibit relatively high expression levels in stomatal cells (GMC, LM, GC) and procambium, while showing lower expression levels in EP_3. However, in WT_flg22 following flg22 treatment, the expression levels of these genes in EP_3 increase (Figure S12). To investigate the effects of hypoxia stress and related signaling pathways on the expression of key hypoxia‐responsive genes in *Arabidopsis* seedlings, we analyzed the expression levels of five genes: *ATE1*, *ZPR2*, *PRT6*, *PCO1*, and *PCO2* under flg22 treatment, hypoxia treatment, and combined hypoxia+flg22 treatment, using untreated samples as the control. qPCR results showed that after flg22 treatment, the expression levels of *ZPR2*, *PCO1*, and *PCO2* were significantly up‐regulated in the cotyledons of wild‐type seedlings, while the expression of *ATE1* and *PRT6* was significantly down‐regulated (Figure S13A). Under hypoxia treatment alone, the expression trends of these genes were consistent with those observed under flg22 treatment, but the changes were more pronounced (Figure S13A). Furthermore, under the combined hypoxia and flg22 treatment, the expression trends of all five genes remained consistent with the flg22 treatment, and the changes were the most significant (Figure S13A). These results suggest that hypoxia may synergistically enhance the transcriptional regulation mediated by flg22.

Confocal microscopy of an *FLS2pro:FLS2‐GFP* reporter further revealed dynamic subcellular relocalization: under control conditions, FLS2‐GFP localized primarily to the plasma membrane; flg22 treatment reduced membrane continuity with emergence of intracellular puncta; hypoxia alone induced similar internalization; while combined hypoxia+flg22 exacerbated this trafficking (Figure S13B), aligning with known flg22‐induced FLS2 dynamics [58].

Concurrently, all treatments significantly reduced FLS2 transcript levels (Figure S13C). Notably, *fls2* mutants exhibited enhanced hypoxia tolerance versus WT. Together, these results indicate FLS2 participates in hypoxia responses, while hypoxia signaling potentiates flg22‐mediated growth inhibition by modulating FLS2 expression and subcellular trafficking.

### 2.9 Hypoxic Signaling Mutants Exhibit Attenuated Responses to flg22‐Induced Immune Stress

**FIGURE 4:**
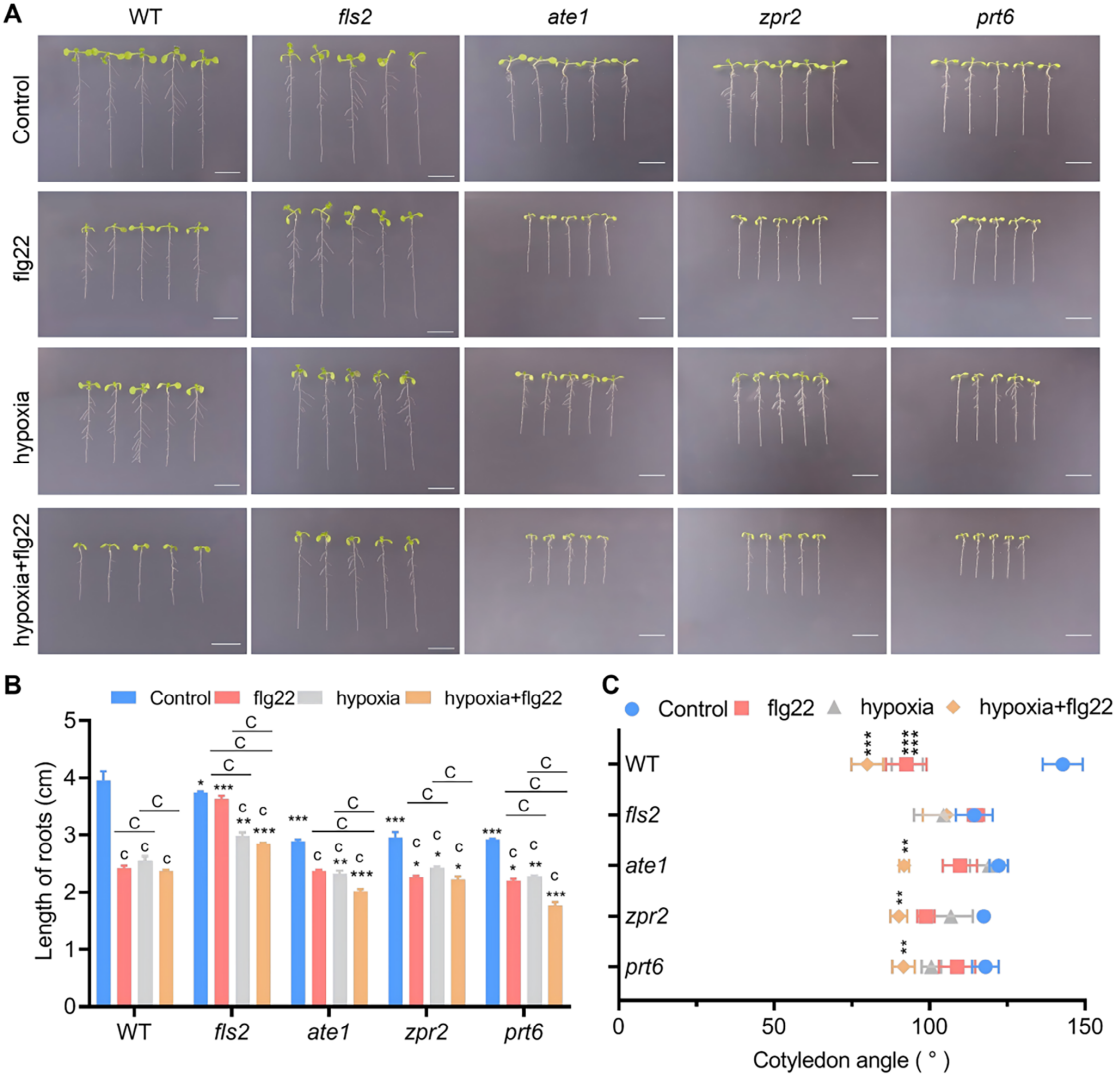
Effects of flg22 and hypoxia treatments on WT, *fls2*, *ate1*,  *zpr2* and *prt6* seedlings. Phenotypic analysis of 3‐day‐old WT, *fls2*, *ate1*, *zpr2* and *prt6*  seedlings after 3 days of treatment under control conditions, flg22 treatment, hypoxia, or combined hypoxia+flg22 treatment. (A) Representative seedling phenotypes of 3‐day‐old WT, *fls2*, *ate1*,  *zpr2* and * prt6* seedlings. Scale bar = 1 cm. (B) Quantitative analysis of root length of seedlings in (A). Data represent mean ± SD (n = 3). Statistical significance was determined by two‐way ANOVA followed by Tukey's test. Significant differences between mutants and WT are indicated by asterisks (*p < 0.05, ***p < 0.001). Significant differences between treatment groups and controls are denoted by lowercase letters (*cp* < 0.001). Significant differences among treatment groups are marked by uppercase letters (*Cp* < 0.001). (C) Measurement of cotyledon opening angle. Data are presented as mean ± SD (*n* = 3). Significant differences between treatment and control within the same genetype were determined by two‐way ANOVA followed by Tukey's test and are denoted by asterisks: **p < 0.01, ***p < 0.001.

Using hypoxic signaling mutants *ate1*, *prt6*, and *zpr2*, we performed flg22 treatment and hypoxic stress assays (Figure 4). Phenotypic analysis revealed that hypoxia induced significant root shortening and reduced cotyledon angles in both WT and *fls2* seedlings, phenocopying flg22‐treated WT (Figure 4A–C). *ate1*, *zpr2*, and *prt6* mutants exhibited similar but significantly attenuated changes compared to WT and *fls2* (Figure 4A–C). Combined hypoxia+flg22 treatment caused the most severe growth inhibition across all genotypes. Under this condition, *fls2* seedlings showed minimal sensitivity, while the hypoxic mutants displayed intermediate responses between *fls2* and WT (Figure 4A–C).

Cotyledon morphology and semi‐thin section analysis showed hypoxia reduced leaf area in WT and *fls2*, with smaller cell volumes, tighter packing, and diminished spongy mesophyll intercellular spaces—mirroring but less severe than flg22‐treated WT phenotypes (Figure S14). Hypoxic mutants exhibited similar but weaker alterations (Figure S14). Combined treatment induced the most dramatic cellular changes, with *fls2 *least affected and mutants showing intermediate sensitivity (Figure S14).

To delineate the patterns of ROS production across different genetic backgrounds, we employed both NBT/DAB histochemical staining and the 2’,7’‐dichlorodihydrofluorescein diacetate (H2DCFDA) fluorescent probe assay. The results from these complementary methodologies were concordant, collectively revealing the genetic interactions between flg22 and hypoxia signaling in orchestrating ROS bursts. Histochemical staining demonstrated that flg22 treatment triggered a substantial ROS accumulation in WT plants (Figures S15A,B and S16A,B). This response was entirely abolished in the *fls2* mutant, establishing FLS2 as an essential component for flg22 perception and downstream ROS activation. In contrast, other tested mutants, including *ate1*, *prt6*, and *zpr2*, retained significant ROS responsiveness to flg22 (Figures S15A,B and S16A,B). Under hypoxic stress, both WT and *fls2* exhibited a moderate yet significant increase in ROS, whereas the other mutants showed no significant response (Figures S15A,B and S16A,B). Notably, the combined application of flg22 and hypoxia elicited the most intense ROS burst across all genotypes (Figures S15A,B and S16A,B). WT plants were the most sensitive to this combined treatment, while the *fls2* mutant displayed the lowest level of accumulation, indicating its strongest resistance (Figures S15A,B and S16A,B). Quantitative assessment of intracellular ROS using H_2_DCFDA further validated and refined these findings. flg22 treatment induced an approximately threefold increase in ROS levels in WT, but the *fls2* mutant remained completely insensitive, reaffirming the central role of FLS2 (Figure S17A,B). All other mutants exhibited varying degrees of flg22‐induced ROS production. Hypoxia treatment significantly elevated ROS levels in all genotypes, with the most pronounced response observed in WT (Figure S17A,B). The combined treatment resulted in the most robust ROS accumulation across all genotypes, displaying a synergistic effect. Two independent detection methods—H_2_DCFDA reporting on general intracellular ROS levels and DAB staining being more indicative of hydrogen peroxide‐associated oxidative stress—converged to confirm distinct functional roles for the respective mutants within the flg22 and hypoxia signaling pathways. Our results firmly establish the indispensable role of FLS2 in the flg22‐triggered ROS burst and uncover the existence of an FLS2‐independent, yet synergistic, backup mechanism that enables potent ROS activation under concomitant hypoxia stress.

PC and MPC analysis confirmed hypoxia increased small epidermal cell proportions, shortened PC lobes, reduced MPC volumes, and elevated packing density in WT and *fls2*, with hypoxic mutants showing attenuated effects. Combined treatment caused the most severe cellular alterations, consistent with the phenotypic hierarchy (Figures S18A,B and S19A,B).

Notably, flg22 alone significantly inhibited growth in hypoxic mutants, causing root shortening, reduced cotyledon angles/areas/cell volumes, increased small epidermal cells, and elevated MPC/PC densities. However, all changes were consistently weaker than in WT (Figure 4; Figure S14).

Collectively, hypoxic signaling mutants exhibit reduced sensitivity to flg22, indicating impaired hypoxia perception attenuates flg22‐mediated growth inhibition. This confirms hypoxic signaling components ATE1, ZPR2, and PRT6 participate in flg22‐induced immune responses and regulate immune‐mediated growth. Hypoxia alone caused weaker inhibition than flg22 in WT, while combined treatment showed additive effects, suggesting flg22‐mediated growth suppression operates partially through concomitant hypoxic responses.

### 2.10 FLS2‐Mediated Immune Signaling Regulates Plant Defense against Pseudomonas Syringae

*Pseudomonas syringae pv. maculicola* (*Psm*) carrying the effector AvrRpt2 (*Psm AvrRpt2*) induces non‐autophagic cell death in *Arabidopsis* via activation of the RPS2 protein [59], making it a valuable tool for hypersensitive response studies [60]. *Psm AvrRpt2* infection triggers ETI, while *Psm* alone primarily induces pathogen‐associated molecular PTI [61]. Given our previous findings implicating hypoxic signaling in FLS2‐mediated immune regulation, we employed *Psm* and *Psm AvrRpt2* infection assays to investigate hypoxic signaling's role in broader immune responses and PTI‐ETI interplay.

**FIGURE 5:**
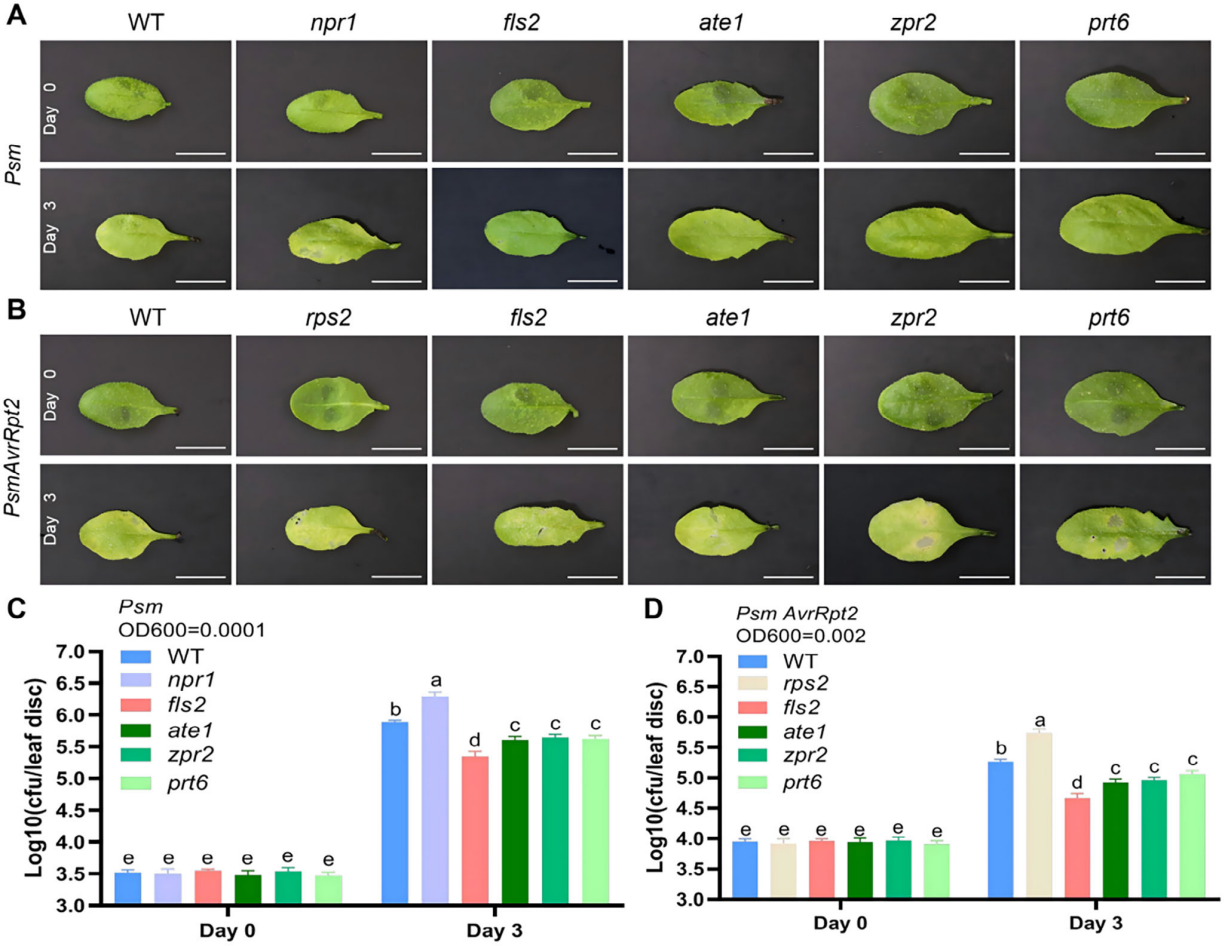
Analysis of the immune responses of WT, *npr1*, *fls2*, *ate1*, *zpr2*, and *prt6* seedlings to *Pseudomonas syringae* infection. Pathogenicity analysis of three‐week‐old WT, *npr1*, *fls2*, *ate1*, *zpr2*, and *prt6* seedlings inoculated with *Psm* (OD600 = 0.0001) or *Psm/AvrRpt2* (OD600 = 0.002) at day 0 (1 h post‐inoculation) and day 3. (A) Phenotypic symptoms of *Psm*‐infected leaves of WT, *npr1*, *fls2*, *ate1*, *zpr2*, and *prt6* seedlings. Scale bar = 1 cm. (B) Phenotypic symptoms of *Psm/AvrRpt2*‐infected leaves of WT, *npr1*, *fls2*, *ate1*, *zpr2*, and *prt6* seedlings. Scale bar = 1 cm. (C) Bacterial biomass quantification (Colony‐Forming Units, CFU) in *Psm*‐infected leaves of WT, *npr1*, *fls2*, *ate1*, *zpr2*, and *prt6* seedlings. Data represent mean ± SD (n = 3). Statistical significance was determined by one‐way ANOVA followed by Tukey's test (p < 0.05), with different letters indicating significant differences between groups. (D) Bacterial biomass quantification (CFU) in *Psm/AvrRpt2*‐infected leaves of WT, *npr1*, *fls2*, *ate1*, *zpr2*, and *prt6* seedlings. Data represent mean ± SD (n = 3). Statistical significance was determined by one‐way ANOVA followed by Tukey's test (p < 0.05), with different letters indicating significant differences between groups.

Three days post‐inoculation (dpi) with *Psm*, *npr1 *mutants exhibited the most severe symptoms, including complete leaf chlorosis with necrotic spots (Figure 5A). In contrast, *fls2*, *ate1*, *zpr2*, and *prt6* mutants displayed localized chlorosis, with severity intermediate between *npr1* and WT (Figure 5A). Pathogen biomass quantification revealed no significant differences at initial inoculation (0 dpi; Figure 5C). By 3 dpi, *Psm* biomass increased significantly across all genotypes, with *npr1* showing the highest levels, followed by* fls2*, *ate1*, *zpr2*, and *prt6*, while WT maintained the higher load than *fls2*, *ate1*, *zpr2*, and *prt6, but lower than npr1*(Figure 5C). Following *Psm AvrRpt2* infection (3 dpi), *rps2* mutants showed mild chlorosis, while WT leaves displayed near‐complete chlorosis and wilting (Figure 5B). Notably, *fls2*, *ate1*, *zpr2*, and *prt6* mutants exhibited complete chlorosis accompanied by distinct necrotic lesions (Figure 5B). Initial pathogen loads were similar across genotypes (Figure 5D). By 3 dpi,* rps2* accumulated the highest biomass, followed by WT, while *fls2*, *ate1*, and* zpr2* mutants harbored significantly lower levels than WT (Figure 5D). Collectively, these results indicate that the loss of function of FLS2 and ATE1 leads to a rewiring of plant immune homeostasis. Although FLS2 is crucial for canonical PTI signaling, its absence unexpectedly derepresses the AvrRpt2‐RPS2‐mediated ETI pathway, leading to enhanced pathogen restriction. This enhanced resistance is observed during *Psm AvrRpt2* infection, demonstrating that a functional PTI pathway can exert a homeostatic constraint on NLR‐mediated immunity. Disruption of this constraint, therefore, potentiates ETI. This enhanced resistance is also observed in *Psm* infections, suggesting the activation of a potent compensatory mechanism independent of the canonical FLS2‐PTI pathway.

### 2.11 ERF13 Acts as a Key Regulator in *Arabidopsis* flg22 Immune Responses

Analysis of core transcription factors in the EP_3 cluster highlighted crucial roles for ERF family members *ERF11*, *ERF15*, *ERF112*, and *ERF13* (Figure 3B). While *ERF15* [62, 63], *ERF11* [64, 65], and* ERF112* [66] have established roles in immune stress responses, and *ERF13* functions in auxin signaling during lateral root development [67], its involvement in FLS2‐mediated immunity was unclear. The qPCR results delineate distinct expression patterns among the ERF transcription factors in response to flg22. In WT seedlings, flg22 treatment significantly up‐regulated *ERF13* and *ERF112* expression, while *ERF15* showed a moderate increase and* ERF11* was unaffected. Notably, this response was abrogated in the* fls2 *mutant; instead of induction, *ERF13* expression was significantly repressed, and the transcript levels of *ERF11*,* ERF15*, and* ERF112* remained unchanged. This genetic evidence underscores a critical role for ERF13 downstream of the FLS2 receptor in mediating flg22‐triggered signaling (Figure S20A). Furthermore, *ERF13* transcript levels were significantly suppressed by hypoxia. This suppression was even more pronounced under the combined hypoxia+flg22 stress, demonstrating a synergistic downregulation (Figure S20B).

**FIGURE 6:**
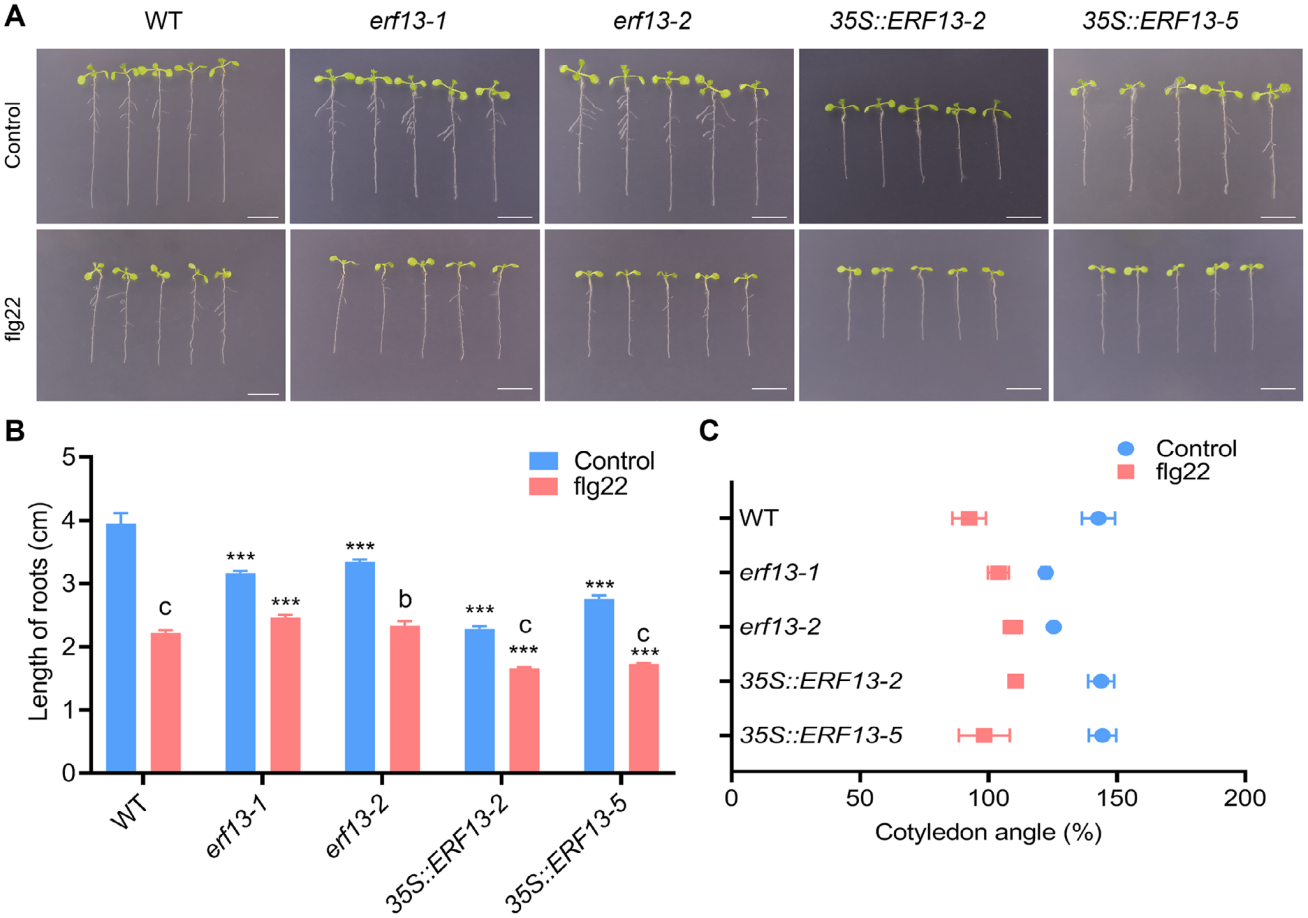
Effects of flg22 treatment on WT, *erf13‐1*, *erf13‐2*, *35S::ERF13‐2*, and* 35S::ERF13‐5* seedlings. Phenotypic analysis of 3‐day‐old WT, *erf13‐1*, *erf13‐2*, *35S::ERF13‐2*, and* 35S::ERF13‐5* seedlings following 3 days of control or flg22 treatment. (A) Representative seedling phenotypes. Scale bar = 1 cm. (B) Quantitative measurement of root length. Data represent mean ± SD (*n* = 3). Statistical significance was determined by two‐way ANOVA followed by Tukey's test. Significant differences between mutants and WT are indicated by asterisks (***p < 0.001). Significant differences between treatment groups and controls are denoted by lowercase letters (*bp* < 0.01, *cp* < 0.001). (C) Analysis of cotyledon opening angle. Data are presented as mean ± SD (*n* = 3). Significant differences between treatment and control within the same genetype were determined by two‐way ANOVA followed by Tukey's test and are denoted by asterisks: **p < 0.01, ***p < 0.001.

To define *ERF13*’s role in flg22 responses, we analyzed loss‐of‐function mutants (*erf13‐1*, *erf13‐2*) and overexpression lines (*35S::ERF13‐2*, *35S::ERF13‐5*). All genotypes showed flg22‐induced growth inhibition. However, *erf13‐1* and *erf13‐2* exhibited milder phenotypes than WT: reduced root shortening, smaller decreases in cotyledon angles/areas, and attenuated cell volume reduction (Figure 6, A–C; Figures S21 and S22). Conversely, overexpression lines displayed similar or more severe inhibition than WT (Figure 6; Figures S21 and S22).

MPC and PC density increased in all *erf13* genotypes after flg22 treatment, manifesting as smaller epidermal cells and tightly packed, reduced‐volume mesophyll cells (Figures S18C,D and S19C,D). Nevertheless, these cellular changes were consistently weaker in mutants than in WT, particularly in loss‐of‐function lines (Figures S18C,D and S19C,D), suggesting *ERF13* differentially regulates growth and cellular morphogenesis.

 *erf13* mutants exhibited significantly attenuated growth inhibition and weaker MPC/PC proliferation under hypoxia and combined treatments compared to WT, while overexpression lines showed exacerbated effects (Figures S18C,D and S19C,D). This confirms *ERF13*’s dual role in mediating hypoxia signaling and flg22‐induced growth inhibition.

To define the genetic role of ERF13 in ROS signaling, we analyzed responses of *erf13* mutants and overexpression lines to flg22 and hypoxia using complementary assays. NBT and DAB staining consistently showed that *erf13* mutants were completely insensitive to flg22, producing no detectable superoxide or H_2_O_2_‐related oxidation, indicating ERF13 is essential for flg22‐induced ROS (Figures S15C,D and S16C,D). Mutants also displayed reduced sensitivity to hypoxia alone and markedly enhanced resistance to combined hypoxia+flg22 stress, with significantly lower ROS than WT (Figures S15C,D and S16C,D). H_2_DCFDA quantification confirmed these results: the flg22‐induced ROS burst was severely impaired in *erf13 *mutants, and responses to hypoxia and combined treatment were strongly attenuated (Figure S17C,D). In contrast, *ERF13* overexpression generally enhanced sensitivity, though effects varied. Together, these results establish ERF13 as a essential positive regulator of flg22‐triggered ROS burst and a modulator of ROS signaling under hypoxia and combined stress.

**FIGURE 7:**
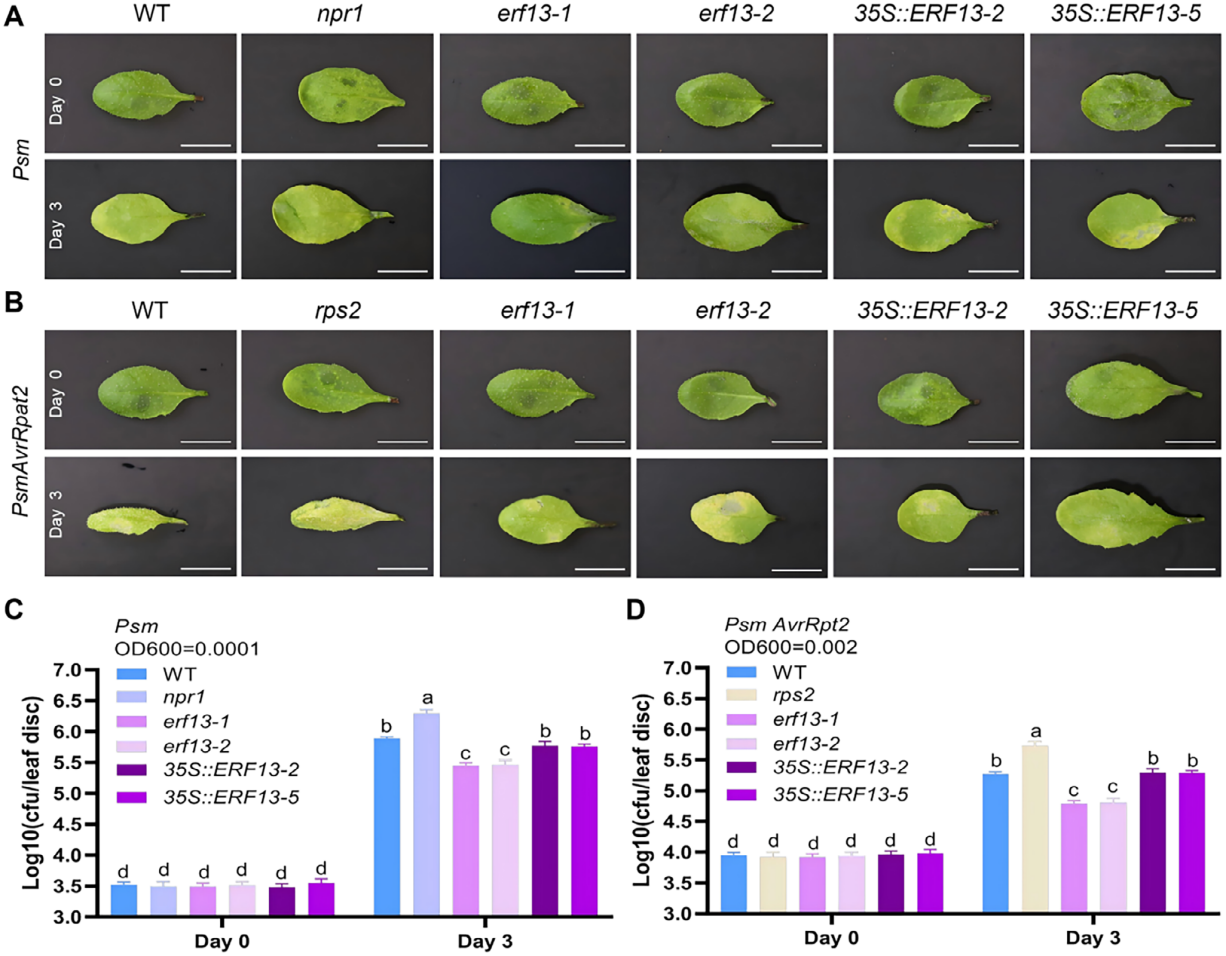
Analysis of the immune responses of WT, *npr1*, *erf13‐1*, *erf13‐2*, *35S::ERF13‐2*, and *35S::ERF13‐5* seedlings to *Pseudomonas syringae* infection. Pathogenicity analysis of three‐week‐old WT, *npr1*, *erf13‐1*, *erf13‐2*, *35S::ERF13‐2*, and *35S::ERF13‐5* seedlings inoculated with *Psm* (OD600 = 0.0001) or *Psm/AvrRpt2* (OD600 = 0.002) at day 0 (1 h post‐inoculation) and day 3. (A) Disease symptoms of *Psm*‐infected leaves of 3‐weeks‐old WT, *npr1*, *erf13‐1*, *erf13‐2*, *35S::ERF13‐2*, and *35S::ERF13‐5* seedlings. Scale bar = 1 cm. (B) Disease symptoms of *Psm/AvrRpt2*‐infected leaves of three‐week‐old WT, *npr1*, *erf13‐1*, *erf13‐2*, *35S::ERF13‐2*, and *35S::ERF13‐5* seedlings. Scale bar = 1 cm. (C) Bacterial growth quantification (CFU) in *Psm*‐infected leaves of three‐week‐old WT, *npr1*, *erf13‐1*, *erf13‐2*, *35S::ERF13‐2*, and *35S::ERF13‐5* seedlings. (D) Bacterial growth quantification (CFU) in *Psm/AvrRpt2*‐infected leaves of three‐week‐old WT, *npr1*, *erf13‐1*, *erf13‐2*, *35S::ERF13‐2*, and *35S::ERF13‐5* seedlings. Data represent mean ± SD (n = 3). Statistical significance was determined by one‐way ANOVA followed by Tukey's test (*p* < 0.05), with different letters indicating significant differences between groups.

Pathogen infection assays revealed compromised immunity in *erf13* mutants. Following *Psm* infection, *erf13* mutants developed more severe symptoms than WT or overexpression lines, with* npr1* showing the strongest susceptibility (Figure 7A). Pathogen quantification confirmed higher *Psm* biomass in* erf13* mutants than WT, while overexpression lines had lower levels (Figure 7C). For *Psm/AvrRpt2* (triggering ETI),* erf13* mutants displayed complete chlorosis and severe necrosis (Figure 7B), yet paradoxically harbored lower pathogen loads than WT (Figure 7D), potentially indicating *ERF13* deficiency hyperactivates ETI responses.

### 2.12 flg22‐Mediated Immune Signaling Regulates Cotyledon Development via Cell Cycle Modulation

Cell cycle control is essential for plant growth, development, and environmental responses. Pathogen infection can perturb cell cycle progression, and conversely, cell cycle dysregulation may trigger autoimmune responses [68]. To determine whether flg22‐induced immune signaling regulates *Arabidopsis* cotyledon development via the cell cycle, we analyzed cell cycle characteristics post‐immune response and assessed immune responses in cell cycle regulatory mutants (Figure S23).

Flow cytometry analysis of cotyledon ploidy revealed that flg22 treatment significantly decreased the proportion of 2C (diploid) cells while increasing higher ploidy cells (4C, 8C, 16C) in WT seedlings (Figure S23A,B). Untreated *fls2*, *ate1*, and *erf13‐1* mutants exhibited lower basal 2C proportions and higher 4C proportions than WT. Post‐flg22 treatment, *fls2* and *ate1* mutants showed reduced 2C proportions, but this decrease was significantly less pronounced than in WT. Conversely, *erf13‐1* mutants displayed a significant increase in 2C cells and a *decrease* in 4C, 8C, and 16C cells (Figure S23A,B).

Expression analysis of cell cycle negative regulators showed flg22 significantly downregulated *SIAMESE* (*SIM*), *SIAMESE‐RELATED 1* (*SMR1*), *E2FB*, and *E2FC* in WT cotyledons (Figure S23C–F). In *fls2 *mutants, *SIM*, *SMR*, and *E2FC* (but not *E2FB*) decreased, albeit less markedly than in WT. *ate1* mutants showed significant downregulation of *SIM*, *SMR*, and *E2FB* (but not *E2FC*). While all four genes decreased in *erf13‐1*, the reduction was significantly weaker than in WT (Figure S23C–F).

Cyclin‐dependent kinases (CDKs) are key cell cycle regulators. Studies link ETI to the nuclear envelope protein CPR5, which interacts with SIM and SMR1 to inhibit CDK activity. This regulates retinoblastoma‐related protein 1 (RBR1) phosphorylation, impacting E2F transcription factor expression and cell cycle progression [61]. We therefore analyzed immune responses in *sim smr*, *e2fabc*, and* cpr5 *mutants (Figure S24).

Flg22 significantly inhibited growth in *e2fabc*, *sim smr*, and *cpr5* mutants (Figure S24). Compared to WT, *sim smr* and *e2fabc* seedlings exhibited more severe root shortening, accompanied by slightly greater increases in cotyledon angle, decreases in leaf area, and reductions in cotyledon cell size. In contrast, *cpr5* mutants showed milder changes than WT in these parameters (Figure S24).

Comprehensive ROS assays revealed distinct genetic requirements for flg22‐ and hypoxia‐triggered oxidative bursts. The *cpr5* mutant consistently exhibited a attenuated ROS response across all assays. It showed a significantly reduced response to flg22, confirming CPR5 as a key positive regulator of flg22‐induced ROS production (Figures S15E,F and S16E,F). Furthermore, *cpr5* displayed hyposensitivity to hypoxia and the most pronounced resistance to the combined hypoxia+flg22 stress, with the lowest ROS accumulation among all genotypes (Figures S15E,F and S16E,F). In contrast, the *e2fbc* mutant generally demonstrated heightened sensitivity. It mounted a hyper‐sensitive response to flg22 in both NBT and DAB staining, and exhibited the strongest ROS accumulation under the combined treatment in the H_2_CFDA assay, suggesting an enhanced synergistic effect (Figure S17E,F). The *sim smr* mutant displayed a context‐dependent phenotype. While its response to flg22 alone was comparable to WT and it was less sensitive to hypoxia alone (Figure S17E,F). This indicates a potential role for SIM and SMR in restraining excessive ROS accumulation specifically under combined stress conditions.

Further analysis showed increased MPC and PC densities in all mutants post‐flg22 treatment (Figures S18E,F and S19E,F). Epidermal cells became smaller, while mesophyll cells decreased in volume and packed more tightly. However, the increase in MPC and PC density was slightly greater in *sim smr* and *e2fabc* mutants than in WT, and weaker in *cpr5* (Figures S18E,F and S19E,F).

To investigate whether the hypoxia response affects cotyledon cell cycle progression, we first detected the expression levels of cell cycle regulatory genes (*SIM*, *SMR*, *E2FB*, and *E2FC*) under hypoxia treatment and hypoxia+flg22 combined treatment (Figure S25). The results showed that in WT cotyledons, hypoxia treatment caused a slight upregulation of these genes, whereas combined treatment led to significant downregulation (Figure S25A). Further chromosomal ploidy analysis revealed no significant change in the ploidy distribution of WT cotyledon cells after hypoxia treatment. However, the proportion of 4C cells significantly decreased in *fls2*, *ate1*, and *erf13‐1* mutants. Under combined treatment, the proportion of high‐ploidy cells significantly increased in WT, *fls2*, and *ate1* mutants, while the* erf13‐1 *mutant showed the opposite trend (Figure S25B).

**FIGURE 8:**
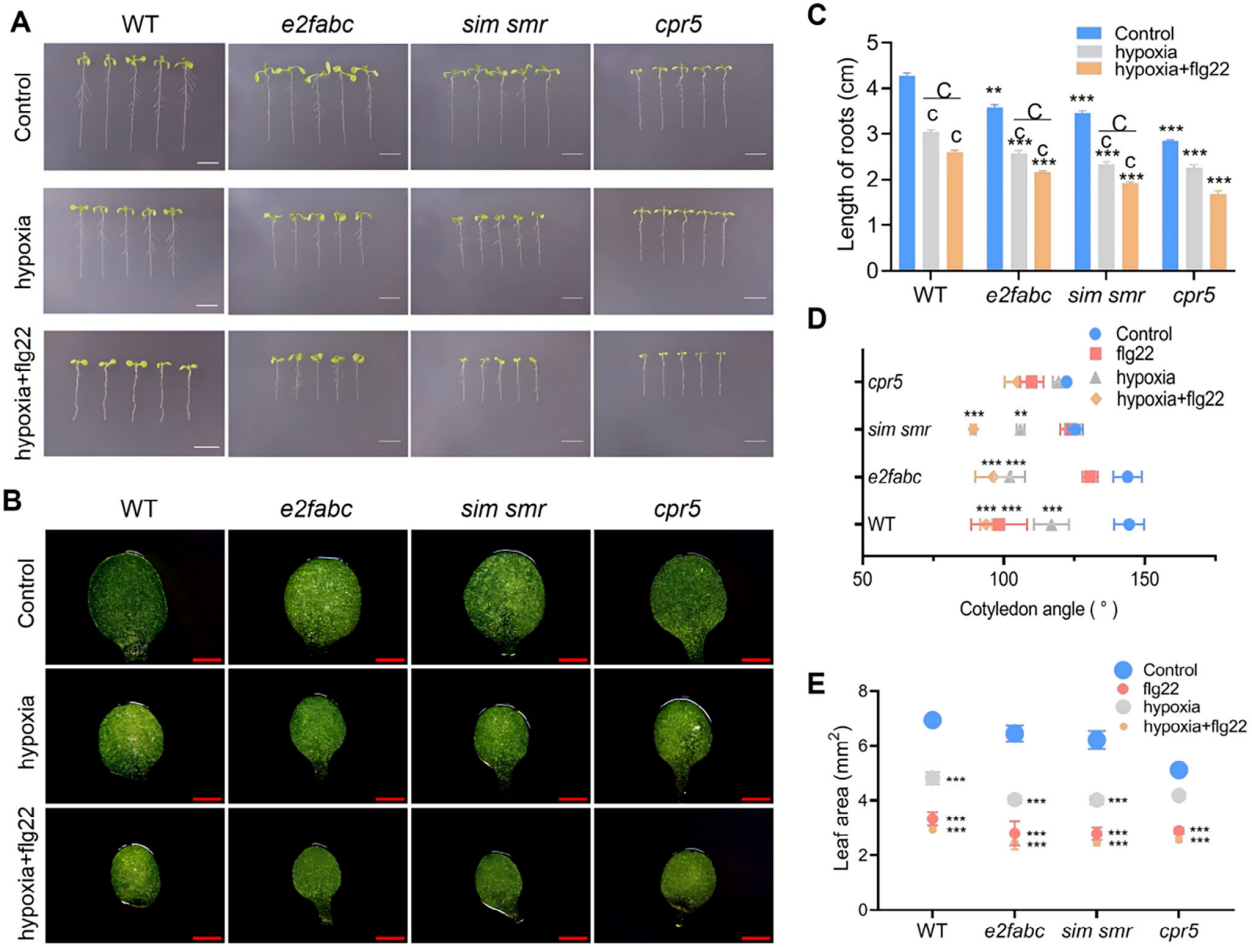
Effects of hypoxia and hypoxia + flg22 co‐treatment on seedlings of cell cycle regulatory mutants. Phenotypic analysis of 3‐day‐old WT, *e2fabc*, *sim smr*, and *cpr5 *seedlings following 3 days of control, hypoxia, or hypoxia + flg22 co‐treatment. (A) Representative whole‐seedling images of 3‐day‐old WT, *e2fabc*, *sim smr*, and *cpr5 *seedlings. Scale bar = 1 cm. (B) Representative cotyledon images of 3‐day‐old WT, *e2fabc*, *sim smr*, and *cpr5 *seedlings. Scale bar = 1 mm. (C) Root length quantification of seedlings in (A). Data are presented as mean ± SD (*n* = 3). Significant differences between mutants and WT within the same treatment were determined by two‐way ANOVA followed by Tukey's test and are denoted by asterisks: **p < 0.01, ***p < 0.001. Significant differences between treatment groups and their respective controls were determined by two‐way ANOVA followed by Tukey's test and are indicated by lowercase letters: *p* < 0.001. Significant differences among treatment groups are marked by uppercase letters (*Cp* < 0.001). (D) Cotyledon angle measurement of seedlings in (A). Data are presented as mean ± SD (*n* = 3). Significant differences between treatment and control within the same genetype were determined by two‐way ANOVA followed by Tukey's test and are denoted by asterisks: **p < 0.01, ***p < 0.001. (E) Cotyledon area quantification of 3‐day‐old WT, *e2fabc*, *sim smr*, and *cpr5 *seedlings. Data are presented as mean ± SD (*n* = 3). Significant differences between treatment and control within the same genetype were determined by two‐way ANOVA followed by Tukey's test and are denoted by asterisks: **p < 0.01, ***p < 0.001.

Cell cycle regulatory gene loss‐of‐function mutants (*e2fabc*, *sim smr*, *cpr5*) were further subjected to hypoxia and hypoxia+flg22 combined treatment. Statistical analysis of seedling root length, cotyledon angle, and cotyledon area showed that compared to WT, the growth phenotypes of *e2fabc* and *sim smr* were more strongly inhibited, while *cpr5* was less inhibited (Figure 8). Observation and statistics of PCs and MPCs showed that the increase in PCs and MPCs was smaller in *cpr5*, while it was more pronounced in *e2fabc* and *sim smr* (Figures S18E,F and S19E,F).

## 3 Discussion

### 3.1 Flg22‐Induced Immune Responses Modulate Cell Development and Growth in *Arabidopsis* Seedlings

Cell fate determination integrates intrinsic developmental signals and external environmental cues [45, 69]. Pathogen‐activated immune signals in plants trigger developmental and metabolic reprogramming, inducing phytohormones (e.g., SA, JA, ABA, ethylene, nitric oxide) and metabolites (e.g., ROS, glycosides, glutathione, phytoalexins) that directly regulate cell differentiation and seedling development [70, 71]. Consistent with this, flg22 treatment significantly inhibited WT seedling growth but not *fls2* mutants (Figure S1), confirming that flg22‐induced immune responses require FLS2 signaling to regulate cell differentiation and development, mediating growth inhibition.

Plant pathogen responses often initiate at the single‐cell level [70]. While bulk transcriptomics masks cell‐specific responses, single‐cell RNA sequencing (scRNA‐seq) enables precise cellular resolution. Given leaves harbor diverse microbes and cotyledons are critical for early development and stress defense [72], we performed scRNA‐seq on flg22‐treated WT and *fls2* mutant *Arabidopsis* cotyledons (Figure 1). Compared to control (WT_CK), WT_flg22 seedlings exhibited altered cell differentiation and development, whereas *fls2* mutants showed no significant changes before and after treatment (*fls2_CK* vs. *fls2_flg22*) (Figures 1 and 2). This further establishes the essential role of FLS2 in mediating flg22‐induced immune responses and developmental regulation.

### 3.2 Cell Type‐Specific Heterogeneity in flg22 Immune Responses

Although plants lack specialized immune cells, immune responses operate with significant cellular heterogeneity [73]. Different cell types employ distinct defense strategies against pathogens [74, 75]. Epidermal cells form the primary physical barrier (e.g., trichomes, cuticles, wax; [76]) and rapidly initiate PTI via plasma membrane‐localized receptors. Notably, epidermal mini‐chloroplasts compartmentalize immune components to regulate fungal invasion [74]. Mesophyll tissue facilitates photosynthesis and adaptation via intercellular air spaces [74].

Our scRNA‐seq revealed increased MPC_2 abundance in WT_flg22 versus WT_CK (Figure 1C,D). However, MPC_2 lacked immune gene enrichment (Figure S6), suggesting its role may involve photosynthetic/metabolic support for immunity. While flg22 altered proportions of some cell types (Figure 1C,D), pseudotime analysis showed conserved developmental trajectories: stomatal lineage (MMC→LM→GMC→GC), vascular (PP→Phloem Companion), EP_3→EP_1, and MPC_2→MPC_6 (Figure 2).

DEGs analysis identified upregulated biotic stress‐responsive genes regulating photosynthesis and amino acid‐derived metabolites. Immune genes were broadly upregulated in WT_flg22 across cell types (Figure S6). Sensitivity varied, with stomatal lineage (MMC, GC) and vascular cells (PP, Phloem Companion, Procambium) showing the most DEGs (Figure S6A). This aligns with their functions: stomatal closure blocks pathogen entry [77, 78], while phloem transports nutrients (attractive to pathogens) and signals, potentially negatively regulating flg22 responses via octopine synthase [79].

### 3.3 Epidermal and Mesophyll Cells Are Primary flg22 Responders in Cotyledons

scRNA‐seq studies highlight epidermal and guard cells as major responders to pathogens like *Colletotrichum higginsianum* [80] and *Pseudomonas syringae pv*. *tomato* DC3000 [81]. Similarly, our multidimensional analysis (cell proportions, pseudotime trajectories, DEGs, GO enrichment) identified EP_3 and MPC_2 as the most significantly altered populations in WT versus *fls2 *upon flg22 treatment (Figures 1 and 2; Figures S6–S9), suggesting they are primary flg22 targets. Root epidermal cells also show robust immune responses to flg22 and Pep1 [82]. Confocal microscopy and semi‐thin sectioning confirmed flg22‐mediated increases in cell density and tighter packing specifically in epidermal and mesophyll cells of WT (Figure S11), demonstrating growth inhibition in these key responder types.

### 3.4 flg22 Triggers Hypoxic Signaling to Suppress Growth

GO analysis revealed flg22 activated hypoxic signaling pathways across nearly all WT cell types (WT_flg22 vs. WT_CK; Figure S6). Hypoxia potently inhibits plant growth, suppressing development by impairing aerobic respiration [83]. Molecularly, hypoxia stabilizes ZPR2 via degradation regulated by ATE1 and PRT6, influencing shoot apical meristem activity [84, 85]. ATE1 also redundantly regulates leaf and stem development [85]. Flg22 inhibition of leaf and root development phenocopied hypoxia (11% O_2_; Figure 4; Figure S14). Coordinated downregulation of *ATE1 *and* PRT6* with* ZPR2* upregulation (Figure S13) mirrored hypoxia‐induced ZPR2 stabilization, suggesting flg22 induces the expression of hypoxia responses genes, potentially mediated by ATE1/ZPR2/PRT6.

Notably, our findings are supported by a recent independent study which reported that flg22‐induced transcriptional reprogramming encompasses a set of hypoxia‐responsive genes, and this overlap is conserved across *Brassicaceae* species [36]. Flg22 reduced FLS2 expression and triggered its relocalization from the plasma membrane, an effect replicated by hypoxia (Figure S13). While hypoxia inhibited *fls2 *mutant development (Figure 4), demonstrating FLS2 dispensability for flg22‐induced expression of hypoxia responses genes. Hypoxic signaling mutants exhibited greater flg22 resistance than WT under combined stress (Figure S14), suggesting hypoxic signaling acts downstream of FLS2 but is not absolutely essential for growth inhibition.

While our genetic and transcriptomic data strongly implicate components of the hypoxic signaling pathway (ATE1, PRT6, ZPR2) in modulating flg22 responses, this study primarily relies on transcriptional evidence and mutant phenotypes. We acknowledge that direct protein‐level validation—such as assessing ZPR2 protein stability, PCO enzyme activity, or accumulation of N‐degron substrates under flg22 treatment—would provide more conclusive mechanistic insights. Technical challenges in reliably detecting these specific proteins have currently precluded such analyses. Future work employing advanced proteomic approaches, specific antibodies, or biosensors will be crucial to unequivocally establish the activation status and protein‐level dynamics of the hypoxia signaling pathway during FLS2‐mediated immunity.

### 3.5 ERF13 is a Key Downstream Regulator in FLS2 Signaling

Previous studies have established that ERF15, ERF11, and ERF112 are involved in plant immune and stress responses. For instance, ERF15 functions as a potent transcriptional activator that enhances ABA‐mediated cold tolerance in tomato and contributes to JA burst upon herbivory attack [62, 63]. Similarly, ERF11 plays a critical role in plant immunity: its transcriptional activation of BT4 is essential for SA/ET‐regulated resistance against *Pst* DC3000, while MdERF11 enhances plant defense against the pathogen *B. dothidea* by modulating the SA biosynthesis pathway [64, 65]. Moreover, VqERF112 has been shown to positively regulate resistance to *Pseudomonas syringae* and *Botrytis cinerea* by modulating the expression of defense‐related genes responsive to SA and JA/ET signaling, and it has also been identified as a candidate regulator of sucrose accumulation in sugarcane [66]. In contrast, ERF13 has been primarily studied in the context of auxin signaling, where it regulates lateral root development. Its potential role in plant immune responses remains largely unexplored. Therefore, this study focuses on investigating the function of ERF13 in the flg22‐triggered immune signaling pathway. ERF transcription factors, particularly ERF13, play pivotal roles in plant defense [54, 63]. Flg22 markedly upregulated *ERF13* expression in WT but not *fls2* mutants (Figure S17). *erf13‐1* and *erf13‐2* mutants exhibited enhanced flg22 tolerance, while *ERF13* overexpression lines showed severe growth inhibition (Figure 6), confirming ERF13 acts downstream of FLS2 to positively regulate flg22 responses.

However, we observed increased susceptibility of *erf13* mutants to *Psm* and *Psm/AvrRpt2* versus WT. This discrepancy may stem from different inoculation concentrations (OD600 = 0.0001/0.002 here vs. OD600 = 0.01). We speculate higher pathogen concentrations saturate immune responses, masking genotypic differences [70].

### 3.6 Loss of FLS2 Rewires Immune Homeostasis to Enhance Effector‐Triggered Immunity

Pattern recognition receptors (PRRs) like FLS2 initiate PTI upon pathogen detection, while nucleotide‐binding leucine‐rich repeat receptors (NLRs) activate the more robust ETI. While these pathways were once viewed as distinct, emerging evidence suggests they synergistically enhance overall immunity [70, 86, 87]. FLS2 recognizes flg22 and competitively inhibits signaling from *Aspergillus oryzae*‐derived Ax21 peptide [88]. *Pseudomonas syringae pv. tomato *(Pst) may suppress flg22 responses [89], and flg22‐induced PTI attenuates ETI triggered by AvrRpt2 via the MPK3/MPK6‐WRKYs‐PP2Cs module (PTI‐mediated ETI suppression [PES]; [51]), revealing complex PTI‐ETI interactions.

In this study, we revisited the role of FLS2 in anti‐bacterial immunity by challenging loss‐of‐function mutants with both *Psm* (which primarily triggers PTI) and *Psm AvrRpt2* (which triggers ETI). We found that *fls2* mutants exhibited enhanced resistance to *Psm*, showing lower pathogen loads than WT (Figure 5A,C). Following *Psm AvrRpt2* infection, *fls2* mutants not displayed more severe chlorosis and necrotic lesions but, harbored significantly lower bacterial titers than wild‐type plants (Figure 5B,D). This finding indicates that the absence of FLS2 leads to a hyperactivation of AvrRpt2‐RPS2‐mediated ETI, resulting in enhanced restriction of pathogen growth. This aligns with our conclusion in the results section, suggesting that the loss of a key PTI component can rewire immune homeostasis, unleashing a more potent compensatory ETI response.

One plausible explanation lies in the phenomenon of PTI‐mediated ETI suppression (PES) [51]. In WT plants, a functional FLS2 pathway may actively restrain the amplitude of downstream ETI to prevent excessive, autoimmunity‐like reactions and maintain a balance between growth and defense. Upon removal of FLS2, this tonic suppression is lifted, allowing the RPS2‐mediated ETI pathway to respond more aggressively when activated by its cognate effector, AvrRpt2. In this context, FLS2 functions not only as an initiator of immunity but also as a homeostatic brake or modulator of NLR‐mediated responses. The enhanced resistance in *fls2* mutants is therefore not due to FLS2's direct role in ETI synergy, but rather a consequence of systemic immune reconfiguration following its loss. This interpretation is further supported by the enhanced ETI responses observed in *ate1* and *erf13* mutants (Figures 5 and 7), suggesting that multiple components of the PTI signaling network may converge to regulate the magnitude of NLR‐mediated defenses.

### 3.7 flg22 Modulates Growth via Cell Cycle Progression

Cell cycle progression is linked to immune responses [68]. Effector‐triggered immunity employs programmed cell death (PCD) to control proliferation and fate [60]. Dysregulation of the anaphase‐promoting complex/cyclosome (APC/C) via overexpression of its inhibitors OSD1/UVI4 enhances immunity and *R* gene expression [90]. Flg22 treatment increased 4C, 8C, and 16C cells (Figure S23A,B), suggesting immune‐induced endoreduplication [91]. Increased S/M‐phase cells, downregulated cell cycle inhibitors, and elevated ploidy collectively indicate flg22 promotes cell cycle progression in cotyledons.

The nuclear envelope protein CPR5 interacts with cyclin‐dependent kinase inhibitors (CKIs), hyperactivating E2F to negatively regulate ETI/PCD [92]. CPR5 antagonizes UV‐B‐INSENSITIVE 4 (UVI4)/OMISSION OF SECOND DIVISION (OSD1): UVI4/OSD1 inhibit ANAPHASE‐PROMOTING COMPLEX/CYCLOSOME (APC/C) via CELL CYCLE SWITCH PROTEIN 52 (CCS52), while CPR5 transcriptionally represses cell cycle components [93]. *sim smr* and *e2fabc *mutants showed stronger flg22 growth inhibition and immune responses, while *cpr5* was less sensitive (Figures S18, S19, and S24), suggesting cell cycle defects enhance flg22 responses and that *SIM*, *SMR*, *E2FB*, and *E2FC* participate in FLS2‐mediated regulation of cotyledon development. Flg22 altered ploidy and cell cycle gene expression in WT but not in less sensitive *ate1* and *erf13‐1* mutants (Figure S23), indicating flg22 impacts cell cycle progression (including endoreduplication) to regulate growth.

### 3.8 Concluding Model

Based on these findings, we propose a mechanistic framework (Figure S26): flg22 perception by FLS2 activates immune signaling, suppressing epidermal and mesophyll cell development in cotyledons and triggering ROS accumulation and expression of hypoxia responses genes. ROS and hypoxia signaling converge to modulate ERF13‐dependent transcription, orchestrating growth/developmental gene expression and cell cycle progression. Immune and hypoxia signals cooperatively modulate growth by disrupting cell cycle regulation, forming an integrated immune‐hypoxia‐cell cycle signaling network.

## 4 Methods

### 4.1 Plant Material and Growth Conditions

Seeds of the *fls2* (*SALK_141277*) and *ate1* (*SALK_023492C*) were obtained from the *Arabidopsis* Biological Resource Center. *erf13‐1* (*GK_724B09*) and *erf13‐2* (*GK_121A12*) were obtained from Prof. Zhaojun Ding (School of Life Sciences, Shandong University). Homozygous T‐DNA insertion lines were confirmed by polymerase chain reaction (PCR) using gene‐specific and T‐DNA‐specific primers [67, 94] (Table S9). The homozygous *Arabidopsis* lines expressing *35S::PSBO1‐YFP* and *35S::EPFL9‐YFP* used in this study were previously generated and molecularly characterized and are stable transgenic materials [45]. *Arabidopsis* seedling cultivation commenced with sowing. Under sterile conditions in a laminar flow hood, seeds were individually sown onto plates containing 1/2× Murashige and Skoog (MS) medium. The sowing procedure consisted of the following steps: First, seeds were surface‐sterilized. An appropriate quantity of seeds, determined by experimental design, was placed in a 1.5 mL sterile microcentrifuge tube. Seeds were treated with 75% (v/v) ethanol for 5 min, followed by treatment with 50% (v/v) Dettol solution for 10 min; tubes were gently inverted every 3 min during treatments to ensure thorough sterilization. Second, seeds were rinsed five times with sterile distilled water to remove residual sterilant. Third, seeds were individually sown onto 1/2× MS medium plates. Briefly, seeds suspended in water were aspirated into a sterile pipette tip. The tip was then detached, and seeds were gently deposited onto the agar surface using the water meniscus for control, ensuring even distribution and avoiding clumping or spillage. Sown plates were sealed with porous surgical tape and subjected to stratification at 4°C in darkness for 3 days. Stratified plates were then transferred to a growth chamber (19–22°C; 16 h light/8 h dark photoperiod) for germination. The day of radicle emergence (germination) was designated as Day 1. For soil cultivation, 6‐day‐old seedlings were transplanted into pots containing a 1:3 (v/v) mixture of nutrient soil and vermiculite.

### 4.2 flg22 Treatment of *Arabidopsis* Seedlings

flg22 treatments were performed using a hydroponic system. Three‐day‐old seedlings (post‐germination) exhibiting uniform growth and health were selected under sterile conditions and transferred to 24‐well plates. Each well contained 1.5 mL of liquid medium and two seedlings. Control seedlings were grown in liquid 1/2× MS medium. Seedlings for flg22 treatment were grown in liquid 1/2× MS medium supplemented with 1 µM flg22 peptide. Both control and treated seedlings were maintained in the growth chamber for 3 days prior to sampling. The flg22 peptide stock solution was prepared by dissolving lyophilized powder in sterile distilled water and stored at ‐20°C. The flg22 treatment solution was prepared using sterile liquid 1/2× MS medium.

### 4.3 Microscopy

Seedlings were stained with 10 µg ml^−1^ propidium iodide (PI) (P4170, Sigma) for 3 min prior to imaging. Fluorescence in samples was detected using a confocal laser scanning microscope (Zeiss, LSM980, Germany). The PI signal was visualized at wavelengths of 610 to 630 nm.

### 4.4 Hypoxic Treatment of *Arabidopsis* Seedlings

Hypoxic treatment (11% O_2_) was similarly conducted using a hydroponic system. Three‐day‐old seedlings were transferred to liquid culture in 24‐well plates. The plates were placed within a sealed environmental chamber. Oxygen concentration within the chamber was reduced to 11% by infusing inert nitrogen gas (N_2_), while maintaining the same temperature and photoperiod as the standard growth chamber. Control plates were kept in the standard growth chamber (ambient O_2_ ≈ 21%). For combined hypoxic + flg22 treatment, plates containing seedlings already exposed to flg22 were transferred to the hypoxic chamber. The hypoxic treatment for 3 days.

### 4.5 Inoculation of *Arabidopsis* Seedlings with Psm and Psm/AvrRpt2

Three‐week‐old, non‐bolting *Arabidopsis* seedlings were used for infection assays with *Psm* and *Psm/AvrRpt2*.

(1) Pathogen Activation: To restore virulence, bacterial stocks were revived approximately one week prior to inoculation. Thawed glycerol stocks of *Psm* were streaked onto King's B (KB) agar plates containing 100 µg/mL streptomycin. *Psm/AvrRpt2* stocks were streaked onto KB agar plates containing 100 µg/mL streptomycin and 10 µg/mL tetracycline. Plates were incubated at 30°C for 2 days. Bacteria were re‐streaked 2–3 times onto fresh selective plates.

(2) Inoculum Preparation: Bacterial suspensions were prepared by resuspending colonies from plates in sterile 10 mM MgSO_4_ using a pipette. Suspensions were adjusted to an optical density at 600 nm (OD_600_) of 0.0001 for *Psm* and 0.002 for *Psm/AvrRpt2* through serial dilution in sterile 10 mM MgSO_4_.

(3) Inoculation: The third to fifth rosette leaves per plant were selected for inoculation. Bacterial suspensions were pressure‐infiltrated into the abaxial (lower) leaf surface using a 1 mL needleless syringe. The petiole of each inoculated leaf was marked. Infiltration sites avoided major veins, and the infiltration spot was gently pressed to ensure even spread of the suspension throughout the leaf. Two leaves per plant and 12 plants per genotype were inoculated.

(4) Leaf Extract Preparation: Inoculated leaves were harvested at 0 days post‐inoculation (dpi; approximately 1 h post‐inoculation) and 3 dpi. Phenotypes were documented photographically. Leaf discs (∼8 mm diameter) were excised using a standard cork borer. Two discs were placed in a 1.5 mL microcentrifuge tube containing 500 µL of 10 mM MgSO_4_ and ceramic grinding beads. Tissues were homogenized thoroughly using a bead mill. Six replicate tubes were prepared per genotype.

(5) Pathogen Cultivation and Quantification: Leaf extracts were serially diluted (tenfold increments, 6 dilutions) in a 96‐well plate. Specifically, 20 µL of undiluted extract was transferred to a well containing 180 µL of 10 mM MgSO_4_, mixed, and 20 µL of this dilution was transferred to a new well containing 180 µL MgSO_4_, repeating to achieve the desired dilutions. Subsequently, 10 µL of each dilution was spotted and streaked linearly onto KB agar plates containing the appropriate antibiotics (100 µg/mL streptomycin for *Psm*; 100 µg/mL streptomycin + 10 µg/mL tetracycline for *Psm/AvrRpt2*).

(6) Pathogen Quantification and Statistical Analysis: Streaked plates were incubated at 30°C for approximately 2 days. Single bacterial colonies on plates showing countable numbers (typically the highest countable dilution) were enumerated. Bacterial titers within the inoculated leaf tissue (Colony Forming Units, CFU per cm^2^ or per leaf disc) were calculated based on the dilution factor, extract volume, and leaf disc area.

### 4.6 Protoplast Isolation

Approximately 50 mg of *Arabidopsis* cotyledons were finely chopped with a razor blade and transferred to a 24‐well plate well containing 0.5 mL of enzyme solution (0.5 mM CaCl_2_, 0.5 mM MgCl_2_, 5 mM MES, 1.5% Cellulase RS, 0.03% Pectolyase Y23, 0.25% BSA, actinomycin D [33 mg/l], and cordycepin [100 mg/l], pH 5.5). Protoplast isolation was performed by incubating the plate at room temperature on an orbital shaker (60 rpm) in darkness for 2 h. During incubation, a small aliquot of the digest was periodically examined under a microscope to monitor cell wall digestion; incubation time was adjusted if necessary. Following digestion, an equal volume of W5 solution (2 mM MES,154 mM NaCl, 125 mM CaCl_2_ and 5 mM KCl, pH 5.7) was added. The mixture was filtered through a 200‐µm nylon mesh. The filtrate was centrifuged at 40 × g for 5 min. The supernatant was discarded, and the pellet was resuspended in 0.5 mL of ice‐cold W5 solution. After incubation on ice for 30 min, the W5 solution was removed. The isolated protoplasts were washed three times with 8% mannitol buffer to remove Mg^2+^. Cells were then filtered through a 40 µm cell strainer. Cell activity was detected by trypan blue staining and cell concentration was measured with a hemocytometer.

### 4.7 Semi‐Thin Sectioning

Prior to semi‐thin sectioning of *Arabidopsis* cotyledons, LR White resin pre‐polymerization solution was prepared by adding 1.95% (w/w) benzoyl peroxide initiator to LR White resin and stirring magnetically overnight until fully dissolved.

(1) Fixation: Tissue samples were fixed in 4% (v/v) glutaraldehyde solution. Vacuum infiltration was applied to ensure complete immersion of samples. Fixation proceeded overnight at room temperature.

(2) Embedding: Fixed samples were embedded in molten agarose. Once cooled but still liquid, samples were oriented within the agarose. After solidification, agarose blocks containing samples were trimmed to cubes with edges <1 cm.

(3) Dehydration and Staining: Agarose blocks were dehydrated through a graded ethanol series (30%, 50%, 70%, 90%; 7 min per step). Blocks were then stained for 5 min in 2% (w/v) Eosin Y dissolved in absolute ethanol. Following staining, blocks were dehydrated twice in absolute ethanol (7 min per wash).

(4) Infiltration: Samples were infiltrated with LR White resin. Initially, blocks were immersed in a 50% (v/v) LR White resin solution in absolute ethanol for 1 h. This was replaced with 100% LR White resin for 5 h. Fresh 100% LR White resin was added, and infiltration continued overnight at room temperature.

(5) Polymerization and Sectioning: Samples and paper labels were placed into embedding capsules. Pre‐polymerized LR White resin was added to cover the samples, and capsules were sealed. Polymerization was carried out in a 60°C oven for 10–12 h. After complete hardening, polymerized blocks were sectioned (approximately 0.5–1 µm thickness) using a Leica EM UC7 ultramicrotome. Sections were collected on glass slides, stained with 1% (w/v) crystal violet, and examined/photographed using light microscopy or scanning electron microscopy.

### 4.8 Flow Cytometry

(1) Plant Leaf Cell Size Measurement: Based on flow cytometry principles, the forward scatter (FSC) signal intensity correlates with cell size/volume. Therefore, cell size was assessed by flow cytometry. Protoplasts were isolated from leaf samples. Protoplast suspensions were analyzed using an analytical flow cytometer (Beckman Coulter CytoFlex S). Cells were excited with a 440‐nm argon‐ion laser. Data from 10,000 cells per sample were acquired and analyzed using CytExpert 2.4 software.

(2) Plant Leaf Chromosome Ploidy Detection: Flow cytometry, utilizing a 488‐nm, 15‐mW solid‐state laser, excites propidium iodide (PI)‐stained DNA molecules, inducing fluorescence emission. Measurement of fluorescence intensity, proportional to DNA content, generates DNA content (ploidy) histograms, enabling determination of sample ploidy composition. Fresh cotyledon tissue (∼0.05 g) was finely chopped in a petri dish with 400 µL of ice‐cold HEPES isolation buffer. The homogenate was transferred to a 1.5 mL microcentrifuge tube, vortexed vigorously for 20 s, and incubated on ice for 15 min. An additional 300 µL of ice‐cold HEPES isolation buffer was added, and the mixture was filtered through a 300‐µm nylon mesh. To the filtrate, 10 µL of RNase solution (250 µg/mL) was added, vortexed, and incubated on ice for 15 min. Finally, 10 µL of PI staining solution (1 mg/mL) was added, and samples were stained in the dark for 8 min prior to analysis. Data from 10,000 cells per sample were acquired and analyzed using CytExpert 2.4 software.

### 4.9 scRNA‐seq Analysis

(1) scRNA‐seq Library Preparation: scRNA‐seq libraries were constructed using the 10× Genomics Chromium Next GEM Single Cell 3ʹ Reagent Kits v3.1. Protoplast suspensions (from leaf samples) were adjusted to a concentration of 700–1200 cells/mL. Single‐cell gel beads‐in‐emulsion (GEMs), each containing a single cell tagged with a unique barcode, were generated. Briefly, a Master Mix (31.8 µL) was prepared on ice. Cell suspension was added and mixed. Subsequently, 70 µL of the cell/master mix was loaded into the second well of a Chromium chip. Gel Beads (50 µL) were loaded into the third well, and Partitioning Oil (45 µL) into the fourth well. A gasket was applied, and the chip was loaded into the Chromium Controller for GEM generation (∼18 min). Following GEM generation, 100 µL of GEMs were transferred to pre‐chilled PCR tubes for reverse transcription (RT). RT products were purified using Recovery Agent, Dynabeads Cleanup Mix, and Elution Buffer I. Purified cDNA was amplified using cDNA Amplification Reaction Mix. Amplified cDNA was purified using SPRIselect beads and Elution Buffer (EB). Purified cDNA concentration and quality were assessed using Qubit fluorometry and Agilent 2100 Bioanalyzer; samples were stored at ‐20°C for up to 4 weeks. cDNA was fragmented, end‐repaired, and A‐tailed using Fragmentation Mix, followed by PCR amplification. Products were purified using SPRIselect beads to remove flanking regions. Adaptor ligation was performed using Adaptor Ligation Mix. Ligated products were purified using SPRIselect beads and EB buffer. Final library amplification (PCR enrichment) was performed. Enriched libraries were purified using SPRIselect beads. Libraries passing quality control were used for high‐throughput sequencing.

(2) Sequencing Data Quality Control and Gene Expression Quantification: Raw scRNA‐seq data were processed using the 10× Genomics Cell Ranger software suite (v7.1.0). Initial processing included read quality assessment and alignment to the reference genome (*Arabidopsis thaliana* TAIR10) for gene expression quantification (count matrix generation). Primary quality control (QC) metrics assessed included the number of high‐quality cells, median genes per cell, and sequencing saturation. Further QC involved the removal of low‐quality cells, cells with unique feature (gene) counts (nFeature_RNA) less than 200 or greater than 7500 were filtered out, as were cells with a percentage of mitochondrial reads (percent.mito) exceeding 1%. Doublets were specifically detected and removed using the Scrublet algorithm (v0.2.3) with an expected doublet rate of 0.08.

(3) Dimensionality Reduction and Clustering Analysis: Principal Component Analysis (PCA) was performed on the normalized gene expression matrix for linear dimensionality reduction. The top principal components (PCs) were selected for subsequent non‐linear dimensionality reduction using UMAP, visualized in two dimensions. Cell clusters were identified using the Shared Nearest Neighbor (SNN) modularity optimization clustering algorithm implemented in Seurat (resolution parameter of 0.8).

(4) Cell Type Annotation: Putative marker genes for each cluster, defined as genes significantly upregulated within a cluster compared to all other clusters, were identified using the FindAllMarkers function in the Seurat package (p‐value < 0.05, minimum log fold‐change threshold). Expression patterns of identified marker genes were visualized using VlnPlot (violin plots) and FeaturePlot (spatial expression on UMAP) functions to validate cluster identity. Final cell type identities were determined by marker gene expression.

(5) Differential Gene Expression and Functional Enrichment Analysis: DEGs between specific cell clusters or conditions were identified using the FindMarkers function in Seurat (Wilcoxon rank sum test; adjusted p‐value < 0.05, |log_2_(fold change)| > 1.5).

(6) GO and KEGG analyses: KEGG and GO analyses were performed in Metascape (http://metascape.org). The DEGs in each comparison group were first identified and subsequently uploaded to Metascape as described in the user's manual. Afterwards, both GO and KEGG analyses were performed automatically in Metascape to obtain the results.

(7) Pseudotime Trajectory Analysis: Pseudotime analysis was conducted using Monocle 3. The Seurat object containing normalized expression data and cluster identities was converted into a CellDataSet object using the importCDS function. Highly variable genes ordered along potential trajectories (differentialGeneTest, q‐value < 0.01) were selected for dimensionality reduction (DDRTree). The developmental trajectory was inferred using the minimum spanning tree (MST) algorithm. Gene expression dynamics along the pseudotime trajectory were visualized using the plot_genes_in_pseudotime function.

(8) Transcriptional Regulatory Network Analysis: The transcription factors can be identified from the DEGs of each of comparison groups according to the *Arabidopsis* transcription factor data base, which can be downloaded from PlantTFDB (http://planttfdb.cbi.pku.edu.cn/). Then the regulatory relationship between transcription factor and target genes can be determined according to the interaction data base, which can be downloaded from http://plantregmap.gao‐lab.org/download.php. Then the transcription factor network can be constructed with Cytoscape program (http://www.cytoscape.org/).

### 4.10 RNA Extraction and Reverse Transcription in *Arabidopsis*

(1) RNA Extraction: Total RNA was extracted from *Arabidopsis* seedling using the RNAprep Pure Plant Kit (Vazyme Biotech). To prevent RNA degradation by ubiquitous RNases, all consumables (pipette tips, microcentrifuge tubes, grinding beads) were treated with 0.1% (v/v) diethyl pyrocarbonate (DEPC)‐treated water. All procedures were performed within a RNase decontaminated laminar flow hood or fume hood.

(2) Reverse Transcription: RNA concentration and purity (A_260_/A_280_ ratio) were determined using a NanoDrop spectrophotometer prior to reverse transcription (RT). Genomic DNA (gDNA) was removed from total RNA using the recommended DNase I treatment protocol included in the HiScript III RT SuperMix for qPCR (+gDNA wiper) kit (Novoprotein). Reaction components were assembled in RNase‐free 200 µL tubes; the volumes of template RNA and RNase‐free water were calculated based on measured concentrations. Reverse transcription was performed strictly according to the manufacturer's instructions.

### 4.11 qPCR Analysis of Gene Expression

Relative gene expression levels were quantified using the NovoStart SYBR qPCR SuperMix Plus (Novoprotein) on a qTOWER3G real‐time PCR system (Analytik Jena). Reaction mixtures were precisely prepared in 96‐well plates. Plates were centrifuged briefly to mix contents and eliminate air bubbles. qPCR amplification was performed using the following cycling conditions: initial denaturation at 95°C for 5 min; 40 cycles of 95°C for 15 s, 60°C for 30 s (annealing/extension), followed by a melt curve analysis step (60°C to 95°C, increment 0.5°C per 5 s). Relative quantification was calculated using the 2^−ΔΔCt^ method, normalizing target gene expression to the reference gene *ACTIN* (*AT1G13180*).

### 4.12 Fluorescent Detection of Intracellular ROS

Intracellular ROS levels were detected using the fluorescent probe 2’,7’‐dichlorodihydrofluorescein diacetate (H_2_DCFDA). H_2_DCFDA is a cell‐permeable, non‐fluorescent probe that diffuses freely into cells. Within the cytoplasm, it is hydrolyzed by endogenous esterases, cleaving the acetate groups to generate non‐fluorescent H_2_DCF. Subsequently, H_2_DCF is oxidized by various intracellular ROS, yielding the highly fluorescent product 2’,7’‐dichlorofluorescein (DCF). The fluorescence intensity of DCF is proportional to the intracellular ROS levels. A 10 mM stock solution of H_2_DCFDA (Sigma‐Aldrich, Cat. No. D6883) was prepared in anhydrous dimethyl sulfoxide (DMSO) and stored protected from light at –20°C. All subsequent procedures were performed under light‐protected conditions. Immediately prior to each experiment, the stock solution was diluted with pre‐cooled phosphate‐buffered saline (PBS, pH 7.4) to a final working concentration of 20 µM. Cotyledons of Arabidopsis seedlings were immersed in the 20 µM H_2_DCFDA working solution and incubated in the dark at 37°C for 20 min. Following incubation, the samples were transferred to fresh PBS and gently washed on a shaker three times for 5 min each to thoroughly remove any residual probe. The washed samples were then mounted on glass slides and immediately observed using a confocal laser scanning microscope (Zeiss **LSM** 880). DCF fluorescence was detected with an excitation wavelength of 488 nm, and the emission signal was collected within the range of 500–535 nm.

### 4.13 Measurement of the Cotyledon‐Hypocotyl Angle

The angle at the junction between the cotyledons and the hypocotyl was measured to quantify seedling morphology. For this purpose, phenotypic images of the seedlings were first acquired. Subsequently, the specific angle was manually measured by applying the Angle Tool within the ImageJ software package.

### 4.14 Statistical Analysis

All experiments were performed with at least three independent biological replicates unless otherwise specified in the figure legends. Data are presented as mean ± standard deviation (SD). For comparisons between two groups, statistical significance was determined using two‐tailed Student's *t*‐test. For multiple group comparisons, one‐way or two‐way analysis of variance (ANOVA) was performed followed by Tukey's post‐hoc test for pairwise comparisons, as indicated in the figure legends. Sample sizes (n) for each experiment are provided in the corresponding figure legends. Statistical significance was defined as *p < 0.05, **p < 0.01, ***p < 0.001. All statistical analyses were performed using GraphPad Prism (version 9.0) and R software with appropriate packages.

## Author Contributions

Conceptualization of the project: X.S. and Z.L. Experimental design: X.S. Performance of some specific experiments: Y.Z(Yaping Zhou), A.Q., L.K., L.Y., Q.Z., M.L.(Mengfan Li), C.L., H.L., Y.Z.(Yinpeng Zhang), J.L., M.L.(Mengyu Liao), and M.Z. Data analysis: Y.Z.(Yaping Zhou), X.F., B.W., W.K., M.Z., and Y.S. Manuscript drafting: Z.L. and S.X. Contribution to the editing and proofreading of the manuscript draft: Y.Z.(Yaping Zhou), and Z.L. All authors have read and approved the final manuscript.

## Conflicts of Interest

The authors declare no conflicts of interest.

## Supporting information




**Supporting File 1**: advs74740‐sup‐0001‐SuppMat.docx.

**Supporting File 2**: advs74740‐sup‐0002‐TableS1.xls.

**Supporting File 3**: advs74740‐sup‐0003‐TableS2.xlsx.

**Supporting File 4**: advs74740‐sup‐0004‐TableS3.xls.

**Supporting File 5**: advs74740‐sup‐0005‐TableS4.xlsx.

**Supporting File 6**: advs74740‐sup‐0006‐TableS5.xlsx.

**Supporting File 7**: advs74740‐sup‐0007‐TableS6.xls.

**Supporting File 8**: advs74740‐sup‐0008‐TableS7.xlsx.

**Supporting File 9**: advs74740‐sup‐0009‐TableS8.xlsx.

**Supporting File 10**: advs74740‐sup‐0010‐TableS9.xlsx.

## Data Availability

The data that support the findings of this study are available in the supplementary material of this article.
