# Supplementary material for: Single‐Cell Transcriptomics Reveals FLS2‐Dependent Hypoxia Signaling and ERF13‐Mediated Transcription During flg22‐Triggered Immunity
Source: Adv Sci (Weinh). 2026 Mar 9;13(28):e16380. doi: 10.1002/advs.202516380 (PMC13185882; doi:10.1002/advs.202516380)
Supplement: Supplementary file 1 — Supporting File 1: advs74740‐sup‐0001‐SuppMat.docx. [file ADVS-13-e16380-s001.docx]

**Supplemental Figures**


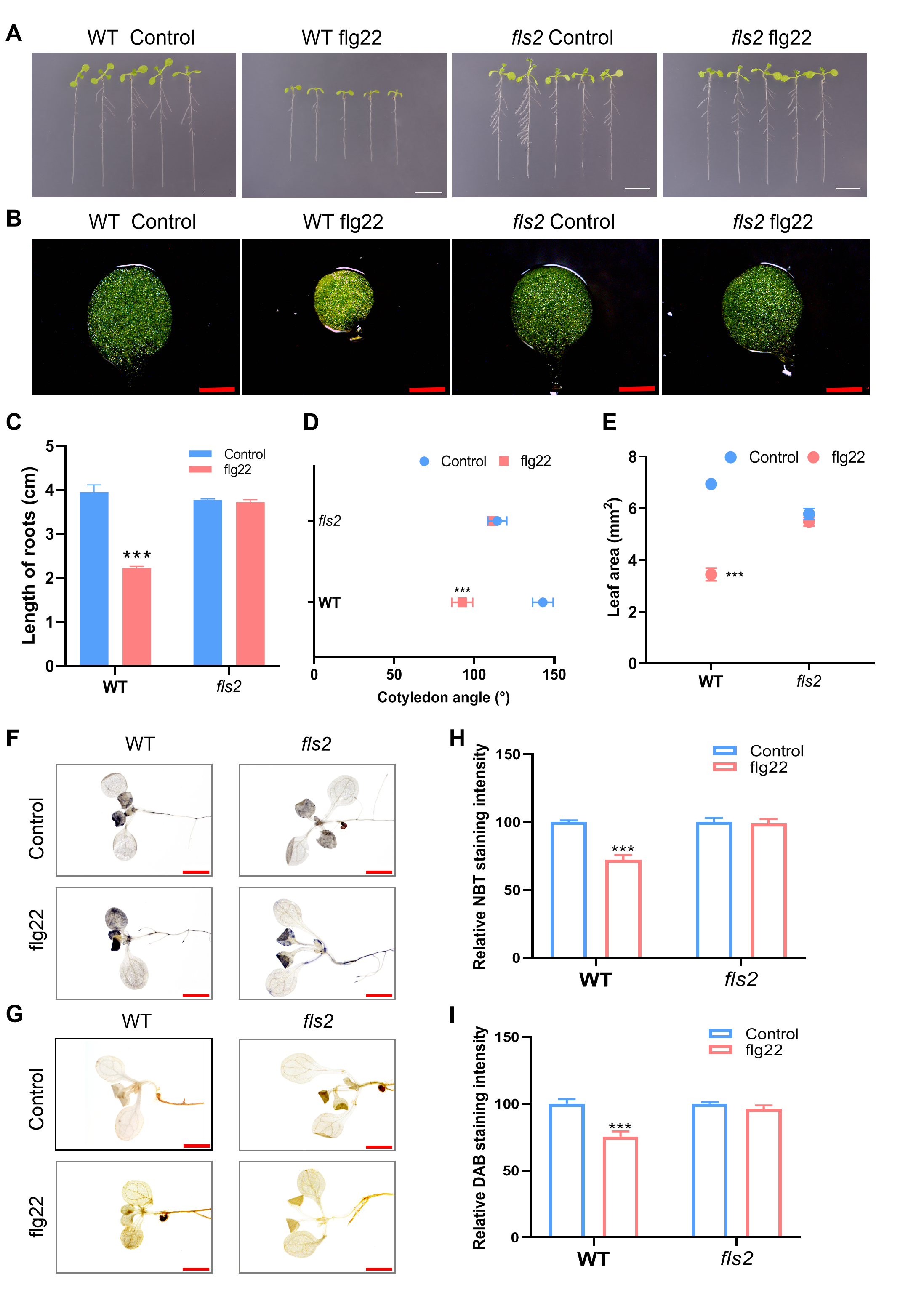


**Supplemental** ****Figure S1.** Effects of flg22 treatment on the development of wild-type (WT) and *fls2* mutant seedlings.**

Phenotypic analysis of 3-day-old WT and *fls2* mutant seedlings following 3 days of control or flg22 treatment.

****(A)**** Whole seedling morphology of WT and *fls2* mutant seedlings. Scale bar = 1 cm.
****(B)**** Cotyledon phenotype of WT and *fls2* mutant seedlings. Scale bar = 1 mm.
****(C)**** Quantitative analysis of root length of WT and *fls2* mutant seedlings in (A). Data represent mean ± SD (n=5). Statistical significance between treatment and control was determined by one-way ANOVA followed by Tukey's test (***p<0.001).

****(D)**** Measurement of cotyledon opening angle of WT and *fls2* mutant seedlings in (A).Data represent mean ± SD (n=5). Statistical significance between treatment and control was determined by one-way ANOVA followed by Tukey's test (***p<0.001).

****(E)**** Quantitative analysis of cotyledon area of WT and *fls2* mutant seedlings. Data represent mean ± SD (n=3). Statistical significance between treatment and control was determined by one-way ANOVA followed by Tukey's test (***p<0.001).

****(F)**** Detection of superoxide anion in WT and *fls2* mutant seedlings by Nitroblue tetrazolium (NBT) staining. Scale bar = 2 mm.
****(G)**** Detection of hydrogen peroxide in WT and *fls2* mutant seedlings by 3,3'-Diaminobenzidine (DAB) staining. Scale bar = 2 mm.

****(H)**** Statistical analysis of the grayscale value indicating NBT staining intensity in (F). A lower grayscale value represents darker NBT staining and a higher level of superoxide anion accumulation. Data are presented as mean ± SD (n = 3). The asterisk indicates a significant difference between treatment and control as determined by Student's *t*-test (***p < 0.001).
****(I)**** Statistical analysis of the grayscale value indicating DAB staining intensity in (G). A lower grayscale value represents darker DAB staining and a higher level of H_2_O_2_ accumulation. Data are presented as mean ± SD (n = 3). The asterisk indicates a significant difference between treatment and control as determined by Student's *t*-test (***p < 0.001).


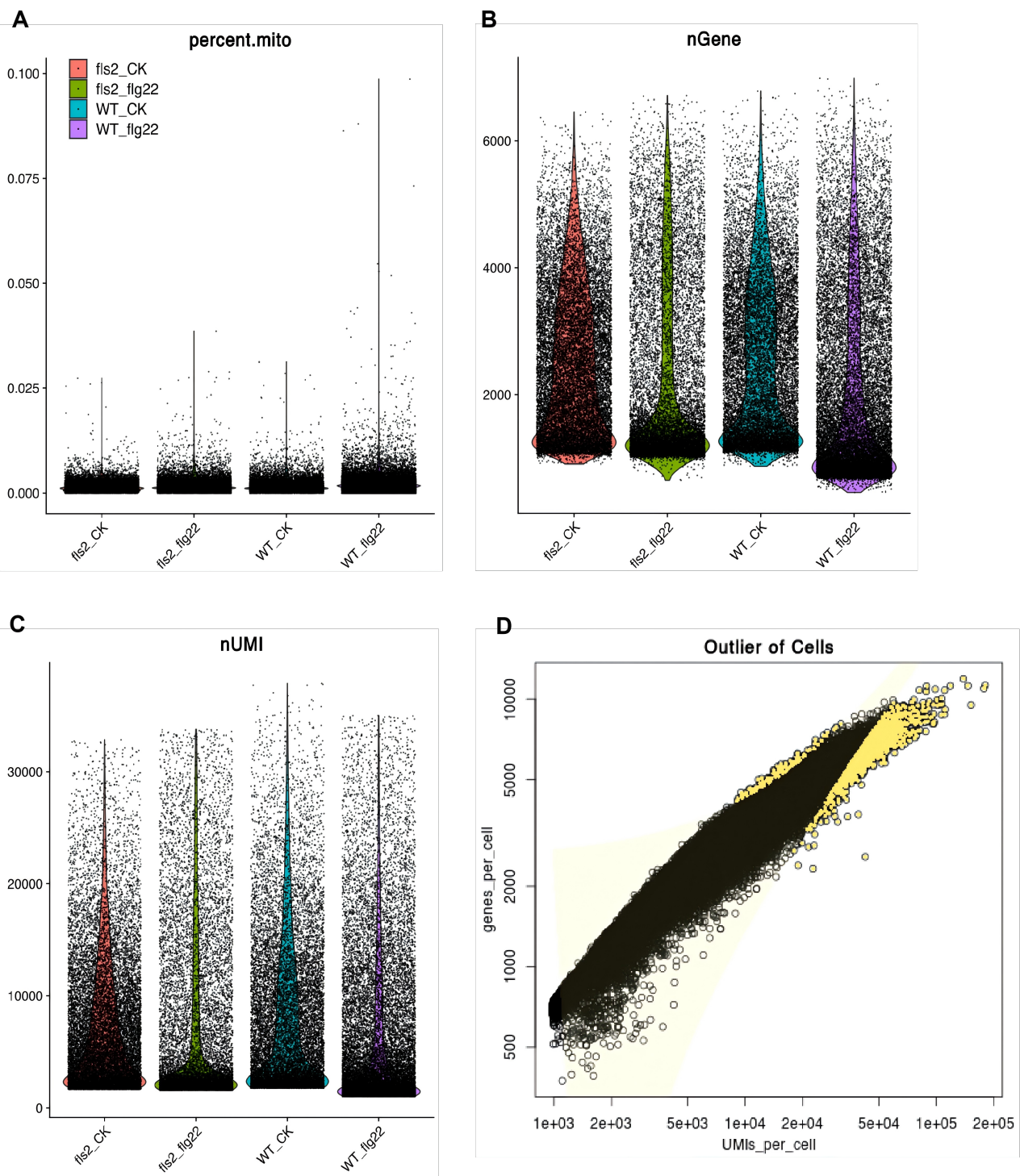


****Supplemental Figure S2.** Quality control metrics of scRNA-seq data.**

****(A)**** Distribution of mitochondrial gene content per cell across samples after quality filtering. The y-axis represents the percentage of mitochondrial genes relative to the total number genes detected per cell. Each point indicates the mitochondrial gene proportion in an individual droplet, with violin plots showing the overall distribution across all cells.

****(B)**** Distribution of detected genes (nGene) per cell following quality control. The y-axis displays unique molecular identifier (UMI) counts, representing transcript abundance.

****(C)**** Distribution of UMI counts (nUMI) per cell after quality filtering.

****(D)**** Generalized linear model-based outlier cell filtration. The x-axis shows nUMI counts per cell, while the y-axis represents nGene counts per cell. Colored points denote outlier cells that were excluded from downstream analyses.


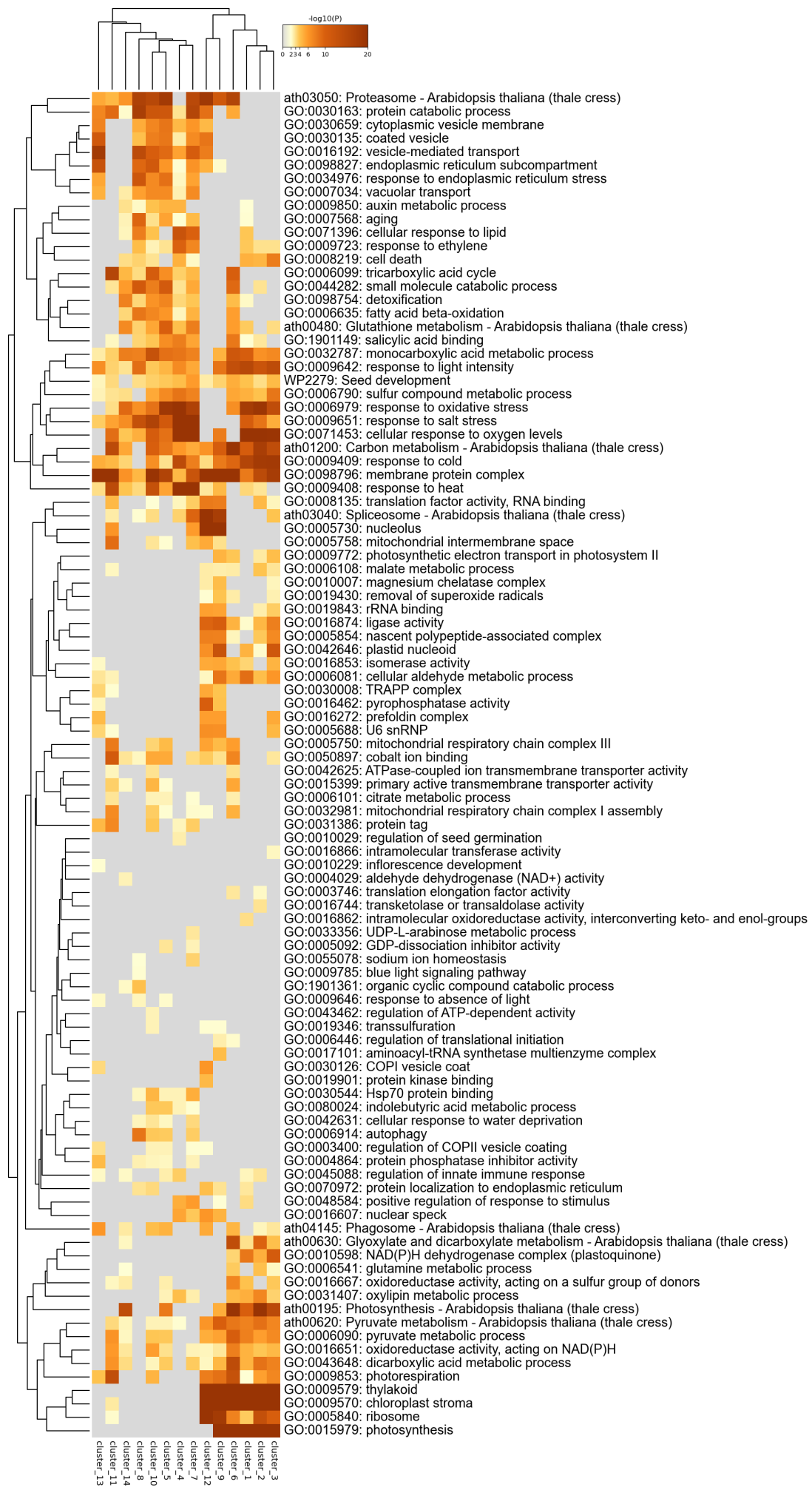


****Supplemental Figure S3.**** Gene Ontology (GO) enrichment heatmap of differentially expressed genes (DEGs) in each cell cluster.

Functional enrichment analysis of cluster-specific genes. This heatmap visualizes the top 100 most significantly enriched GO terms and KEGG pathways for each cluster. Rows represent individual terms or pathways, and columns represent cell clusters. The color gradient indicates the strength of the enrichment score (-log10(P-value)) for each term within a given cluster, highlighting the functional themes associated with each distinct cell population.


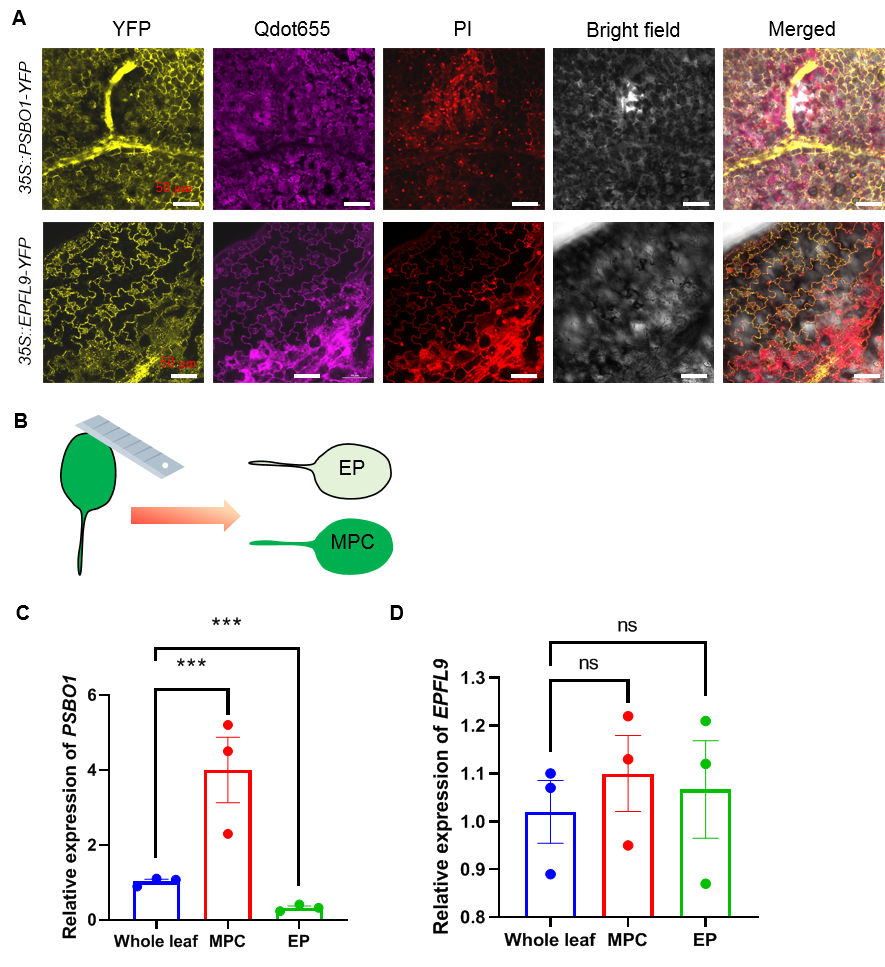


****Supplemental Figure S4.**** Cellular localization analysis of PSBO1 and EPFL9 in transgenic *Arabidopsis*.
(A) Confocal microscopy images of cotyledons from transgenic *Arabidopsis* seedlings expressing *35S::PSBO1-YFP* or *35S::EPFL9-YFP*. Fluorescence signals are shown as follows: YFP (Yellow Fluorescent Protein, Yellow), (Qdot655, magenta), and propidium iodide (PI) staining (red). Bright-field, merged, and composite images (YFP, PI, and bright-field) are also presented to illustrate tissue context and protein localization. Scale bar: 50 μm.

(B) Schematic diagram showing the isolation of epidermal and mesophyll cell tissues from *Arabidopsis* leaves.
(C) qPCR analysis of *PSBO1* gene expression in total leaf tissue, epidermal tissue, and mesophyll cells. Data are presented as mean ± SD (n = 3). Statistical significance between treatment and control was determined by one-way ANOVA followed by Tukey's test (***p<0.001).

(D) qPCR analysis of *EPFL9* gene expression in total leaf tissue, epidermal tissue, and mesophyll cells. Statistical significance between treatment and control was determined by one-way ANOVA followed by Tukey's test (ns: no significance).


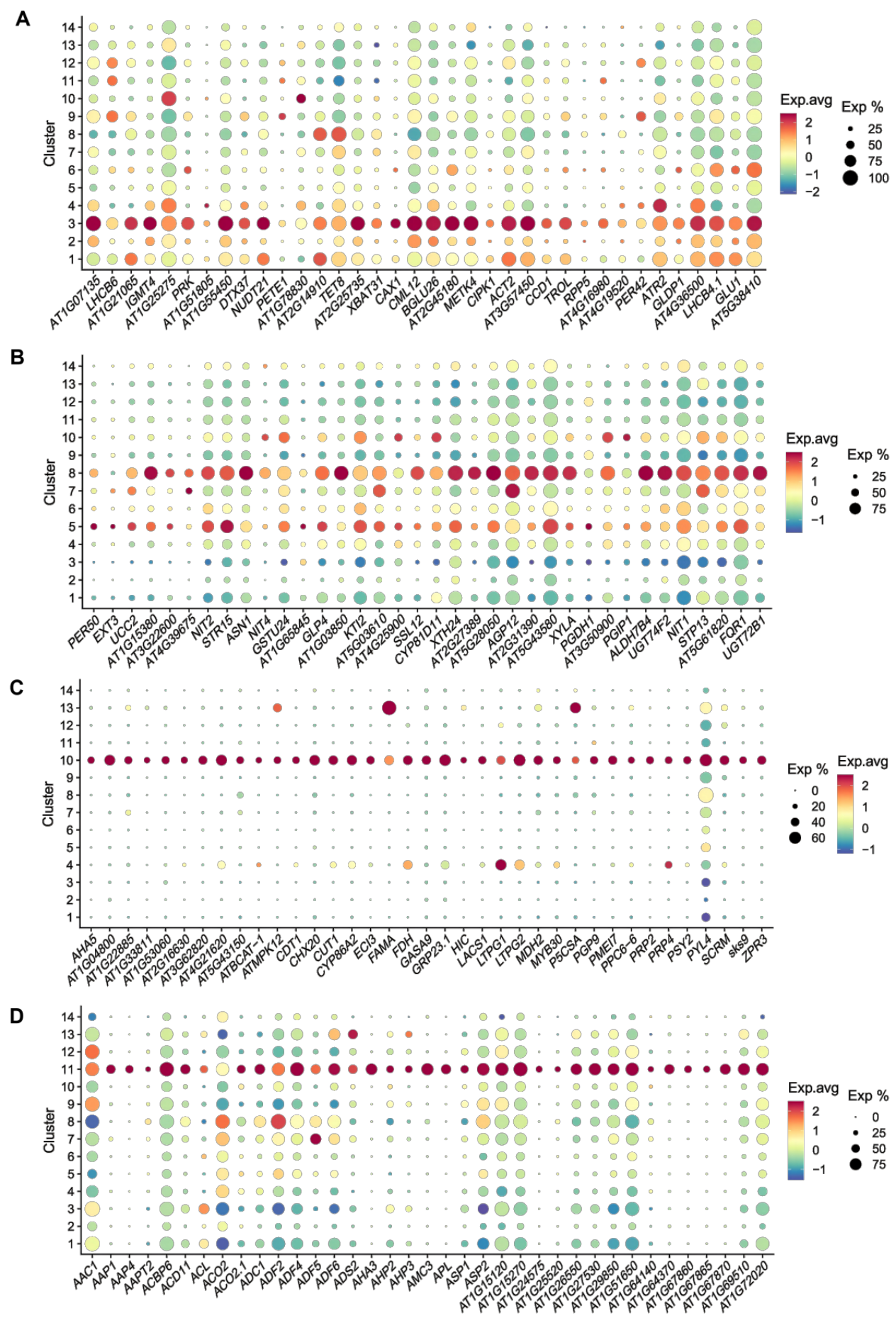


****Supplemental Figure S5.** Expression features of cluster-specific genes.**

**Dot plots visualize the genes uniquely expressed in three distinct cell types:**

**(A) Epidermal cells (cluster 3).**

**(B) Cortex cells (clusters 5 and 8).**

**(C) Guard cells (cluster 10).**

**(D) Phloem Companion cells (cluster 11)**

**In each plot, dot size corresponds to the percentage of expressing cells, and color represents the mean expression value.**


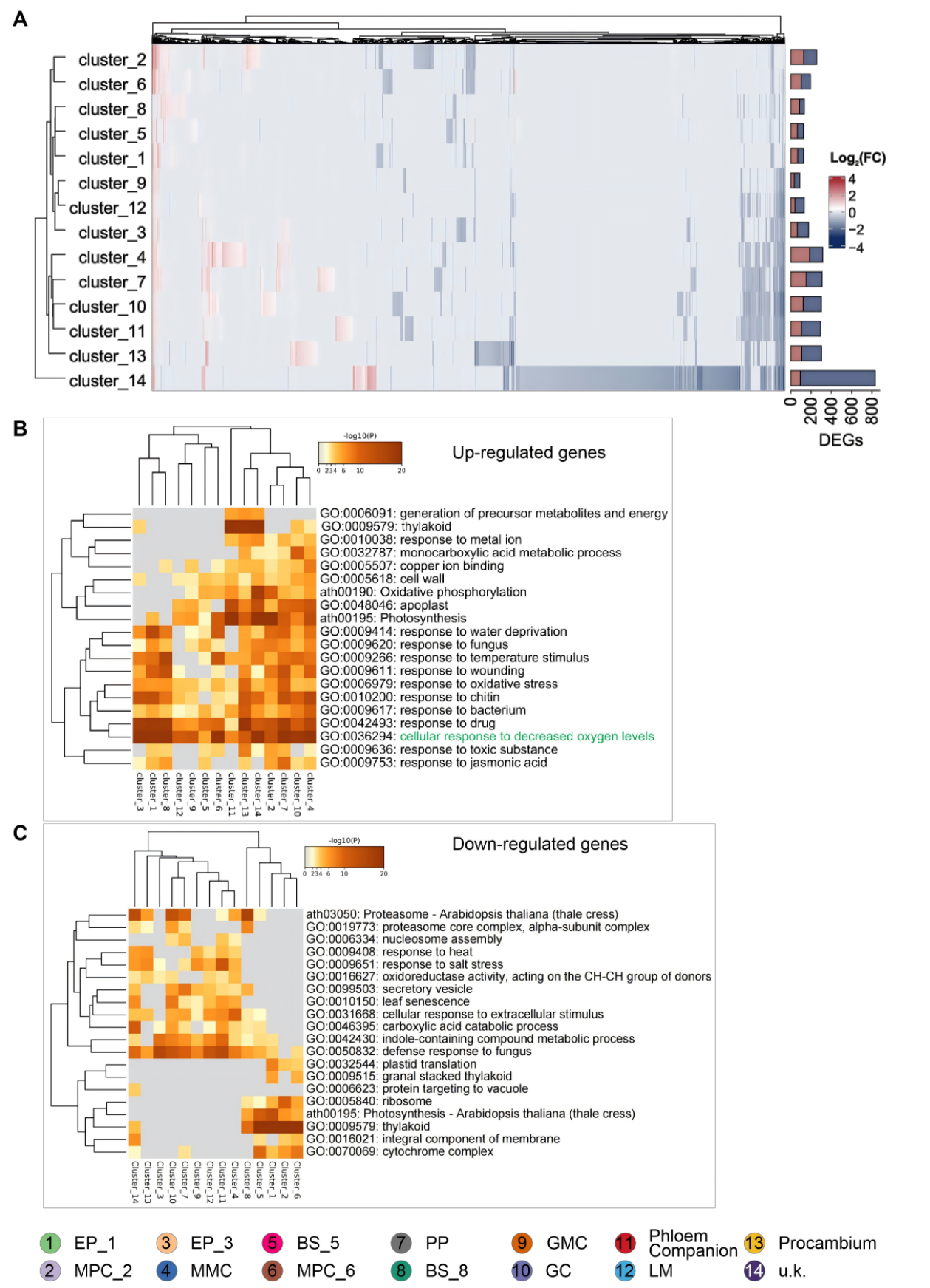


****Supplemental Figure S6.** Differential gene expression analysis between WT_flg22 and WT_CK across cell clusters.**

****(A)**** Heatmap displaying expression profiles of differentially expressed genes (DEGs) in distinct cell clusters. The number of DEGs in each cluster is indicated on the right. Gene expression levels are represented by a color gradient from blue (low) to red (high).

****(B)**** Gene Ontology (GO) enrichment heatmap of upregulated genes in different cell clusters.

****(C)**** GO enrichment heatmap of downregulated genes in different cell clusters.


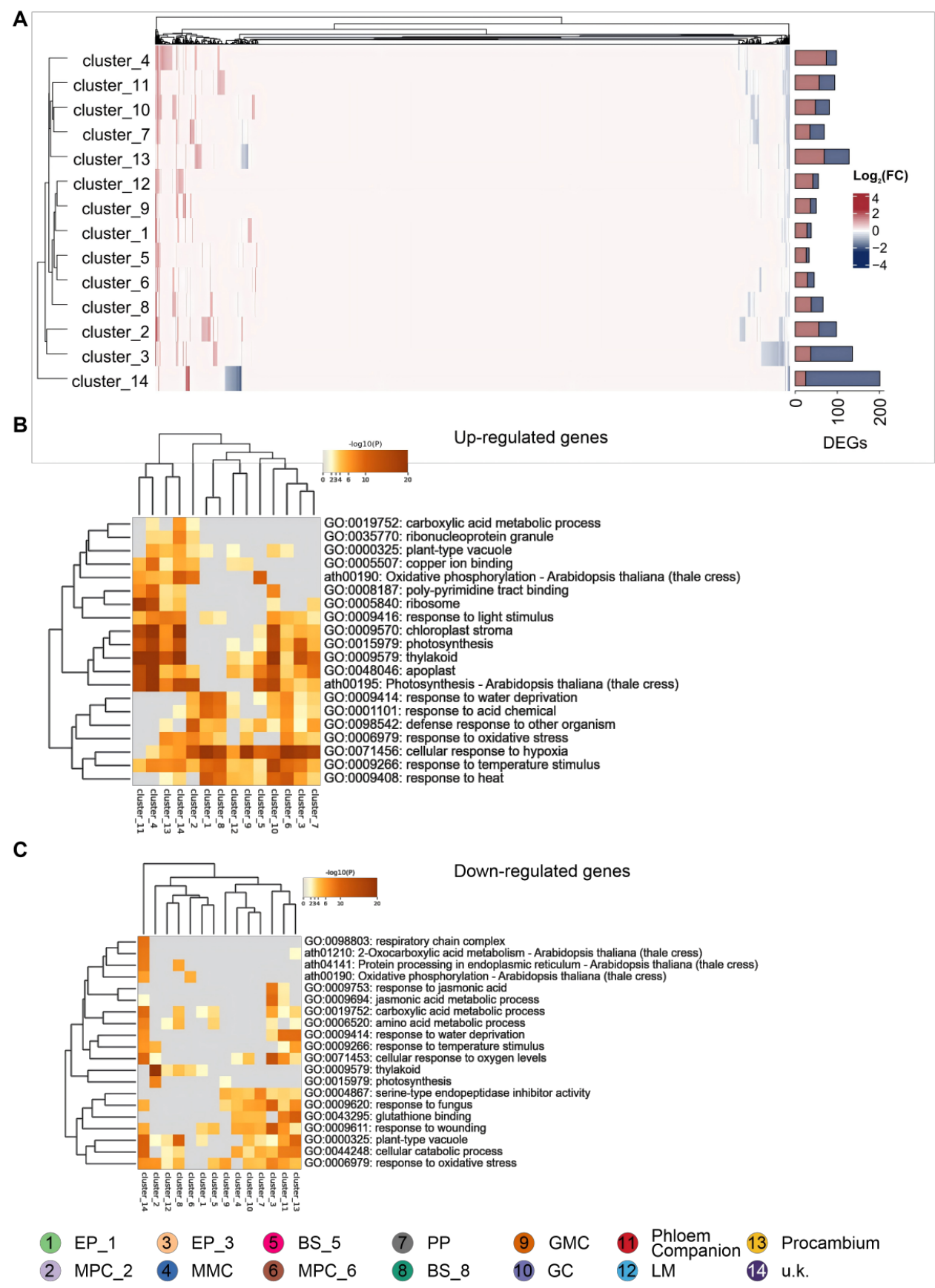


****Supplemental Figure S7.** Differential expression analysis between *fls2*_flg22 and *fls2*_CK across cell clusters.**

****(A)**** Heatmap illustrating expression profiles of DEGs in distinct cell clusters. The number of DEGs for each cluster is annotated on the right. Gene expression levels are color-coded, with blue indicating low expression and red representing high expression.

****(B)**** Heatmap of GO enrichment analysis for upregulated DEGs in each cell cluster.

****(C)**** Heatmap of GO enrichment analysis for downregulated DEGs in each cell cluster.


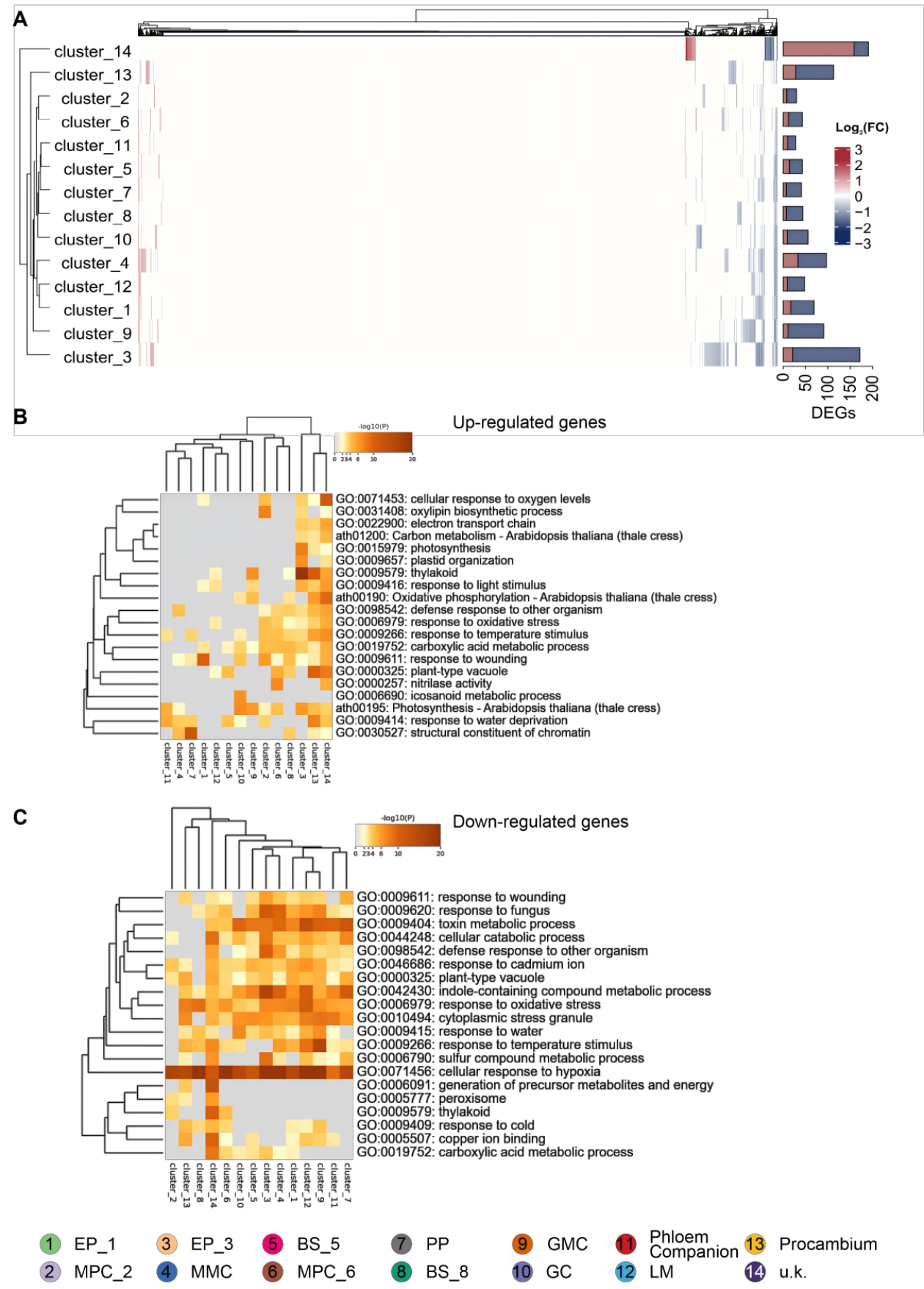


****Supplemental Figure S8.** Comparative DEG analysis between *fls2*_CK and WT_CK across cell clusters.**

****(A)**** Heatmap visualization of DEGs in respective cell clusters. The right axis indicates the quantity of identified DEGs per cluster, while expression levels are color-scaled from blue (low) to red (high).

****(B)**** Functional enrichment analysis via GO term heatmap for cluster-specific upregulated DEGs.

****(C)**** GO term heatmap analysis of downregulated DEGs across distinct cell clusters.


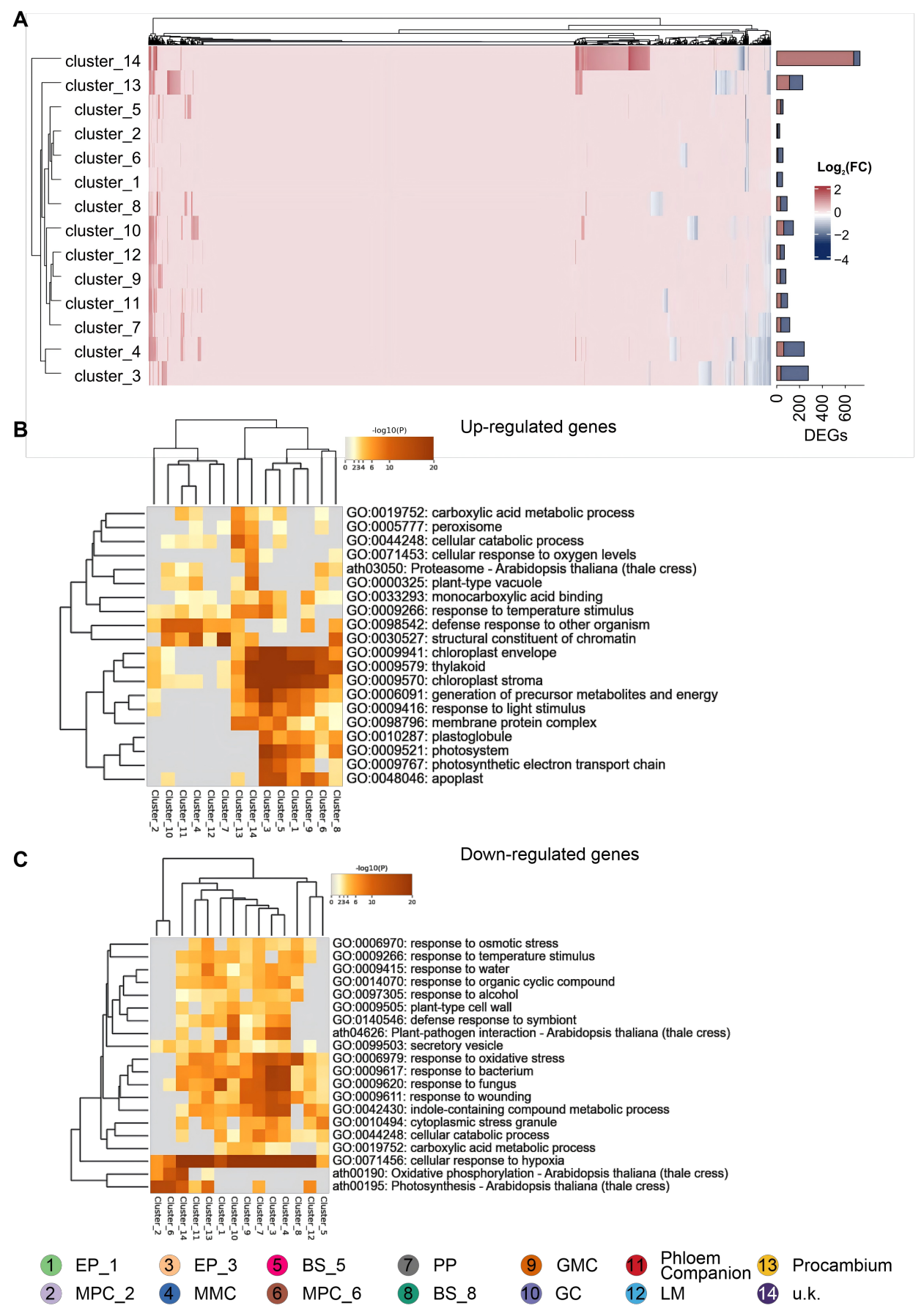


****Supplemental Figure S9.** Differential gene expression analysis between *fls2*_flg22 and WT_flg22 across cell clusters.**

****(A)**** Heatmap representation of DEGs across distinct cell clusters. The right panel indicates the number of DEGs identified per cluster, with gene expression levels color-coded using a blue (low) to red (high) gradient.

****(B)**** Gene Ontology (GO) enrichment heatmap analysis of upregulated DEGs in each cell cluster.

****(C)**** GO enrichment heatmap analysis of downregulated DEGs in each cell cluster.


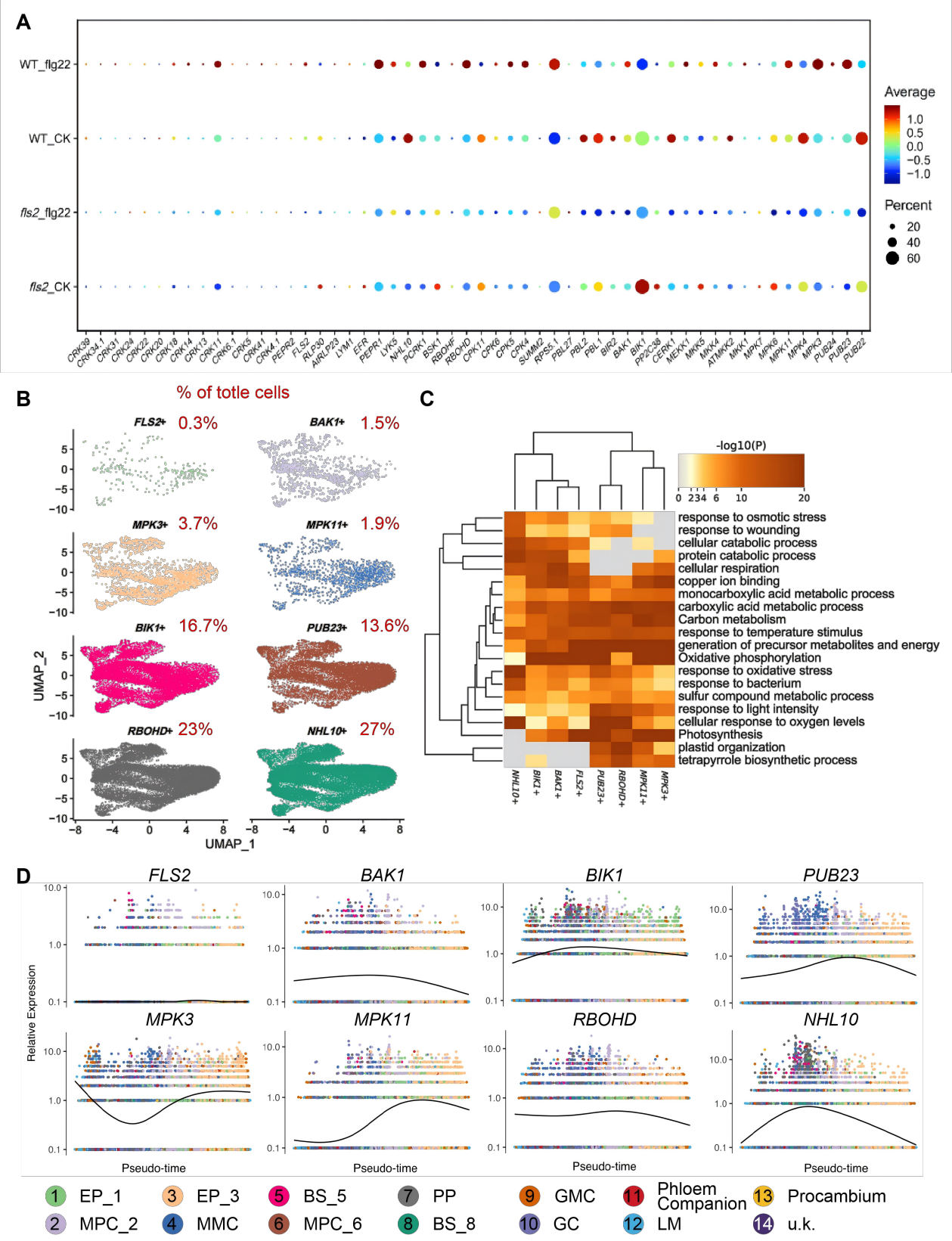


****Supplemental Figure S10.** Expression patterns of immune response-related marker genes.**

****(A)**** Dot plot showing the expression of immune response-associated genes. Gene expression levels are represented by color intensity, while the percentage of cells expressing each gene is indicated by dot size.

****(B)**** UMAP visualization of selected FLS2 signaling pathway marker genes across all cell types. The percentage of gene-expressing cells relative to the total cell population is annotated.

****(C)**** GO term enrichment heatmap of DEGs in cells expressing FLS2 signaling pathway marker genes.

****(D)**** Pseudotime expression dynamics of core FLS2 signaling pathway genes across different cell types.


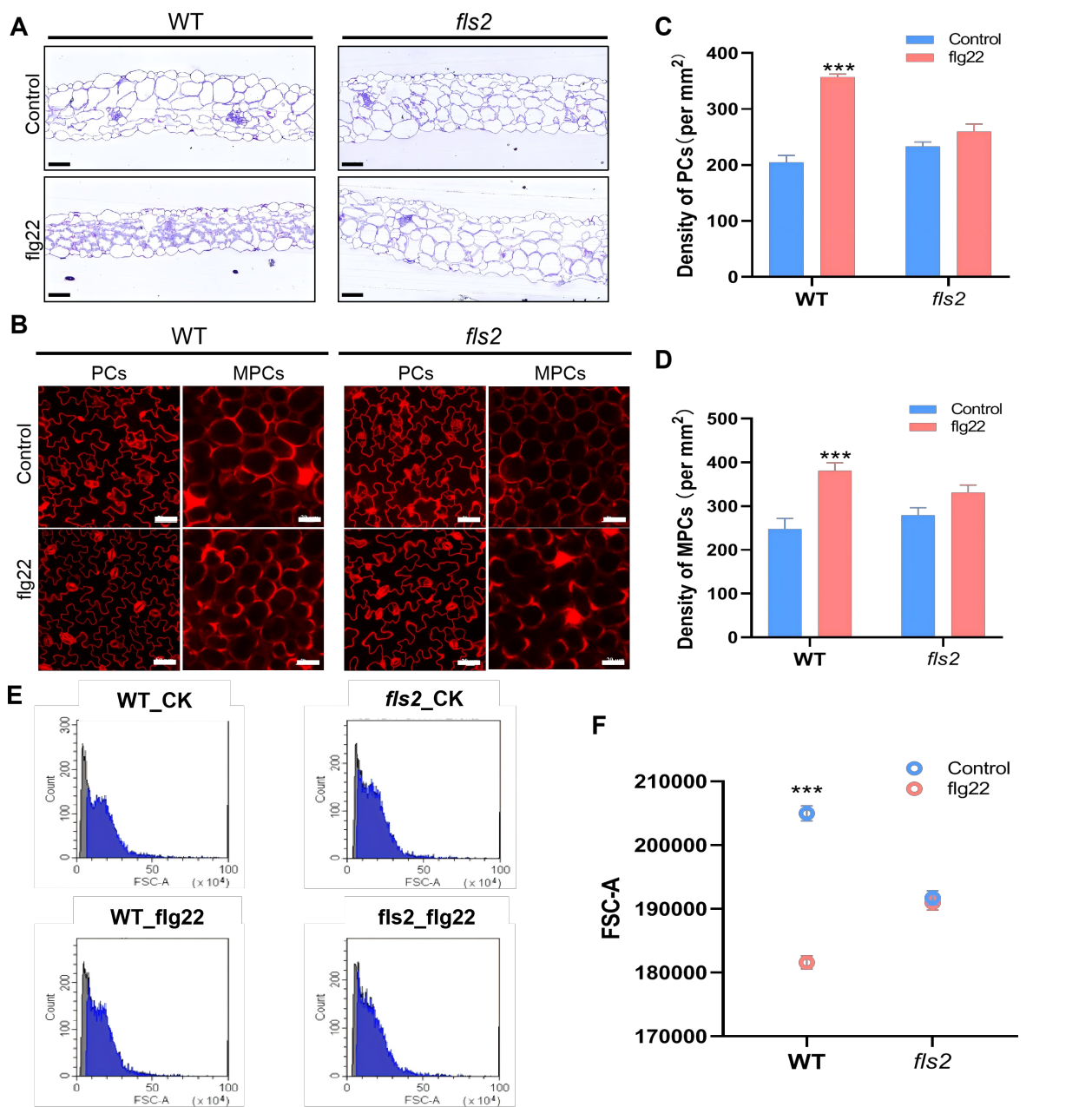


****Supplemental Figure S11.** Effects of flg22 treatment on epidermal and mesophyll cell development in *Arabidopsis* WT and *fls2* mutant seedlings.**

****(A)**** Semi-thin tissue sections (transverse view) of cotyledons from 3-day-old WT and *fls2* mutant seedlings after 3 days of control or flg22 treatment. Scale bar = 50 μm.
****(B)**** Confocal microscopy analysis of PCs and MPCs developmental phenotypes of cotyledons from 3-day-old WT and *fls2* mutant seedlings after 3 days of control or flg22 treatment. Scale bar = 50 μm.
****(C)**** Quantitative analysis of PC density in (B). Data are presented as mean ± SD (n = 3). Statistical significance between treatment and control was determined by one-way ANOVA followed by Tukey's test (***p<0.001).
****(D)**** Quantitative analysis of MPC density in (B). Data are presented as mean ± SD (n = 3). Statistical significance between treatment and control was determined by one-way ANOVA followed by Tukey's test (***p<0.001).
****(E)**** Flow cytometry histograms of cell size (FSC-A) of cotyledons from 3-day-old WT and *fls2* mutant seedlings after 3 days of control or flg22 treatment. The y-axis (count) represents cell number, while the x-axis (FSC-A) indicates forward scatter values proportional to cell size.
****(F)**** Quantification of cell size from (E). Data are presented as mean ± SD (n = 3). Statistical significance between treatment and control was determined by one-way ANOVA followed by Tukey's test (***p<0.001).


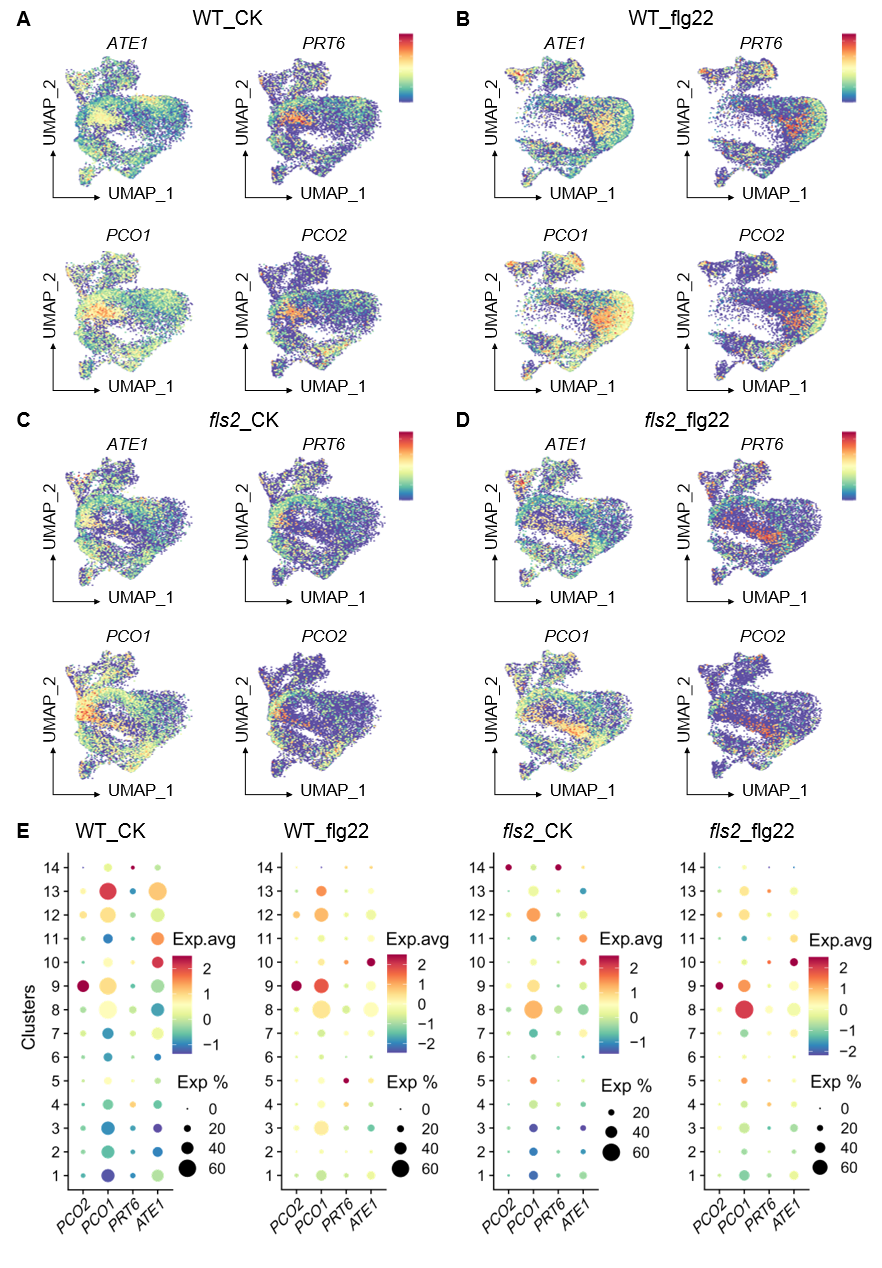


****Supplemental Figure S12.** Analysis of hypoxia-responsive gene expression patterns.**

**(A)-(D)** Feature plots depicting the spatial expression patterns of hypoxia-responsive genes (*ATE1*, *PRT6*, *PCO1*, and *PCO2*) under different conditions: WT_CK, WT_flg22, *fls2*_CK, and *fls2*_flg22.

**(E)** Dot plot summarizing the expression patterns and levels of these genes across distinct cell clusters within the WT_CK, WT_flg22, *fls2*_CK, and *fls2*_flg22 samples.


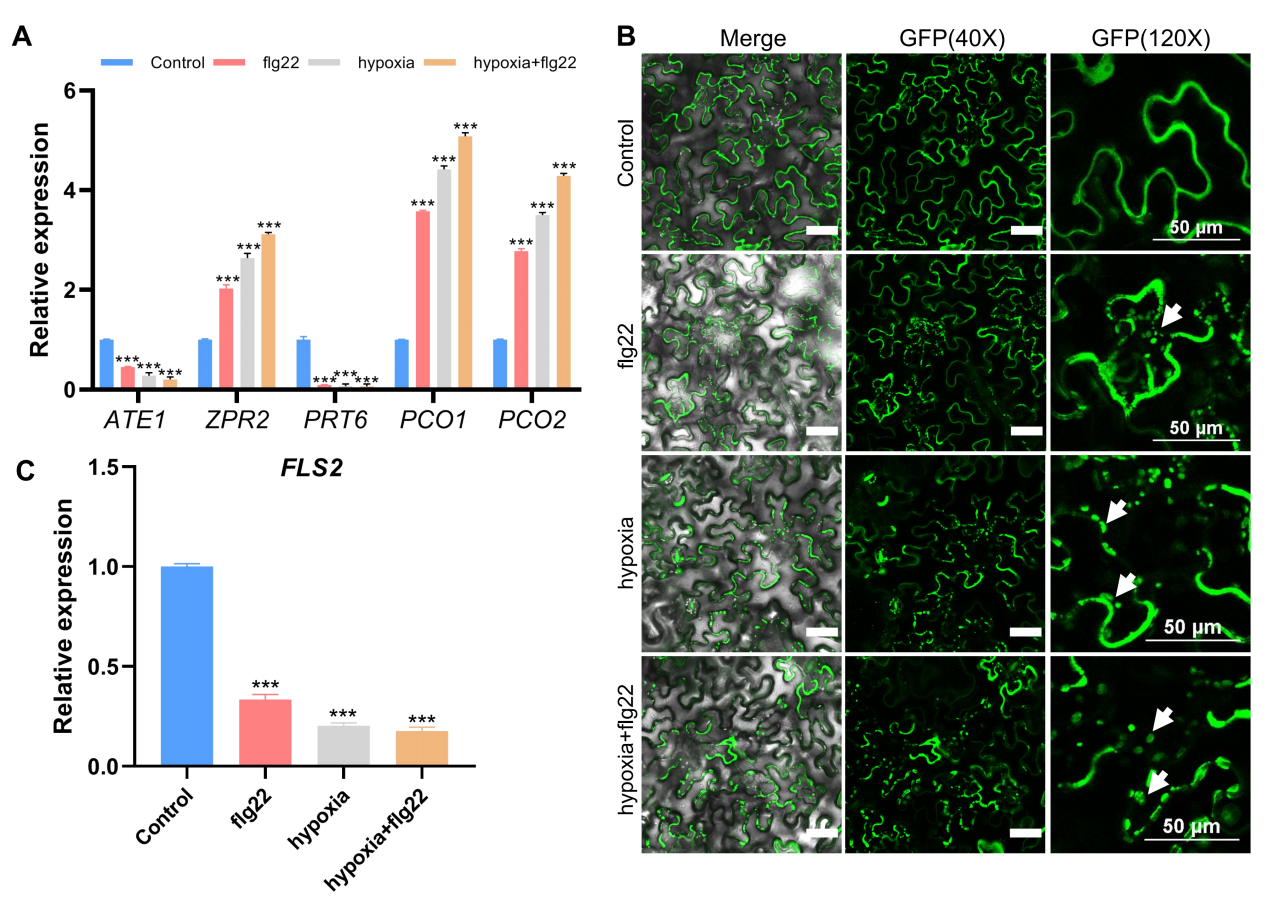


****Supplemental Figure S13.** Interaction between hypoxia signaling and flg22-triggered immune responses.**

****(A)**** Expression analysis of *ATE1*, *ZPR2*,*PRT6*,*PCO1*, and *PCO2* genes in cotyledons of WT seedlings treated with flg22, hypoxia, or a combination of hypoxia and flg22. Data are presented as mean ± SD (n = 3). Statistical significance between treatment and control was determined by one-way ANOVA followed by Tukey's test (***p<0.001).

****(B)**** Representative images showing the expression pattern of *FLS2p::FLS2-GFP*. GFP: green fluorescent protein; Merge: overlay of GFP and bright-field channels. Scale bar = 50 μm.

****(C)**** Relative expression levels of FLS2 in cotyledons of WT seedlings under flg22, hypoxia, or combined hypoxia and flg22 treatments. Data are presented as mean ± SD (n = 3). Statistical significance between treatment and control was determined by one-way ANOVA followed by Tukey's test (***p<0.001).


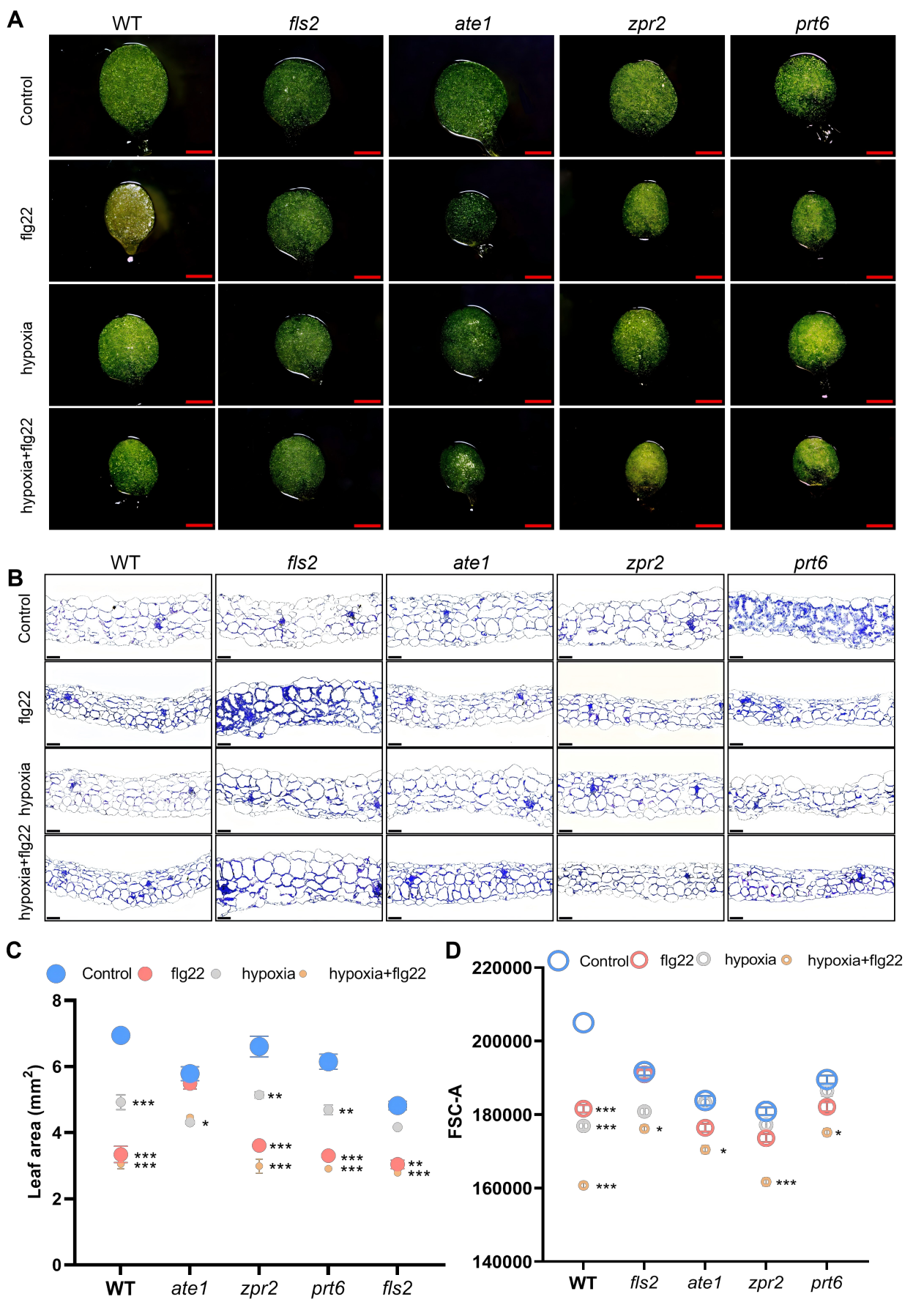


****Supplemental Figure S14.** Effects of flg22 and hypoxia treatments on cotyledon development in** WT, *fls2*, *ate1*, *prt6*, and*zpr2* seedlings**.**

Phenotypic analysis of cotyledons from 3-day-old WT, *fls2*, *ate1*, *prt6*, and*zpr2* seedlings following 3 days of treatment under control conditions, flg22 treatment, hypoxia, or combined hypoxia+flg22 treatment.

****(A)**** Cotyledon morphology. Scale bar = 1 mm.

****(B)**** Semi-thin cross-sections of cotyledons. Scale bar = 50 μm.

****(C)**** Quantification of cotyledon area in (A). Data are presented as mean ± SD (n = 3). Statistical significance between treatment and control within the same genotype was determined by one-way ANOVA followed by Tukey's test (***p<0.001).
****(D)**** Analysis of cotyledon cell size. Data are presented as mean ± SD (n = 3). Statistical significance between treatment and control within the same genetype was determined by one-way ANOVA followed by Tukey's test (***p<0.001).


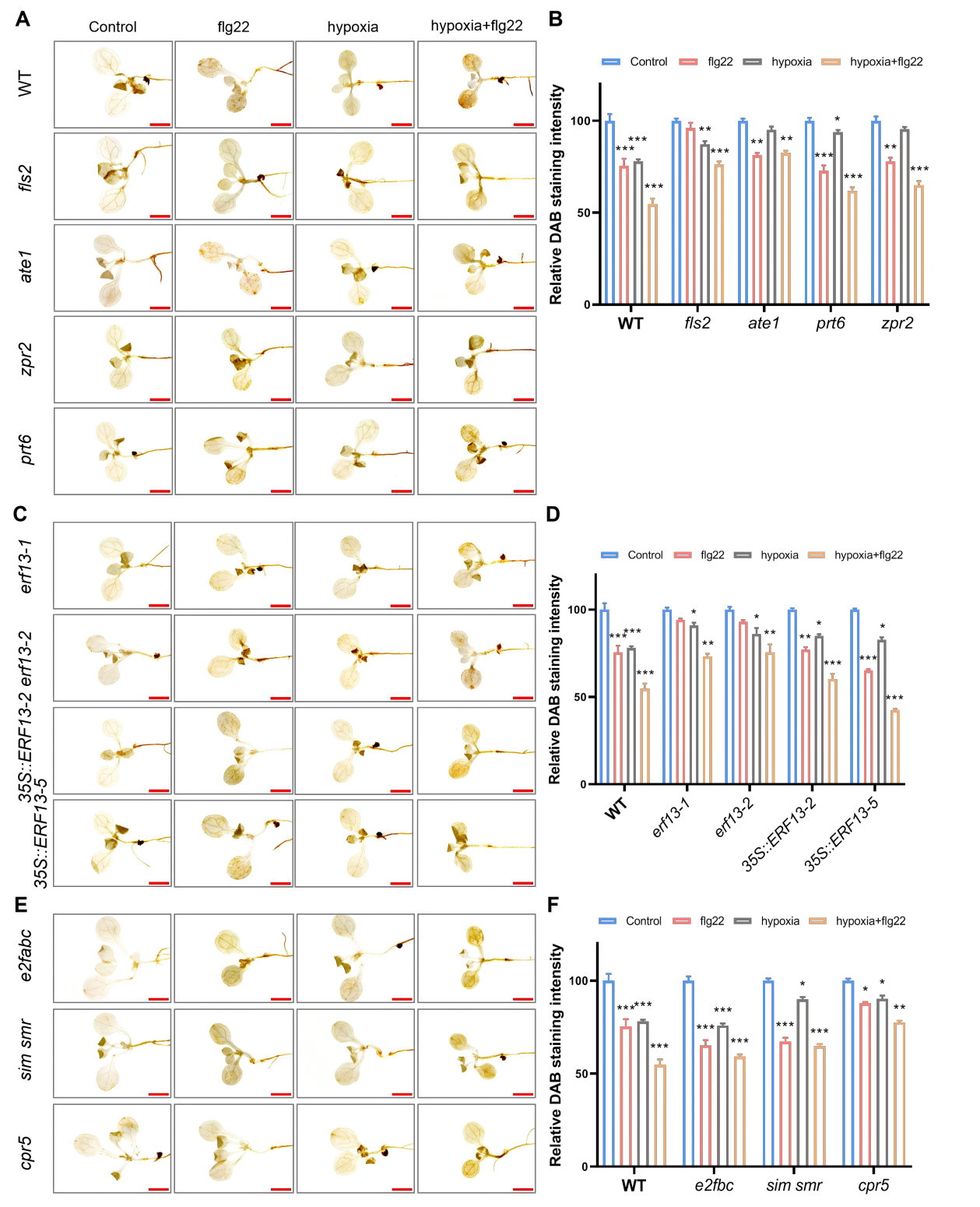


****Supplemental Figure S15.**** DAB staining **analysis of the effects of flg22 and hypoxia treatments on the** H_2_O_2_ **accumulation in** WT, mutants and transgenic seedlings**.**

**(A)** Histochemical detection of H_2_O_2_ accumulation in WT, *fls2*, *ate1*, *prt6*, and*zpr2* seedling via DAB staining, shown by the brown precipitate. The panel depicts a representative DAB staining image. Scale bar = 2 mm.

**(B)** Statistical analysis of the grayscale value indicating DAB staining intensity in (A). A lower grayscale value represents darker DAB staining and a higher level of H_2_O_2_ accumulation. Data are presented as mean ± SD (n = 3). The asterisk indicates a significant difference between the treatment group and the control group as determined by Student's *t*-test (*p < 0.05,**p < 0.01,***p < 0.001).

**(C)** Histochemical detection of H_2_O_2_ accumulation in *erf13-1*, *erf13-2*, *35S::ERF13-2*, and *35S::ERF13-5* and seedlings via DAB staining. The panel depicts a representative DAB staining image. Scale bar = 2 mm.

**(D)** Statistical analysis of the grayscale value indicating DAB staining intensity in (B). A lower grayscale value represents darker DAB staining and a higher level of H_2_O_2_ accumulation. Data are presented as mean ± SD (n = 3). The asterisk indicates a significant difference between the treatment group and the control group as determined by Student's *t*-test (*p < 0.05,**p < 0.01,***p < 0.001).

**(E)**Histochemical detection of H_2_O_2_ accumulation in *e2fabc*, *sim smr*, and *cpr5* seedlings via DAB staining. The panel depicts a representative DAB staining image. Scale bar = 2 mm.

**(F)** Statistical analysis of the grayscale value indicating DAB staining intensity in (E). A lower grayscale value represents darker DAB staining and a higher level of H_2_O_2_ accumulation. Data are presented as mean ± SD (n = 3). The asterisk indicates a significant difference between the treatment group and the control group as determined by Student's *t*-test (*p < 0.05,**p < 0.01,***p < 0.001).


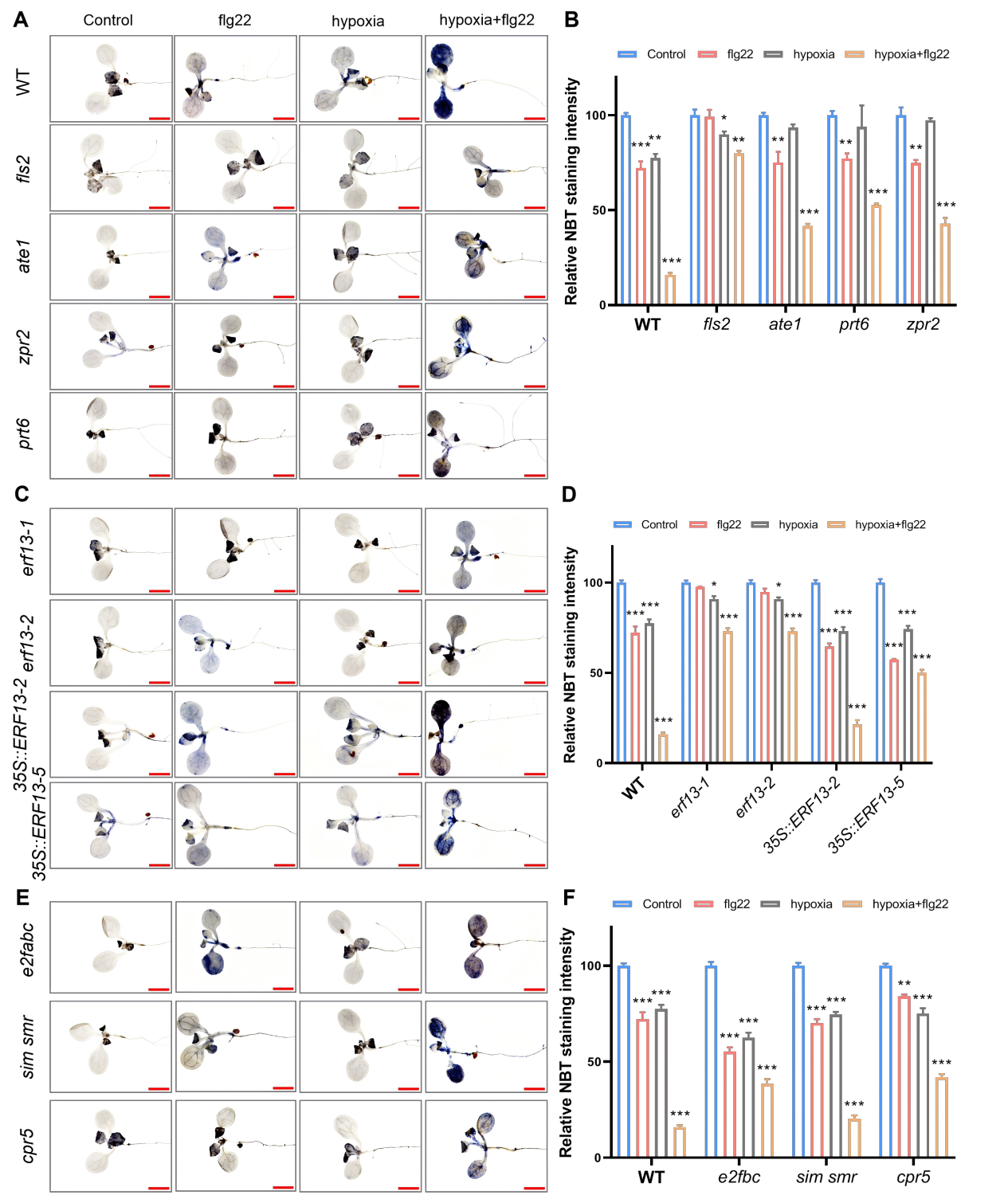


****Supplemental Figure S16.**** NBT staining **analysis of the effects of flg22 and hypoxia treatments on the** superoxide anion **accumulation in** WT, mutants and transgenic seedlings**.**

**(A)** Histochemical detection of superoxide anion accumulation in WT, *fls2*, *ate1*, *prt6*, and*zpr2* seedling via NBT staining, visualized as blue formazan deposits. The panel depicts a representative NBT staining image. Scale bar = 2 mm.

**(B)** Statistical analysis of the grayscale value indicating NBT staining intensity in (A). A lower grayscale value represents darker NBT staining and a higher level of superoxide anion accumulation. Data are presented as mean ± SD (n = 3). The asterisk indicates a significant difference between the treatment group and the control group as determined by Student's *t*-test (*p < 0.05,**p < 0.01,***p < 0.001).

**(C)** Histochemical detection of superoxide anion accumulation in *erf13-1*, *erf13-2*, *35S::ERF13-2*, and *35S::ERF13-5* seedlings via NBT staining. The panel depicts a representative NBT staining image. Scale bar = 2 mm.

**(D)** Statistical analysis of the grayscale value indicating NBT staining intensity in (C). A lower grayscale value represents darker NBT staining and a higher level of superoxide anion accumulation. Data are presented as mean ± SD (n = 3). The asterisk indicates a significant difference between the treatment group and the control group as determined by Student's *t*-test (*p < 0.05,***p < 0.001).

**(E)** Histochemical detection of superoxide anion accumulation in *e2fabc*, *sim smr*, and *cpr5* seedlings via NBT staining. The panel depicts a representative NBT staining image. Scale bar = 2 mm.

**(F)** Statistical analysis of the grayscale value indicating NBT staining intensity in (E). A lower grayscale value represents darker NBT staining and a higher level of superoxide anion accumulation. Data are presented as mean ± SD (n = 3). The asterisk indicates a significant difference between the treatment group and the control group as determined by Student's *t*-test (**p < 0.01,***p < 0.001).


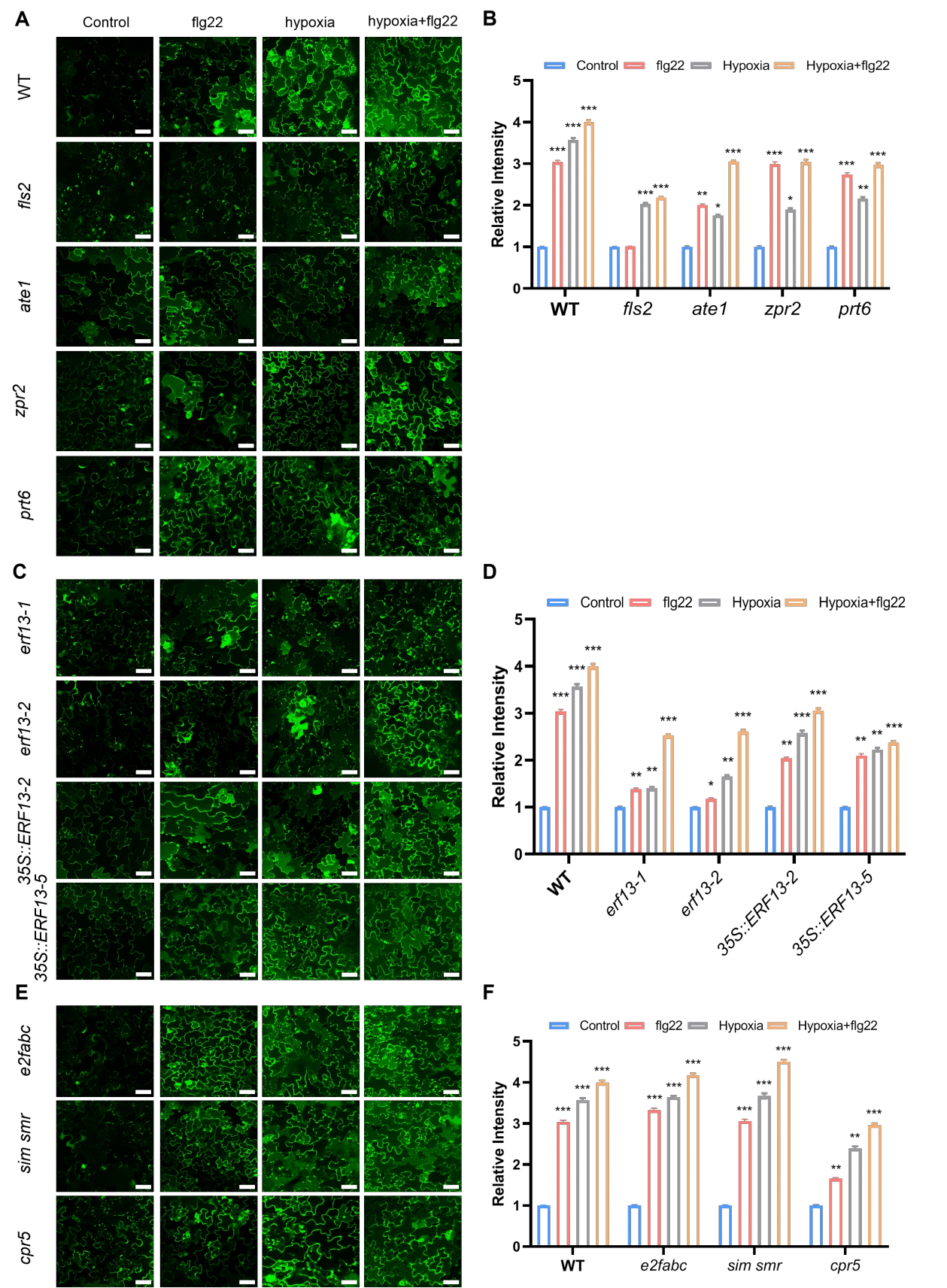


****Supplemental Figure S17.**** H_2_CFDA staining **analysis of the effects of flg22 and hypoxia treatments on the ROS accumulation in** WT, mutants and transgenic seedlings**.**

**(A)** Detection of intracellular ROS levels by H_2_CFDA staining. Representative images show the fluorescence of the oxidized DCF probe following H_2_CFDA staining in WT, *fls2*, *ate1*, *prt6*, and*zpr2* seedlings under control and stress conditions. Scale bar, 50 µm.

**(B)** Quantification of DCF fluorescence intensity. Fluorescence intensity was measured from images as shown in (A). Data are presented as mean ± SD (n = 3) Statistical significance was determined by Student's *t*-test, comparing the stress-treated samples with their respective controls (*p < 0.05,**p < 0.01,***p < 0.001).

**(C)** Detection of intracellular ROS levels by H_2_CFDA staining. Representative images show the fluorescence of the oxidized DCF probe following H_2_CFDA staining in *erf13-1*, *erf13-2*, *35S::ERF13-2*, and *35S::ERF13-5* seedlings under control and stress conditions. Scale bar, 50 µm.

**(D)** Quantification of DCF fluorescence intensity. Fluorescence intensity was measured from images as shown in (C) . Data are presented as mean ± SD (n = 3) Statistical significance was determined by Student's *t*-test, comparing the stress-treated samples with their respective controls (*p < 0.05,**p < 0.01,***p < 0.001).

**(E)** Detection of intracellular ROS levels by H_2_CFDA staining. Representative images show the fluorescence of the oxidized DCF probe following H_2_CFDA staining in *e2fabc*, *sim smr*, and *cpr5* seedlings under control and stress conditions. Scale bar, 50 µm.

**(F)** Quantification of DCF fluorescence intensity. Fluorescence intensity was measured from images as shown in (E) . Data are presented as mean ± SD (n = 3) Statistical significance was determined by Student's *t*-test, comparing the stress-treated samples with their respective controls (**p < 0.01,***p < 0.001).


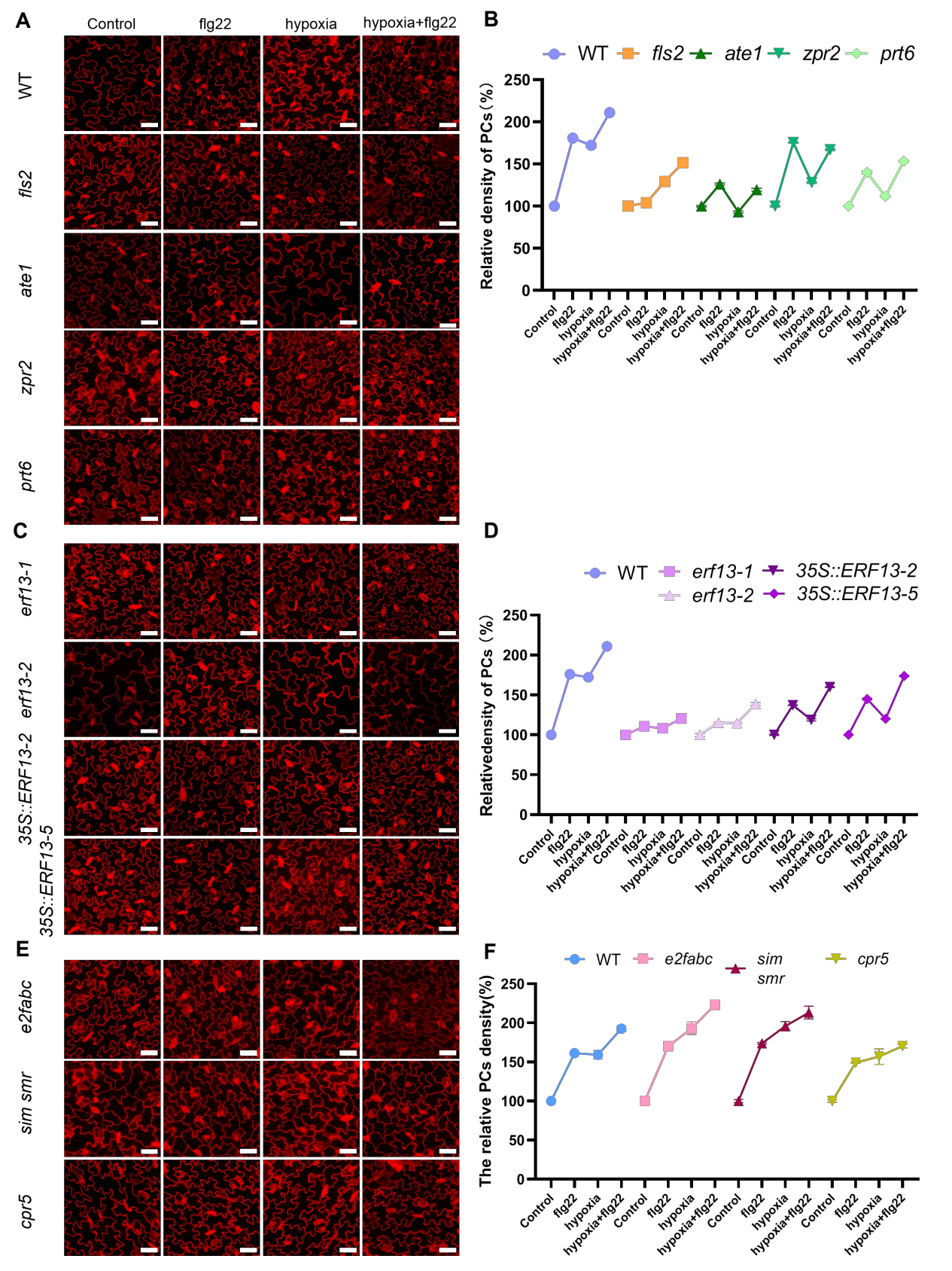


****Supplemental Figure S18.** Effects of immune and hypoxia treatments on PCs in** WT, mutants and transgenic seedlings**.**

****(A)**** Confocal microscopy analysis of PC developmental phenotypes of WT, *fls2*, *ate1*, *prt6*, and *zpr2* seedlings under control conditions, flg22 treatment, hypoxia, or combined hypoxia+flg22 treatment. Scale bar = 50 μm.

****(B)**** Statistical analysis of relative PC density in (A). Data are presented as mean ± SD (n = 3).
****(C)**** Confocal microscopy analysis of PC developmental phenotypes of *erf13-1*, *erf13-2*, *35S::ERF13-2*, and *35S::ERF13-5* seedlings under control conditions, flg22 treatment, hypoxia, or combined hypoxia+flg22 treatment. Scale bar = 50 μm.

****(D)**** Statistical analysis of relative PC density in (C). Data are presented as mean ± SD (n = 3).

****(E)**** Confocal microscopy analysis of PC developmental phenotypes of *e2fabc*, *sim smr*, and *cpr5* seedlings under control conditions, flg22 treatment, hypoxia, or combined hypoxia+flg22 treatment. Scale bar = 50 μm.

****(F)**** Statistical analysis of relative PC density in (E). Data are presented as mean ± SD (n = 3).


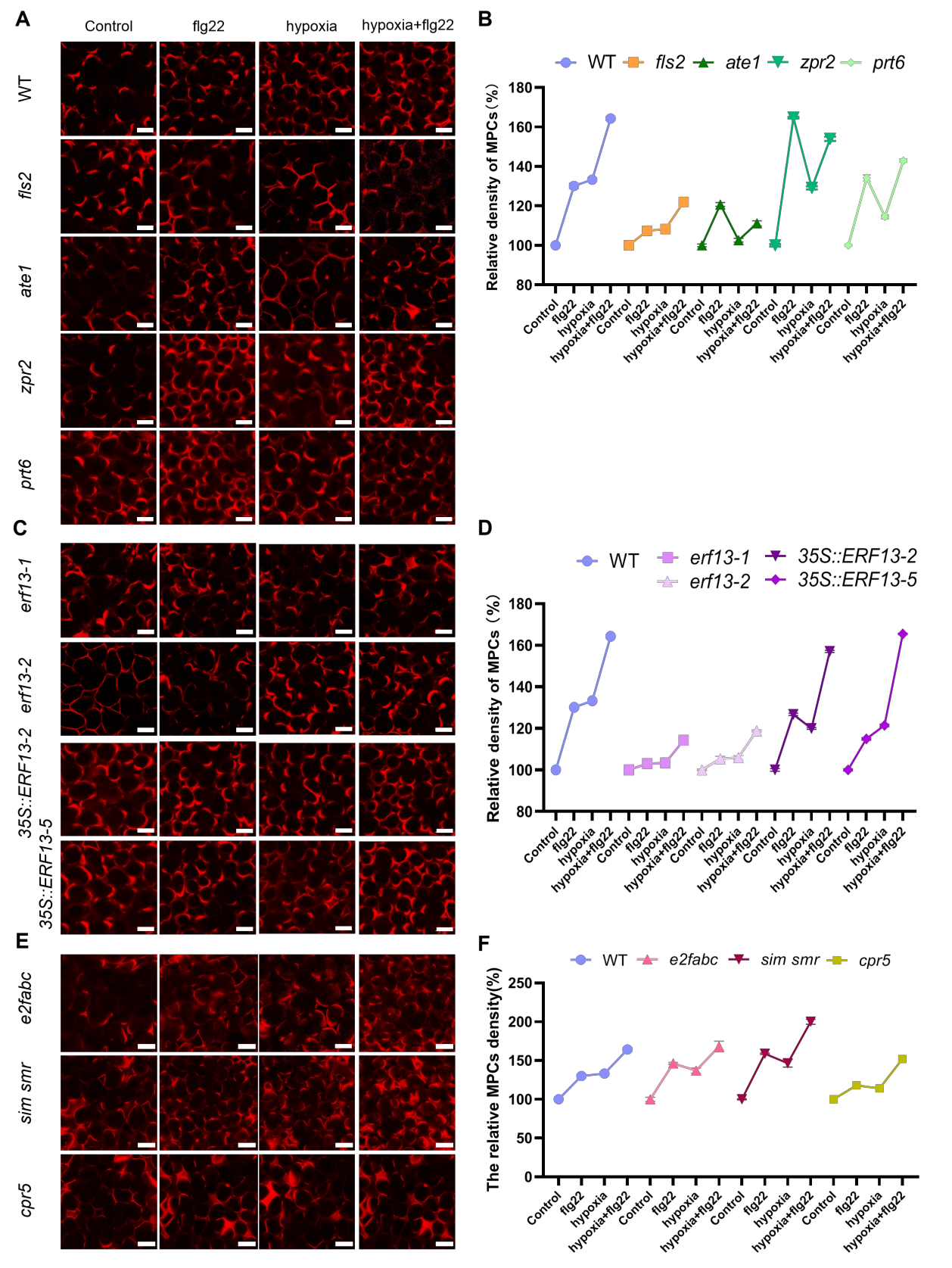


****Supplemental Figure S19.** Effects of immune and hypoxia treatments on MPCs in** WT, mutants and transgenic seedlings**.**

****(A)**** Confocal microscopy analysis of MPC developmental phenotypes of WT, *fls2*, *ate1*, *prt6*, and *zpr2* seedlings under control conditions, flg22 treatment, hypoxia, or combined hypoxia+flg22 treatment. Scale bar = 50 μm.

****(B)**** Statistical analysis of relative MPC density in (A). Data are presented as mean ± SD (n = 3).
****(C)**** Confocal microscopy analysis of MPC developmental phenotypes of *erf13-1*, *erf13-2*, *35S::ERF13-2*, and *35S::ERF13-5* seedlings under control conditions, flg22 treatment, hypoxia, or combined hypoxia+flg22 treatment. Scale bar = 50 μm.

****(D)**** Statistical analysis of relative MPC density in (C). Data are presented as mean ± SD (n = 3).

****(E)**** Confocal microscopy analysis of MPC developmental phenotypes of *e2fabc*, *sim smr*, and *cpr5* seedlings under control conditions, flg22 treatment, hypoxia, or combined hypoxia+flg22 treatment. Scale bar = 50 μm.

****(F)**** Statistical analysis of relative MPC density in (E). Data are presented as mean ± SD (n = 3).


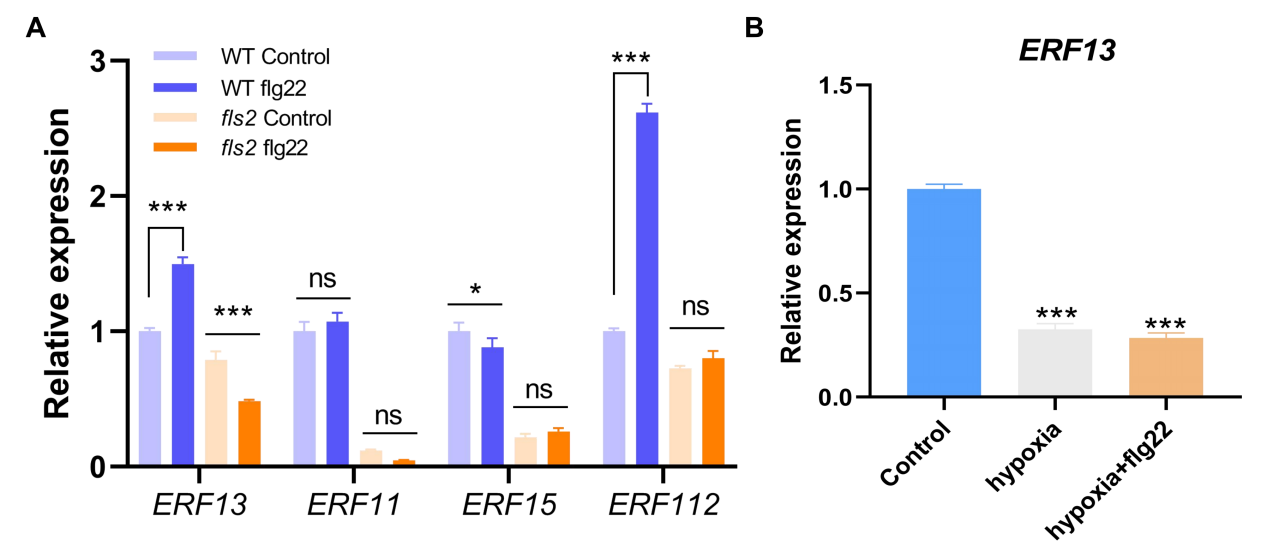


****Supplemental Figure S20.** Analysis of *ERF genes* expression patterns.**

****(A)**** Expression profiles of *ERF13*, *ERF11*, *ERF15*, *ERF112* in *fls2* and WT seedlings under control and flg22 treatment conditions. Data are presented as mean ± SD (n = 3). Statistical significance was determined by one-way ANOVA followed by Tukey's test. Significant differences between treatment and control are indicated by asterisks (*p<0.05, ***p<0.001).

****(B)**** *ERF13* expression levels in WT seedlings subjected to hypoxia and combined hypoxia+flg22 treatments. Data are presented as mean ± SD (n = 3). Statistical significance was determined by one-way ANOVA followed by Tukey's test. Significant differences between treatment and control are indicated by asterisks (***p<0.001).


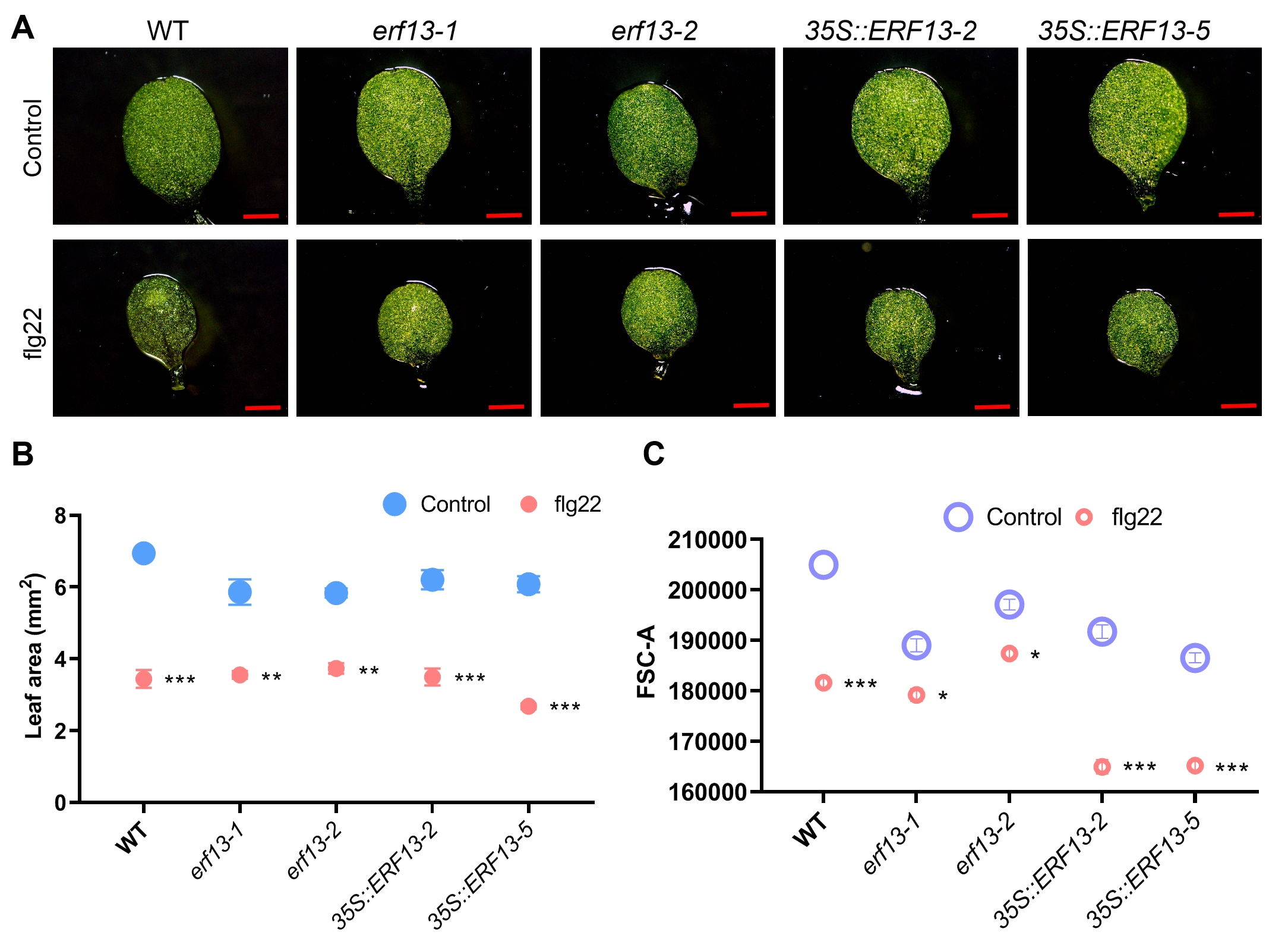


**Supplemental Figure S21.** **Effects of flg22 treatment on cotyledon development in** WT, *erf13-1*, *erf13-2*, *35S::ERF13-2*, and *35S::ERF13-5* seedlings**.**

Phenotypic analysis of cotyledons from 3-day-old WT, *erf13-1*, *erf13-2*, *35S::ERF13-2*, and *35S::ERF13-5* seedlings following 3 days of control or flg22 treatment.

****(A)**** Representative cotyledon images of WT, *erf13-1*, *erf13-2*, *35S::ERF13-2*, and *35S::ERF13-5* seedlings. Scale bar = 1 mm.
****(B)**** Quantification of cotyledon area of seedlings in (A). Data are presented as mean ± SD (n = 3). Significant differences between treatment and control within the same genetype were determined by two-way ANOVA followed by Tukey's test and are denoted by asterisks: **p < 0.01, ***p < 0.001.
****(C)**** Analysis of cotyledon cell size was performed using flow cytometry histograms (FSC-A) of the seedlings shown in (A). Data are presented as mean ± SD (n = 3). Significant differences between treatment and control within the same genotype were determined by two-way ANOVA followed by Tukey's test and are denoted by asterisks: *p < 0.05, ***p < 0.001.


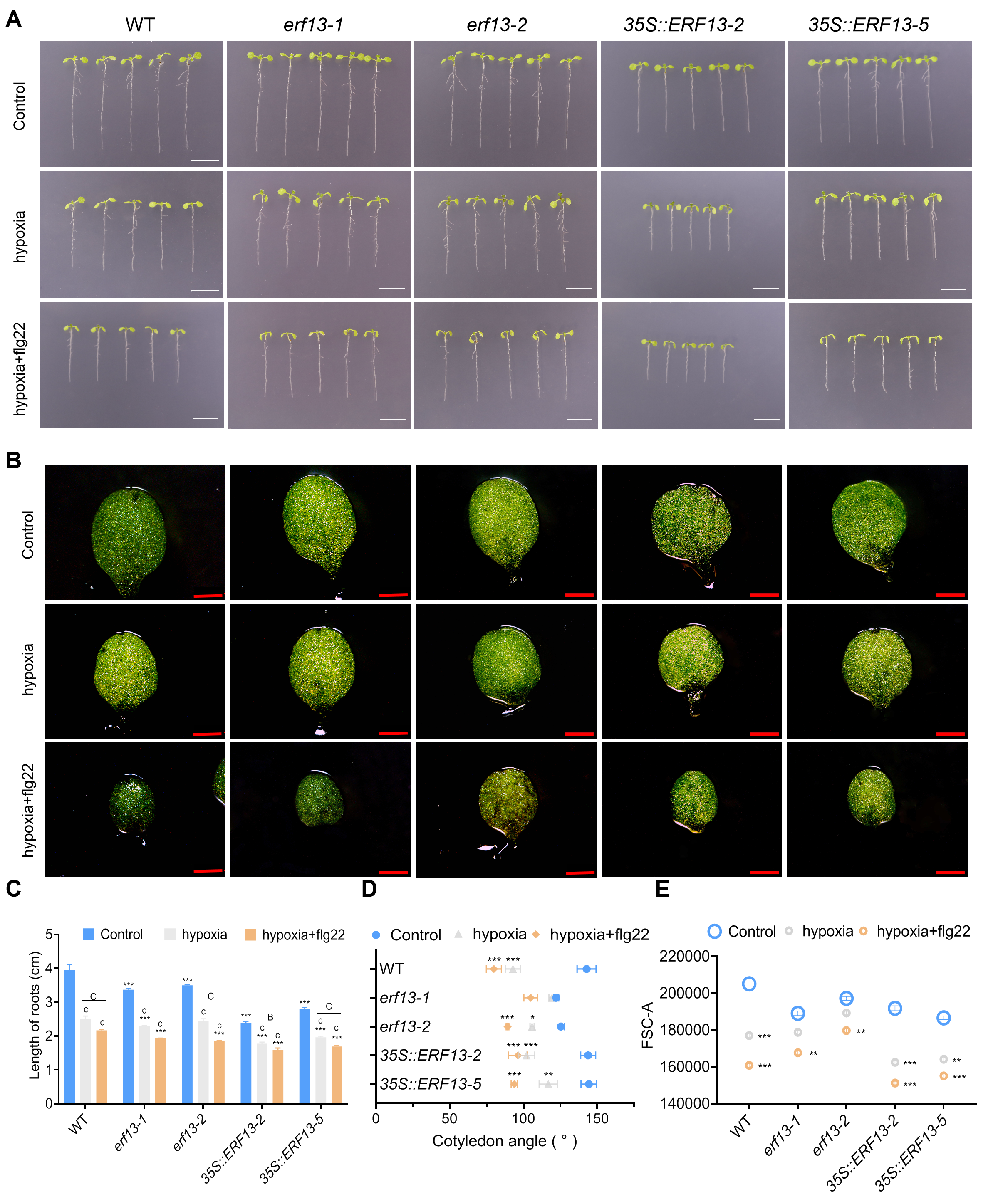


**Supplemental Figure S22.** **Effects of hypoxia and combined hypoxia+flg22 treatments on** WT, *erf13-1*, *erf13-2*, *35S::ERF13-2*, and *35S::ERF13-5* seedlings**.**

Phenotypic analysis of 3-day-old WT, *erf13-1*, *erf13-2*, *35S::ERF13-2*, and *35S::ERF13-5* seedlings following 3 days of treatment under control conditions, hypoxia, or combined hypoxia+flg22 treatment.

****(A)**** Whole seedling morphology of WT, *erf13-1*, *erf13-2*, *35S::ERF13-2*, and *35S::ERF13-5* seedlings. Scale bar = 1 cm.
****(B)**** Cotyledon phenotype of WT, *erf13-1*, *erf13-2*, *35S::ERF13-2*, and *35S::ERF13-5* seedlings. Scale bar = 1 mm.
****(C)**** Quantitative analysis of root length of seedlings in (A).Data are presented as mean ± SD (n = 3). Statistical significance was determined by two-way ANOVA followed by Tukey's test. Significant differences between mutants and WT are indicated by asterisks (***p<0.001). Significant differences between treatment groups and controls are denoted by lowercase letters (cp<0.001). Significant differences among treatment groups are marked by uppercase letters (Bp<0.01, Cp<0.001).

****(D)**** Measurement of cotyledon opening angle of seedlings in (A). Data are presented as mean ± SD (n = 3). Significant differences between treatment and control within the same genetype were determined by two-way ANOVA followed by Tukey's test and are denoted by asterisks: *p < 0.05, **p < 0.01, ***p < 0.001.
****(E)**** Analysis of cotyledon cell size was performed using flow cytometry histograms (FSC-A) of the seedlings shown in (A). Data are presented as mean ± SD (n = 3). Significant differences between treatment and control within the same genetype were determined by two-way ANOVA followed by Tukey's test and are denoted by asterisks: **p < 0.01, ***p < 0.001.


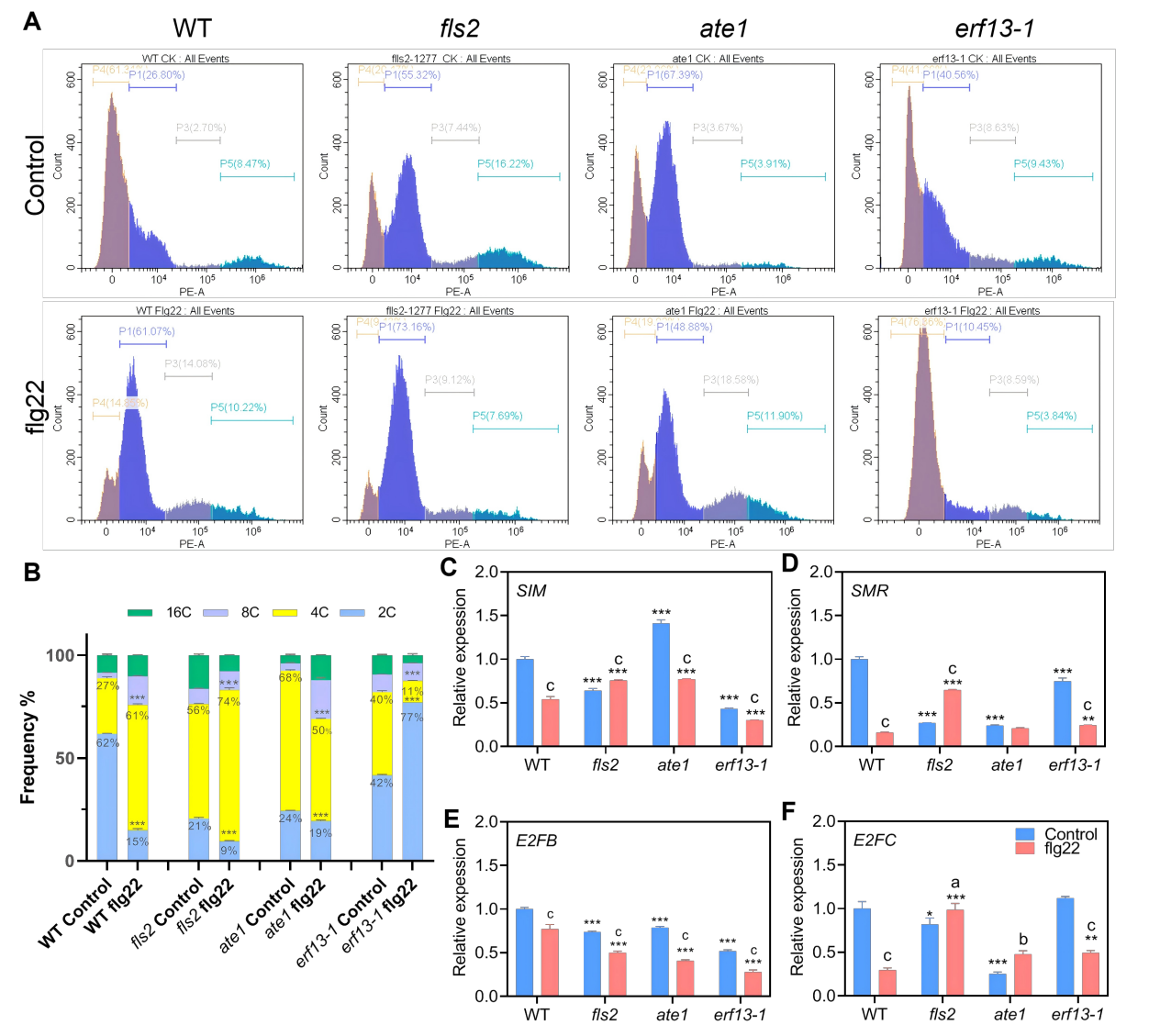


****Supplemental Figure S23.** Impact of the flg22 immune response on cotyledon cell cycle.**

1. Frequency distribution histograms of chromosomal ploidy in cotyledon cells of WT, *fls2*, *ate1*, and *erf13-1*seedlings before and after flg22 treatment. The x-axis (PE-A) represents the fluorescence intensity area under the curve for the nucleic acid dye propidium iodide (PI), reflecting DNA content. The y-axis (Count) indicates cell number.
   ****(B)**** Percentage composition of chromosome ploidy. Data are presented as mean ± SD (n=3). Statistical significance between the treatment and control groups was determined by one-way ANOVA followed by Tukey's test: ***p < 0.001.

****(C)**** Expression levels of cell cycle regulatory genes *SIM*,*SMR*, *E2FB*, and *E2FC*.

Data are presented as mean ± SD (n = 3). Significant differences were determined by two-way ANOVA followed by Tukey's test. Asterisks denote significant differences between mutants and WT within the same treatment: *p < 0.05, **p < 0.01, ***p < 0.001. Lowercase letters indicate significant differences between treatment groups and their respective controls: ap < 0.05, bp < 0.01, cp < 0.001.


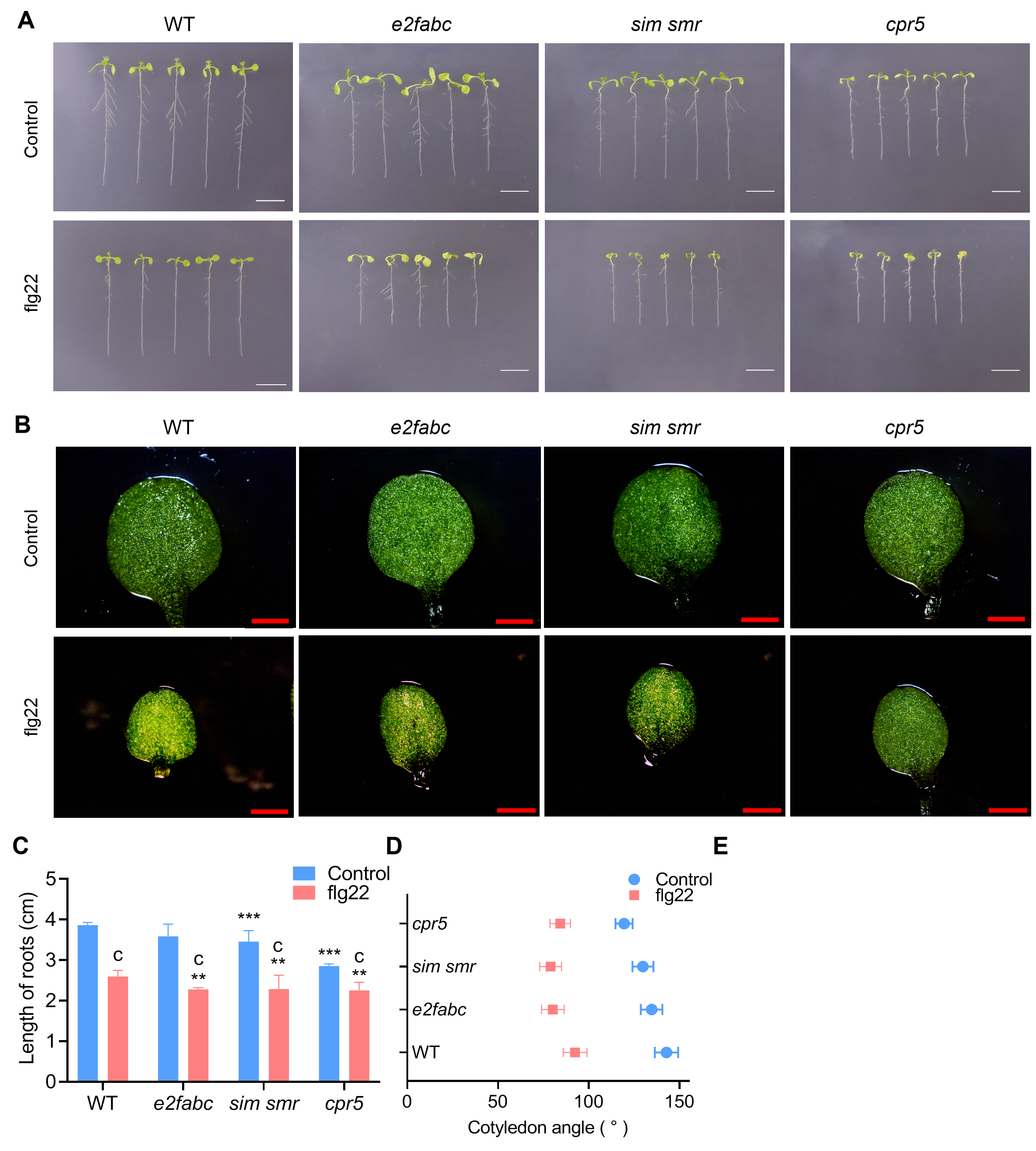


****Supplemental Figure S24.** Effects of flg22 treatment on seedlings of cell cycle regulatory mutants.**

Phenotypic analysis of 3-day-old WT, *e2fabc*, *sim smr*, and *cpr5* seedlings following 3 days of control or flg22 treatment.

****(A)**** Representative whole-seedling images of 3-day-old WT, *e2fabc*, *sim smr*, and *cpr5* seedlings. Scale bar = 1 cm.

****(B)**** Representative cotyledon images of 3-day-old WT, *e2fabc*, *sim smr*, and *cpr5* seedlings. Scale bar = 1 mm.
****(C)**** Root length quantification of seedlings in (A).Data are presented as mean ± SD (n = 3). Significant differences were determined by two-way ANOVA followed by Tukey's test. Asterisks denote significant differences between mutants and WT within the same treatment: **p < 0.01, ***p < 0.001. Lowercase letters indicate significant differences between flg22-treated groups and their respective untreated controls: cp < 0.001.

****(D)**** Cotyledon angle measurement of seedlings in (A). Data are presented as mean ± SD (n = 3). Significant differences between treatment and control within the same genetype were determined by two-way ANOVA followed by Tukey's test and are denoted by asterisks: **p < 0.01, ***p < 0.001.
****(E)**** Cotyledon area quantification of seedlings in (B). Data are presented as mean ± SD (n = 3). Significant differences between treatment and control within the same genetype were determined by two-way ANOVA followed by Tukey's test and are denoted by asterisks: **p < 0.01, ***p < 0.001.


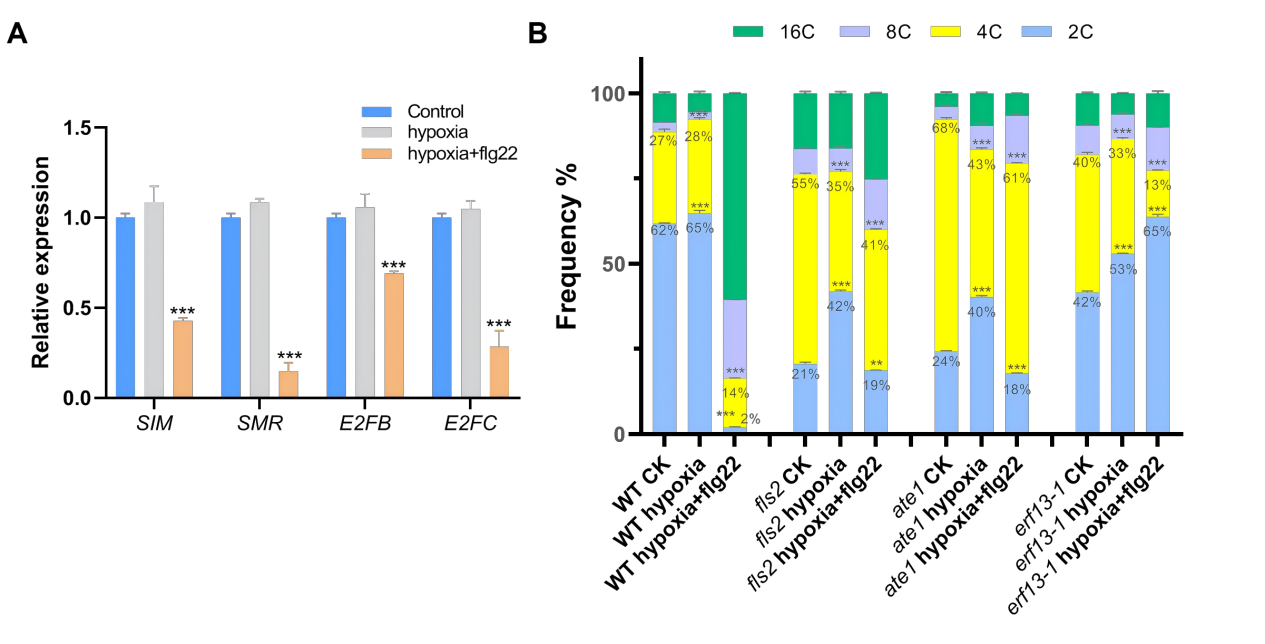


****Supplemental Figure S25.** Impact of hypoxia and hypoxia+flg22 co-treatment on cotyledon cell cycle.**

1. Expression analysis of cell cycle regulatory genes *SIM*, *SMR*, *E2FB*, and *E2FC* in WT cotyledons before and after hypoxia or hypoxia+flg22 co-treatment. Data are presented as mean ± SD (n = 3). Significant differences were determined by two-way ANOVA followed by Tukey's test. Asterisks denote significant differences between mutants and WT within the same treatment: ***p < 0.001.

****(B)**** Percentage composition of chromosomal ploidy states in cotyledon cells of WT, *fls2*, *ate1*, and *erf13-1*seedlings following treatment. Data are presented as mean ± SD (n=3). Statistical significance between the treatment and control groups was determined by one-way ANOVA followed by Tukey's test: ***p < 0.001.


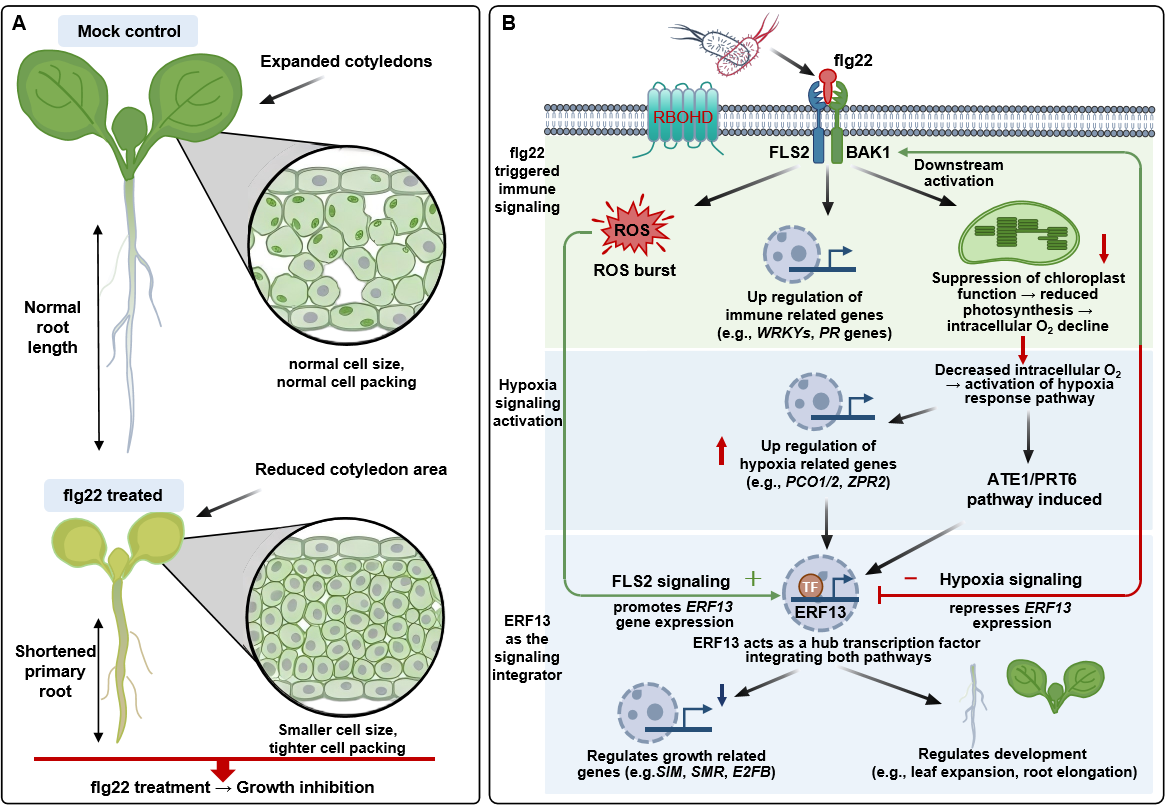


****Supplemental Figure S26.**** A schematic model illustrating the mechanism by which FLS2 and hypoxia signaling regulate cell development and immune responses.

****(A)**** Schematic representation showing that when plant seedlings are treated with flg22, leaf development is inhibited, and cell growth is restricted.

****(B)**** Upon perception of flg22 by the FLS2/BAK1 receptor complex, canonical immune signaling is activated, including a ROS burst (mediated by RBOHD and MPK3/6) and upregulation of defense-related genes (e.g., *WRKYs*, *PR* genes). Concurrently, immune signaling suppresses chloroplast function, leading to reduced photosynthesis and a decline in intracellular O_2_ levels. This decrease in O_2_ activates the hypoxia response pathway, including the ATE1/PRT6 N-degron pathway and upregulation of hypoxia-responsive genes. The transcription factor ERF13 acts as an integrative hub: its expression is promoted by FLS2 signaling but repressed by hypoxia signaling, which conversely enhances *FLS2*expression. ERF13, in turn, directly regulates key cell cycle and growth-related genes (e.g., *SIM*, *SMR*, *E2FB*), ultimately orchestrating the inhibition of developmental processes such as leaf expansion and root elongation.
